# Exploring Novel Nitrofuryl‐1,3,4‐Thiadiazole‐Based Derivatives: Design, Synthesis, and Evaluation of In Vitro Leishmanicidal and Trypanocidal Activity

**DOI:** 10.1002/ardp.70227

**Published:** 2026-03-22

**Authors:** Alireza Mousavi, Martina Slapničková, Maryam Norouzbahari, Sarah D'Alessandro, Parham Foroumadi, Fariba Peytam, Federica Perego, Eva Doleželová, Zahra Emamgholipour, Maliheh Barazandeh Tehrani, Seyed Esmaeil Sadat‐Ebrahimi, Elahe Hosseinzadeh, Elmira Meghrazi Ahadi, Loghman Firoozpour, Hamidreza Bijanzadeh, Nicoletta Basilico, Alena Zíková, Alireza Foroumadi

**Affiliations:** ^1^ International Campus‐School of Pharmacy Tehran University of Medical Sciences Tehran Iran; ^2^ Department of Medicinal Chemistry, Faculty of Pharmacy Tehran University of Medical Sciences Tehran Iran; ^3^ Institute of Parasitology, Biology Centre, Czech Academy of Sciences Ceske Budejovice Czech Republic; ^4^ Faculty of Pharmacy Final International University Girne TRNC Turkey; ^5^ Department of Pharmacological and Biomolecular Sciences “Rodolfo Paoletti” University of Milan Milan Italy; ^6^ Drug Design and Development Research Centre, The Institute of Pharmaceutical Sciences Tehran University of Medical Sciences Tehran Iran; ^7^ Department of Biomedical, Surgical and Dental Sciences University of Milan Milan Italy; ^8^ Department of Environmental Sciences, Faculty of Natural Resources and Marine Sciences Tarbiat Modares University Tehran Iran

**Keywords:** apoptosis, cytostatic, Leishmania, nitrofuran, Trypanosoma

## Abstract

Neglected tropical diseases remain a major global health challenge, highlighting the need for new antiparasitic agents. In this study, a series of substituted 1‐[5‐(5‐nitrofuran‐2‐yl)‐1,3,4‐thiadiazol‐2‐yl]piperidine‐4‐carboxamides was designed, synthesized, and evaluated for in vitro antileishmanial and antitrypanosomal activity. Compound **18** emerged as the most promising derivative, showing submicromolar activity against all tested parasites with acceptable selectivity toward THP‐1 cells. Mechanistic studies in *T. b. brucei* bloodstream cells revealed a reversible cytostatic effect rather than apoptosis, and assessment of cellular and mitochondrial ROS levels indicated that oxidative stress was not a primary contributor to activity. In silico ADME analysis supported the drug‐likeness of the synthesized compounds. Taken together, these findings identify **18** as a valuable lead for further antiparasitic drug development.

## Graphical abstract

In a series of substituted 1‐[5‐(5‐nitrofuran‐2‐yl)‐1,3,4‐thiadiazol‐2‐yl]piperidine‐4‐carboxamides evaluated for in vitro antileishmanial and antitrypanosomal activity, compound **18** emerged as the most promising derivative, showing submicromolar anti‐parasitic effects targeting diverse *Leishmania* and *Trypanosoma* species and acceptable selectivity toward THP‐1 cells.

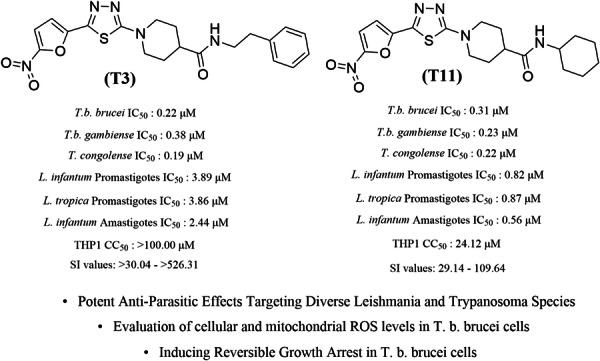

## 1 Introduction

Neglected tropical diseases (NTDs) comprise a group of approximately 20 conditions that collectively affect more than one billion individuals worldwide, predominantly in resource‐limited regions of Africa, Asia, and the Americas [1]. These diseases disproportionately burden vulnerable populations and are associated with severe health consequences as well as profound social and economic impacts, frequently leading to chronic disability and reduced quality of life [2]. Among the pathogens responsible for NTDs, trypanosomatid parasites are the etiological agents of several major infections, including leishmaniasis, human African trypanosomiasis (HAT), and American trypanosomiasis (Chagas disease) [3].

Leishmaniasis is caused by multiple *Leishmania* species and remains a serious public health concern in tropical and subtropical areas. Transmission occurs through the bite of infected phlebotomine sand flies, followed by intracellular proliferation of the parasites within host macrophages [4]. The disease manifests as cutaneous, mucocutaneous, or visceral forms, with visceral leishmaniasis being life‐threatening in the absence of treatment. Current therapeutic options are limited to a small number of agents, such as pentavalent antimonials, amphotericin B, paromomycin, and miltefosine, whose clinical utility is frequently compromised by toxicity, resistance development, high treatment costs, and poor patient adherence [5].

Human African trypanosomiasis, commonly referred to as sleeping sickness, is caused by subspecies of *Trypanosoma brucei* that can cross the blood–brain barrier and infecting the central nervous system [6]. The disease exists in two epidemiological forms: a chronic infection caused by *T. b. gambiense* in West and Central Africa and a more acute form caused by *T. b. rhodesiense* in Eastern and Southern Africa. Disease progression involves an initial hemolymphatic phase followed by a meningoencephalitic stage marked by neurological symptoms [7]. Available treatments, including pentamidine, suramin, melarsoprol, eflornithine, nifurtimox, and fexinidazole, are selected according to disease stage and parasite species but remain limited by toxicity, logistical challenges, and incomplete efficacy, highlighting the urgent need for safer and more effective therapies [8].

**Figure 1:**
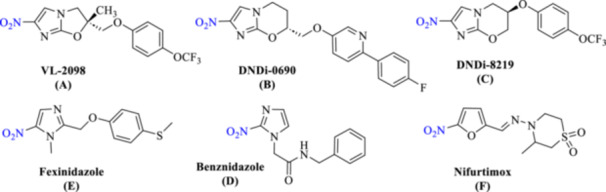
Structures of antitrypanosomal agents bearing nitro functionality.

Nitro‐containing compounds have long been recognized as effective scaffolds in antitrypanosomal drug discovery, with their use dating back to the 1960s. Several representative agents incorporating this functional group are shown in Figure 1 [9, 10]. Building on the success of nitro‐based antitubercular drugs such as delamanid and pretomanid, several nitroimidazole–oxazole and nitroimidazole–oxazine derivatives have been advanced as clinical candidates for leishmaniasis treatment [11]. Notable examples include VL‐2098, DNDi‐0690, and DNDi‐8219. Although VL‐2098 displayed strong efficacy against both acute and chronic *Leishmania donovani* infections in animal models, its further development was limited by unfavorable toxicological findings. DNDi‐0690 was subsequently optimized to address safety and solubility concerns and demonstrated efficacy against intracellular *Leishmania infantum* and *L. donovani*, ultimately progressing to phase I clinical evaluation [10]. DNDi‐8219, identified through screening of a pretomanid‐derived analogue library, also showed pronounced activity in a murine *L. donovani* model [12].

More recently, 5‐nitroimidazole derivatives have emerged as important therapeutic agents for human African trypanosomiasis [8]. Among them, fexinidazole represents a breakthrough, receiving approval in July 2021 as the first fully oral treatment effective against both early and late stages of *T. b. gambiense* infection [13]. Related nitro‐based scaffolds are also present in established antiparasitic drugs, including for Chagas disease. In addition, nifurtimox, a 5‐nitrofuran derivative, is used in Chagas disease therapy and has been approved by the WHO as part of the nifurtimox–eflornithine combination therapy (NECT) for HAT [14].

In addition to nitroheterocyclic backbones, the 1,3,4‐thiadiazole moiety is recognized as a privileged scaffold in medicinal chemistry and has attracted considerable interest in antiparasitic drug discovery. This heterocycle offers favorable physicochemical properties, including structural rigidity, electron‐withdrawing character, and the ability to act as a bioisostere for amide or ester functionalities, which can enhance metabolic stability and biological activity [15]. Notably, thiadiazole‐containing compounds have demonstrated promising activity against kinetoplastid parasites in several studies [16, 17]. Based on these considerations, we hypothesized that combining nitro‐based pharmacophores with a thiadiazole core could lead to the identification of novel compounds with enhanced antiparasitic potential.

## 2 Results and Discussion

### 2.1 Design

**Figure 2:**
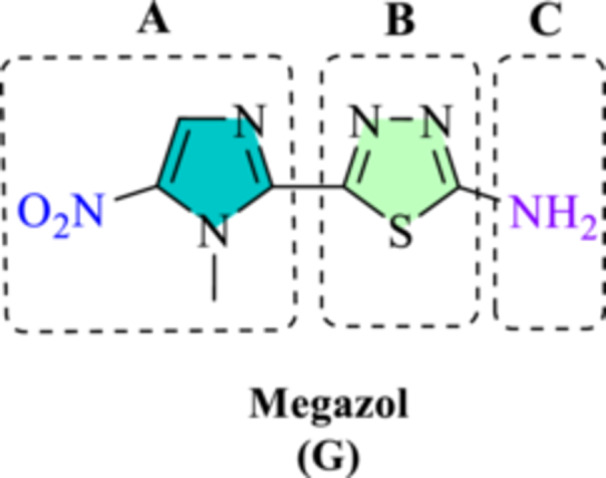
Scheme highlighting the distinct functional groups of megazol. (A) *N*‐methyl‐5‐nitroimidazole ring; (B) 1,3,4‐thiadiazole ring; (C) primary amine.

Megazol (Figure 2) is a well‐studied 5‐nitroimidazole derivative that has shown strong antiparasitic activity in preclinical evaluations. In animal models, this compound exhibited high curative efficacy against multiple strains of *T. cruzi* and *T. brucei*, and it also demonstrated potent activity against intracellular forms of *L. infantum* and *L. donovani* in vitro [10, 18]. Despite these encouraging results, the clinical application of megazol remains limited, and its efficacy and safety profile have yet to be fully established for the treatment of NDTs. Consequently, extensive efforts have focused on structural modification of this scaffold to identify new and more effective antitrypanosomal agents [10].

The chemical structure of megazol could be divided into three distinct parts: part A is *N*‐methyl‐5‐nitroimidazole ring. Removing or changing the position of nitro had a detrimental effect on the antiparasitic activity, showing its crucial role [18]. Moreover, replacing imidazole with other heterocycles like furan and thiophene resulted in compounds, showing a broad range of activity [10, 14, 19, 20, 21]. Part B is a 1,3,4‐thiadiazole ring which has been already replaced with similar moieties like oxadiazole and triazole causing a significant decrease in activity, indicating its necessary role [10, 18]. Part C is the amine functionality which has been replaced with other functional groups or aza‐heterocycles on this backbone to improve its anti‐trypanosomatid activity, as well as its pharmacokinetic and toxicological properties [10, 14, 21].

**Figure 3:**
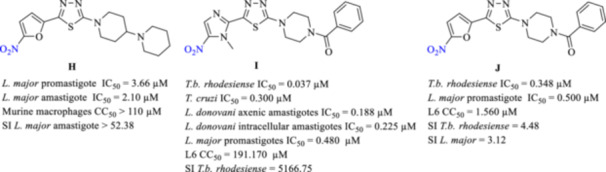
The structures of some of our previously reported nitro‐based derivatives and their anti‐trypanosomatidal activity.

Our group has previously described a series of megazol‐inspired derivatives exhibiting antitrypanosomal activity (Figure 3) [14, 20, 22, 23, 24]. Within this series, the nitrofuran‐containing analogue H showed notable inhibitory effects against both promastigote and amastigote stages of *Leishmania major* [21]. To further elucidate the contribution of the heterocyclic moiety in Part A, nitro‐substituted imidazole, furan, and thiophene rings were systematically evaluated. Among these, nitroimidazole‐based compounds, particularly analogue I, displayed superior trypanocidal potency together with the most favorable selectivity profile. Compound I achieved submicromolar IC₅₀ values against *T. b. rhodesiense* as well as the amastigote forms of *T. cruzi* and *L. donovani*, while maintaining excellent selectivity toward mammalian cells, and it produced a complete cure in an acute mouse model of HAT [14]. In contrast, the corresponding nitrofuran derivative J retained antiparasitic activity but was associated with increased cytotoxicity toward host cells. By comparison, nitrothiophene analogues were characterized by pronounced mammalian cell toxicity and lacked meaningful trypanocidal efficacy [14, 25].

**Figure 4:**
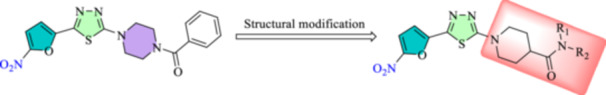
Design strategy for novel antitrypanosomal compounds **8–20**.

Building on our earlier work on antitrypanosomal agents and guided by nitrofuran‐based megazol analogues, we designed and synthesized a new series of 2‐(5‐nitro‐2‐furyl)‐1,3,4‐thiadiazole derivatives incorporating substituted 4‐piperidine carboxamide moieties **8–20** (Figure 4). These compounds were evaluated for their in vitro inhibitory activity against multiple *Trypanosoma* and *Leishmania* species. Selectivity indices were determined by assessing cytotoxicity toward the human THP‐1 cell line. In addition, in silico ADME analysis was performed to estimate the drug‐likeness of the synthesized compounds.

### 2.2 Chemistry

**Scheme 1:**
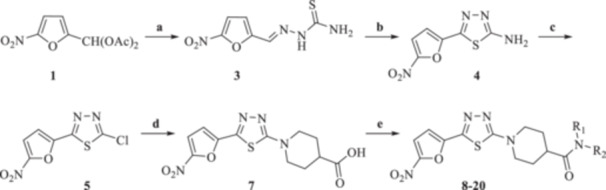
Reagents and conditions for the preparation of targeted compounds **8–20**: (a) thiosemicarbazide **2**, Conc. HCl, EtOH, reflux, 3 h; (b) NH_4_Fe(SO_4_)_2_.12H_2_O, H_2_O, reflux, 2 h; (c) NaNO_2_, HCl, Cu, H_2_O, 60°C, 20 min; (d) piperidine‐4‐carboxylic acid **6**, TEA, EtOH, reflux, 1.5 h; (e) appropriate amines, TEA, TBTU, DMF, rt, overnight.

As illustrated in Scheme 1, an efficient, multi‐step synthetic approach was performed to obtain the targeted, substituted 1‐[5‐(5‐nitrofuran‐2‐yl)‐1,3,4‐thiadiazol‐2‐yl]piperidine‐4‐carboxamides **8–20**. Initially, 5‐(5‐nitrofuran‐2‐yl)‐1,3,4‐thiadiazol‐2‐amine **4** was synthesized through a two‐step procedure. This protocol involved a nucleophilic reaction between (5‐nitrofuran‐2‐yl)methylene diacetate **1** and thiosemicarbazide **2** using concentrated hydrochloric acid (Conc. HCl) in ethanol (EtOH) under the reflux conditions within 3 h to give adduct **3**. Subsequently, this compound went through an oxidative cyclization reaction in the presence of ammonium ferric sulfate dodecahydrate in water under the reflux conditions within 2 h to obtain compound **4**.

Amine functionality was replaced with chlorine atom via diazotization reaction. To this aim, heating a mixture of compound **4**, Conc. HCl, and copper powder in water at 60°C in 20 min yielded compound **5**. Afterwards, this adduct went through the aromatic nucleophilic substitution with piperidine‐4‐carboxylic acid **6** in the presence of triethylamine (TEA) in EtOH under reflux conditions within 1.5 h, resulting into compound **7**. The amidation reaction of carboxylic acid moiety using 2‐(1*H*‐benzotriazole‐1‐yl)‐1,1,3,3‐tetramethylaminium tetrafluoroborate (TBTU) in the presence of TEA in DMF at ambient temperature overnight to afford desirable targeted products **8–20**.

**Figure 5:**
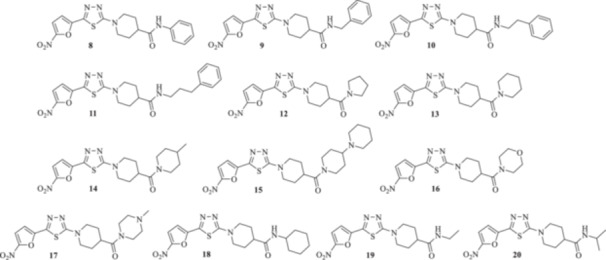
The chemical structures of targeted substituted 1‐(5‐(5‐nitrofuran‐2‐yl)‐1,3,4‐thiadiazol‐2‐yl)piperidine‐4‐carboxamides **8–20**.

To determine the generality of this procedure, various primary and secondary amines were applied to obtain the corresponding substituted 1‐[5‐(5‐nitrofuran‐2‐yl)‐1,3,4‐thiadiazol‐2‐yl]piperidine‐4‐carboxamides **8–20** presented in Figure 5. The structures of the isolated products were deduced based on their IR, ^1^H and ^13^C NMR spectroscopy, high‐resolution mass spectrometry (HRMS), and elemental analysis. Furthermore, the purity was confirmed using HPLC. Partial assignments of these resonances are given in the Experimental Part.

### 2.3 Biological Evaluations

All substituted 1‐[5‐(5‐nitrofuran‐2‐yl)‐1,3,4‐thiadiazol‐2‐yl]piperidine‐4‐carboxamides **8–20** were tested in vitro against different species of *Leishmania* and *Trypanosoma* for their antiparasitic activity. Furthermore, the cytotoxicity of all compounds **8–20** was tested against THP‐1 cells (human acute monocytic leukemia cell line) differentiated into macrophages (as summarized in Table 1) to determine the selectivity index (S.I.) for each compound against each parasite. Overall, most of the compounds presented acceptable selectivity toward this cell line. Moreover, compounds **10** and **20** exhibited no cytotoxicity against THP‐1 cells, even at 100 μM concentration. Overall, these compounds displayed better SI in comparison with our previously reported antiparasitic agents [14].

**Table 1:** Antileishmanial activities and mammalian cell cytotoxicity of compounds **8–20**.

Comp.	*L. infantum* promastigotes		*L. tropica* promastigotes		Cytotoxicity against THP1 cells
	IC_50_ μM^a^	S.I.^b^	IC_50_ μM^a^	S.I.^b^	CC_50_ μM^a^
**8**	2.09 ± 0.83	7.72	2.01 ± 0.10	8.05	16.2 ± 1.8
**9**	3.67 ± 1.71	3.88	2.51 ± 0.68	5.67	14.23 ± 4.81
**10**	3.89 ± 0.82	> 30.04	3.86 ± 1.19	> 30.29	> 100
**11**	8.96 ± 3.16	2.98	4.68 ± 3.64	5.69	27 ± 26
**12**	15.33 ± 0.21	2.60	6.78 ± 2.69	5.88	39.9 ± 17.7
**13**	7.14 ± 4.84	3.90	4.05 ± 1.01	5.97	27.82 ± 0.26
**14**	5.39 ± 1.79	6.52	6.18 ± 3.51	5.34	35.14 ± 10.08
**15**	3.23 ± 0.97	2.49	2.20 ± 1.47	2.60	8.05 ± 3.92
**16**	38.4 ± 2.6	2.00	20.48 ± 8.16	4.84	76.81 ± 18.76
**17**	21.41 ± 6.27	0.91	12.69 ± 6.01	1.12	19.50 ± 3.68
**18**	0.82 ± 0.20	29.52	0.87 ± 0.03	29.14	24.12 ± 7.74
**19**	4.89 ± 2.39	4.52	4.57 ± 1.53	4.84	22.11 ± 1.84
**20**	12.85 ± 0.21	> 10.64	12.53 ± 7.68	> 7.40	> 100
**AmB** c	0.15 ± 0.10	> 133.33	0.21 ± 0.06	> 95.24	> 20

^a^ Values are expressed as mean ± SD. All experiments were performed at least three times.

^b^ SI (selectivity index) = CC_50_ THP1/IC_50_ parasite.

^c^ AmB: Amphotericin B.

In the present study, various substituents were introduced on the amide functionality to optimize their antiparasitic potencies. To elucidate the structure and observed activity correlations, compounds **8–20** were classified into three categories based on the amine moiety: the first series bearing a phenyl ring (**8–11**), the second series bearing cyclic amine (**12–18**), and the third series bearing acyclic aliphatic amine (**19, 20**).

#### 2.3.1 Antileishmanial Activities

**Figure 6:**
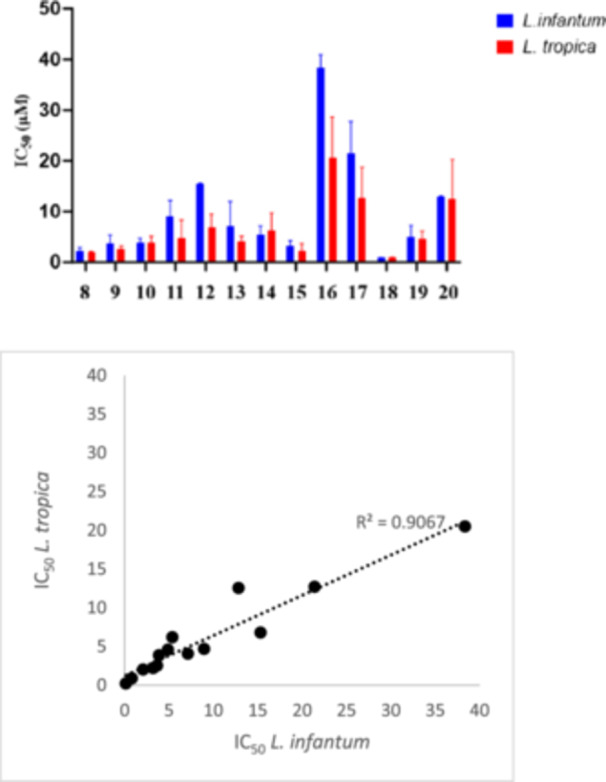
Correlation between the IC_50_ values of compounds against *L. infantum* and *L. tropica* promastigotes.

The antileishmanial activity of substituted 1‐[5‐(5‐nitrofuran‐2‐yl)‐1,3,4‐thiadiazol‐2‐yl]piperidine‐4‐carboxamides **8–20** was assessed against the promastigote forms of *L. infantum* and *L. tropica* (Table 1), as well as the intramacrophages amastigotes form of *L. infantum* (Table 2). Amphotericin B, a known medication for the treatment of leishmaniasis, was used as reference drug in these experiments. As depicted in Table 1, an overall view indicates that most compounds displayed superior inhibitory activities against *L. tropica* compared to *L. infantum* species. Moreover, Figure 6 illustrates the correlation between the IC_50_ values of compounds **8–20** against *L. infantum* and *L. tropica* promastigotes, revealing a consistent SAR trend across both species.

**Table 2:** In vitro antileishmanial activities of compounds **8–20** against intramacrophages *L. infantum* amastigotes

Compound	% inhibition at 2.5 μM^a^	IC_50_ μM^b^	S.I.^c^
**8**	60.90	1.27 ± 0.68	12.72
**9**	82.30	1.74 ± 0.38	8.17
**10**	70.19	2.44 ± 1.90	> 40.00
**11**	28.60	ND	ND
**12**	29.11	ND	ND
**13**	25.60	ND	ND
**14**	8.25	ND	ND
**15**	24.71	ND	ND
**16**	22.96	ND	ND
**17**	32.89	ND	ND
**18**	70.50	0.56 ± 0.39	42.70
**19**	46.36	ND	ND
**20**	42.14	ND	ND
**AmB**	100	0.16 ± 0.10	> 125

^a^ Mean value of two experiments.

^b^ Values are expressed as mean ± SD. All experiments were performed at least three times.

^c^ SI (selectivity index) = CC_50_ THP‐1/IC_50_ parasite.

Among the first series, compound **8** demonstrated the best inhibitory potency against both species, showing IC_50_ values of 2.09 and 2.01 μM against *L. infantum* and *L. tropica* promastigotes, respectively. Introducing a methylene spacer between the amide nitrogen and the phenyl ring resulted in a progressive decrease in antileishmanial activity, with increasing linker length directly correlating with reduced potency. This effect was more pronounced against *L. infantum*, as compound **11** exhibited approximately two‐fold higher potency against *L. tropica* promastigotes than against *L. infantum*, indicating species‐dependent sensitivity to linker elongation.

Our investigation into the second series was initiated with compound **12**, bearing five‐membered endocyclic amine, which exhibited moderate antileishmanial activities against both species. Subsequent replacement of the endocyclic amine with a six‐membered ring yielded compound **13**, which exhibited a significant improvement in activity. To further enhance the potency, several substituents were introduced at the C‐4 position of the piperidine ring. To this goal, a small group (like methyl, in compound **14**) and a bulky group (like additional piperidine, in compound **15**) were incorporated at this site. These modifications led to an enhancement in inhibition potencies, as compound **15**, having 1,4′‐bipiperidine on the amide functionality, demonstrated the best results (IC_50_ values against *L. infantum* and *L. tropica* were 3.23 μM and 2.20 μM, respectively). Considering the great potential of this position on the antileishmanial activity, subsequent modifications were explored. For instance, an oxygen atom was incorporated, leading to compound **16** bearing morpholine ring. Additionally, the introduction of an *N*‐methyl group resulted in compound **17**, featuring 1‐methylpiperazine. However, the inclusion of these moieties adversely affected the antileishmanial potencies.

Based on the promising results of compounds **13–15,** we changed the six‐membered endocyclic amine into a six‐membered exocyclic amine, resulting in compound **18**. This derivative emerged as the most potent antileishmanial agent in this study, having IC_50_ values of 0.82 μM against *L. infantum* and 0.87 μM against *L. tropica*.

In the third series, incorporation of acyclic, aliphatic amines did not result in a significant improvement in inhibitory potency, as compounds **19** and **20** displayed only moderate activity. Compared with their cyclic counterparts, the increased conformational flexibility associated with acyclic amines appears to be detrimental to antiparasitic efficacy, suggesting that a well‐defined spatial orientation and steric organization of the amide substituent are important determinants of optimal biological activity.

Following these evaluations, all substituted 1‐(5‐(5‐nitrofuran‐2‐yl)‐1,3,4‐thiadiazol‐2‐yl)piperidine‐4‐carboxamides **8–20** were examined against intramacrophage amastigotes of *L. infantum* at the single dose of 2.5 μM. Amastigote is the intracellular form of *Leishmania* parasites, which resides and replicates inside the host's cells during the infection. Subsequently, the compounds exhibiting inhibitory activities > 50% at 2.5 μM (**8**, **9**, **10**, and **18**) were chosen to determine the IC_50_ values. These results are summarized in Table 2. The activity of selected compounds against the amastigote form of *L. infantum* was higher than their potency against the promastigote form, indicating variations in sensitivity across parasite life stages. A similar trend was observed for *L. infantum* amastigotes in the current series, consistent with previous findings [21]. Compound **18** exhibited the most potent inhibitory activity.

Considering the results presented in Tables 1 and 2, compound **18** emerged as the most potent derivative having remarkable IC_50_ values of 0.82 μM, 0.87 μM, and 0.56 μM against *L. infantum* promastigotes, *L. tropica* promastigotes, and *L. infantum* amastigotes, respectively. Therefore, this compound can be considered a promising candidate for further examinations as antileishmanial agents.

#### 2.3.2 Antitrypanosomal Activities

This study progressed by evaluating the antitrypanosomal activities of substituted 1‐[5‐(5‐nitrofuran‐2‐yl)‐1,3,4‐thiadiazol‐2‐yl]piperidine‐4‐carboxamides **8–20** against the bloodstream form of different *Trypanosoma* species including *T. brucei gambiense*, *T. brucei brucei*, and *T. congolense*. Hygromycin B (HygB) is a known trypanocidal agent used as a reference drug in this examination. The observed results are presented in Table 3.

**Table 3:** In vitro antitrypanosomal activities of compounds **8–20**.

Compound	*T. b. gambiense*		*T. b. brucei*		*T. congolense*	
	IC_50_ μM^a^	S.I.^b^	IC_50_ μM^a^	S.I.^b^	IC_50_ μM^a^	S.I.^b^
**8**	0.44 ± 0.06	36.73	0.41 ± 0.11	39.41	0.21 ± 0.13	76.95
**9**	1.09 ± 0.04	13.05	1.36 ± 0.14	10.46	0.24 ± 0.18	59.29
**10**	0.22 ± 0.20	> 454.54	0.38 ± 0.04	> 263.16	0.19 ± 0.13	> 526.31
**11**	0.35 ± 0.01	76.17	0.39 ± 0.02	68.36	0.19 ± 0.11	140.31
**12**	3.78 ± 0.16	10.55	4.49 ± 0.17	8.89	0.83 ± 0.69	48.07
**13**	1.84 ± 0.29	15.12	2.23 ± 0.81	12.47	1.32 ± 0.72	21.07
**14**	0.82 ± 0.05	42.85	0.89 ± 0.19	39.48	0.51 ± 0.36	68.90
**15**	0.13 ± 0.03	61.92	0.17 ± 0.01	47.35	0.16 ± 0.03	50.31
**16**	6.67 ± 0.32	11.51	6.17 ± 0.57	12.45	0.93 ± 0.75	82.59
**17**	2.87 ± 0.88	6.79	5.6 ± 1.0	3.48	0.96 ± 0.56	20.31
**18**	0.23 ± 0.04	104.87	0.31 ± 0.10	77.81	0.22 ± 0.16	109.64
**19**	2.20 ± 0.39	10.05	3.0 ± 0.6	7.47	0.34 ± 0.22	65.03
**20**	13.5 ± 2.5	> 7.40	5.23 ± 2.23	> 19.12	0.39 ± 0.14	> 256.41
**HygB** ^**c**^	0.63 ± 0.31	ND	0.72 ± 0.01	ND	0.08 ± 0.02	ND

^a^ Values are expressed as mean ± SD. All experiments were performed at least two times.

^b^ SI (selectivity index) = CC_50_ THP‐1/IC_50_ parasite.

*T. b. gambiense* causes a slowly progressing form of chronic trypanosomiasis in humans, predominantly prevalent in West and Central Africa [26]. *T. b. brucei* serves as a widely utilized research model for parasitic studies considering its non‐pathogenicity to humans, while retaining all other characteristics of the pathogenic species [27]. *T. congolense* is the major pathogen causing nagana disease, which is found in cattle and other animals, thereby exerting a detrimental impact on Africa's livestock industry [28].

**Figure 7:**
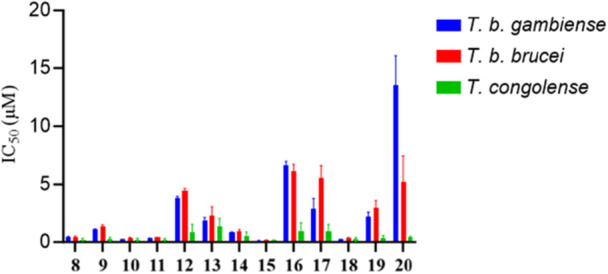
Correlation between the IC_50_ values of compounds **8–20** against *T. b. gambiense*, *T. b. brucei*, and *T. congolense* bloodstreams.

Figure 7 presents an overall comparison of compounds **8–20** against *T. b. gambiense*, *T. b. brucei*, and *T. congolense*. Moreover, Table 3 shows that most compounds manifest a similar trend in the IC_50_ values against *T. b. gambiense* and *T. b. brucei* species. Furthermore, most compounds exhibited the superior inhibitory potencies against *T. congolense* when compared to other species. This trend was also observed in previously reported nitro‐based antitrypanosomal agents [29].

Analysis of structure–activity relationships against *T. b. gambiense* and *T. b. brucei* revealed distinct trends across the compound series. Within the first series, in contrast to the antileishmanial results, elongation of the linker between the amide nitrogen and the terminal phenyl ring led to enhanced trypanocidal potency. Among these analogues, compound **10** showed the highest activity within this subset.

For the second series, SAR trends were generally consistent with those observed for antileishmanial activity against promastigote forms. Introduction of a pyrrolidine ring resulted in a partial loss of potency, as evidenced by compound **12**, which exhibited an approximately 10‐fold reduction in activity against both *T. b. gambiense* and *T. b. brucei*. Expansion to six‐membered rings restored activity, with compounds **13** and **14** bearing piperidine and 4‐methylpiperidine moieties displaying improved trypanocidal effects. Further optimization led to compound **15**, which emerged as the most potent antitrypanosomal agent in this study, exhibiting IC₅₀ values of 0.13 ± 0.03 µM, 0.17 ± 0.01 µM, and 0.16 ± 0.03 µM against *T. b. gambiense*, *T. b. brucei*, and *T. congolense*, respectively. Increasing the polarity of the piperidine scaffold by incorporation of morpholine or *N*‐methylpiperazine substituents attenuated activity against *T. b. gambiense* and *T. b. brucei*. In line with the leishmanicidal SAR, introduction of a cyclohexylamine moiety yielded compound **18**, which showed strong antitrypanosomal potency and outperformed the reference drug against both *T. b. gambiense* and *T. b. brucei*. In contrast, increasing molecular flexibility through linear aliphatic substituents (**19** and **20**) resulted in a pronounced reduction in activity, with **20** displaying the weakest potency against *T. b. gambiense* (IC₅₀ = 13.5 µM).

Activity trends against *T. congolense* differed notably from those observed for *T. b. gambiense* and *T. b. brucei*. With the exception of **13**, all compounds exhibited submicromolar IC₅₀ values against this species. In the first series, variation in linker length between the phenyl group and the amide moiety had little influence on activity. In contrast to the other species, morpholine‐ and N‐methylpiperazine‐containing derivatives showed improved potency relative to piperidine analogues. Compound **18** remained highly active, displaying comparable IC₅₀ values across all three *Trypanosoma* species. Notably, compounds **19** and **20** bearing linear aliphatic amines retained strong activity against *T. congolense*, with IC₅₀ values of 0.34 and 0.39 µM, respectively.

Overall, these SAR observations are consistent with previous reports on nitro‐containing antitrypanosomal agents [30, 31, 32, 33, 34]. Earlier studies have demonstrated that substitution at the amide position strongly influences trypanocidal potency and selectivity toward mammalian cells [30]. Increased molecular flexibility and the nature of terminal substituents, including electronic effects and lipophilicity, have been shown to modulate activity across *Trypanosoma* species [31, 32, 33, 34, 35]. In agreement with these findings, the present study confirms that careful optimization of the amide‐linked substituent and overall physicochemical properties is critical for achieving potent and selective antitrypanosomal activity.

**Figure 8:**
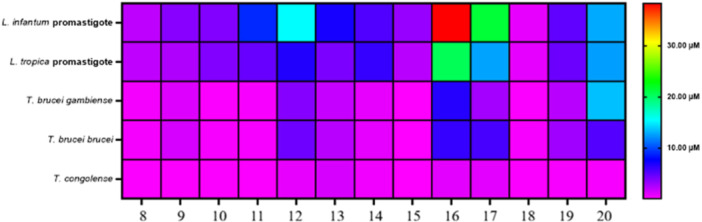
Anti‐kinetoplastid activity heat map of compounds **8–20**.

An activity heatmap was prepared to illustrate the anti‐trypanosomatid activities of substituted 1‐[5‐(5‐nitrofuran‐2‐yl)‐1,3,4‐thiadiazol‐2‐yl]piperidine‐4‐carboxamides **8–20** against *L. infantum* and *L. tropica* promastigotes and *T. b. gambiense*, *T. b. brucei*, and *T. congolense* bloodstreams (Figure 8). The activity level of compounds is represented by color‐coding based on their IC_50_ values: purple for high inhibition, green for moderate inhibition, and red for low inhibition. Overall, our SAR analysis revealed that all compounds **8‐20** exhibited superior potencies against *Trypanosoma* species compared to *Leishmania* species. Moreover, the best results were observed against *T. congolense* parasites. Notably, compound **18** possessed the best in vitro antileishmanial and antitrypanosomal activities, as evidenced by their impressive profiles shown in Figure 8.

#### 2.3.3 Evaluation of cellular and mitochondrial ROS levels in *T. b. brucei* bloodstream cells treated with selected compounds

**Figure 9:**
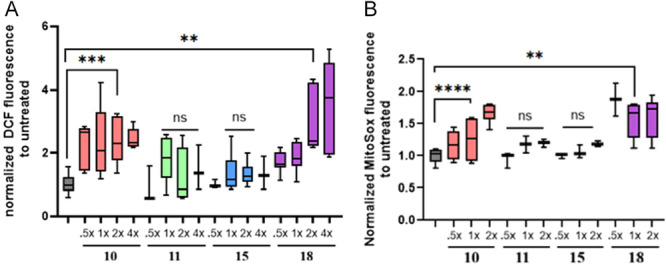
Left graph (A): Assessment of cellular ROS species in *T. b. brucei* BF cells treated with compounds **10**, **11**, **15** and **18**. Right graph (B): Assessment of mitochondrial ROS species in *T. b. brucei* BF cells treated with compounds **10**, **11**, **15** and **18**.

To further elucidate the potential mechanism of action of the selected compounds, the generation of reactive oxygen species (ROS) was assessed in *T. b. brucei* bloodstream form cells. The evaluation included both intracellular and mitochondrial ROS measurements using flow cytometry, following treatment with four selected compounds (**10**, **11**, **15**, and **18**) at concentrations ranging from 0.5× to 4× their respective EC_50_ values. Notably, compounds **10** and **18** induced a marked increase in both intracellular and mitochondrial ROS levels, while **11** and **15** did not show any significant elevation in ROS production (Figure 9).

Despite the observed ROS generation for **10** and **18**, the similarity in EC_50_ values across all four compounds suggests that ROS induction is unlikely to play a central role in their antiparasitic activity. This observation implies that the inhibitory activity of these derivatives might be attributed to alternative mechanisms independent of oxidative stress.

#### 2.3.4 Apoptosis Assessment and Cytostatic Effects of Selected Compounds on *T. b. brucei* Bloodstream Form

**Figure 10:**
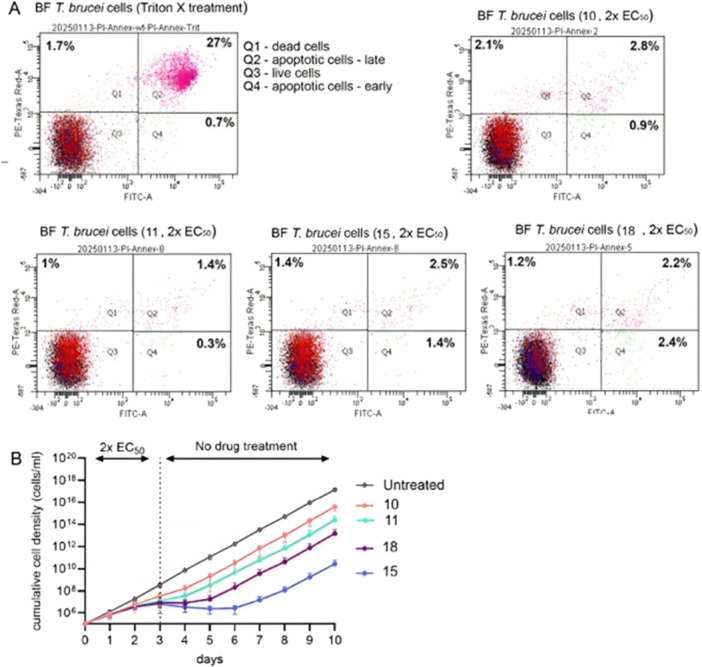
Treatment with **10**, **11**, **15**, and **18** induces a reversible cytostatic effect in *Trypanosoma brucei* cultures. (A) Representative scatter plots from flow cytometry‐based live/dead staining of *T. b. brucei* cells following treatment with selected compounds. (B) Growth curve analysis of *T. b. brucei* cells treated with selected compounds for 3 days, followed by centrifugation and resuspension in fresh medium for an additional 7 days to assess recovery.

To determine whether the examined compounds exert a cytostatic or cytotoxic effect on the *T. b. brucei* cell population, we employed the Dead Cell Apoptosis Kit with Annexin V conjugates and propidium iodide (PI) to differentiate between live, apoptotic, and dead cells. Treatment of the *T. b. brucei* bloodstream form cells with Triton X‐100 (0.2%) resulted in cell death, as indicated by a subpopulation displaying high green fluorescence (indicative of apoptosis) and high red fluorescence (indicative of increased cell membrane permeability, a hallmark of cell death) (Figure 10A). Interestingly, treatment with four selected compounds (**10**, **11**, **15**, and **18**) did not induce either apoptosis or cell death (Figure 10A). Only a small fraction of the cell population (approximately 2%) exhibited high red and green fluorescence signals. These findings suggest that compounds induce a cytostatic rather than a cytotoxic effect. To assess whether this effect was reversible, we cultured the cells for 3 days under the 2× EC_50_ compound exposure. Subsequently, the cells were spun down and resuspended in fresh medium without the compounds (Figure 10B). For cells treated with **10** and **11**, growth recovery was almost immediate. In contrast, cells treated with **15** and **18** required 48–72 h to fully recover.

Considering that the resazurin‐based Alamar Blue assay relies on the reduction of resazurin to fluorescent resorufin by NADH, NADPH, FADH, or other biologically abundant reducing agents in the presence of mitochondrial and cytosolic reductases, the overall reductive power of the cell can serve as a measure of viability. However, it can also indicate metabolic arrest and a transition to a quiescent state.

Taken together, our data suggest that the examined compounds effectively induce metabolic arrest, as evidenced by the resazurin‐based assay, and reduce proliferation, as indicated by growth curve analysis, without directly inducing cell death, as assessed by the cell death assay.

### 2.4 Molecular Docking Studies

To gain supportive insight into the molecular interactions that may contribute to the antitrypanosomal activity of the present series, molecular docking studies were conducted for the two most potent compounds, **10** and **18**. As pteridine reductase 1 (PTR1) and dihydrofolate reductase–thymidylate synthase (DHFR‐TS) are among the most established molecular targets for antikinetoplastid agents, representative *Trypanosoma* protein structures associated with these enzymes and having available crystallographic data were selected for this exploratory analysis (PDB IDs: 3CLB, 3GN2, and 3RG9) [36, 37, 38, 39, 40].

The docking protocol was first validated by re‐docking the co‐crystallized ligands into their respective binding sites, confirming reasonable reproduction of the experimental binding modes. Compounds **10** and **18** were subsequently docked into the same binding pockets to enable qualitative analysis of their predicted interaction profiles. The predicted binding energies (Δ*G*) obtained from the docking simulations are summarized in Table 4.

**Table 4:** Docking results of compounds 10 and 18 against selected *Trypanosoma* protein structures

Compound	PDB code	Lowest binding energy (kcal/mol)	Residues involved in hydrogen bonding
**10**	3CLB	−13.09	ARG94, ILE154
**18**	3CLB	−12.30	ILE41, ARG94
**10**	70.19	−10.62	LEU208
**18**	28.60	−12.31	ILE15, LEU208
**10**	29.11	−13.38	ARG100, ILE160
**18**	100	−13.89	THR86, SER89, ARG100

**Figure 11:**
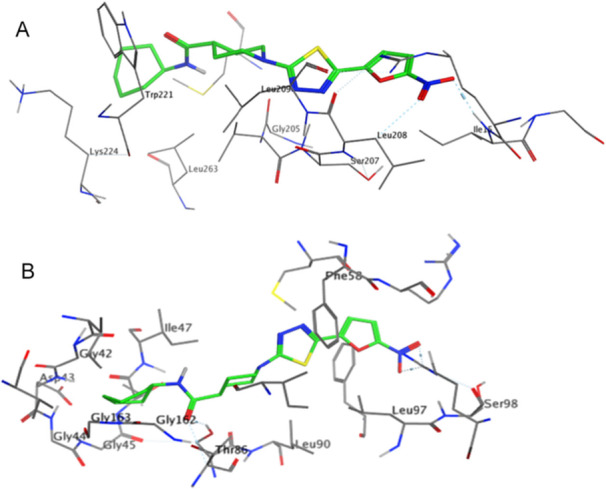
Predicted binding modes of compound **18**.

Both compounds adopted stable binding orientations within the active‐site cavities of all three protein structures. Inspection of the predicted binding poses revealed that the nitroheterocyclic core plays a central role in ligand anchoring, frequently engaging in one or two hydrogen‐bond interactions with polar residues within the binding pocket. Additional contacts were observed between the amide‐linked substituents and surrounding residues, contributing to the overall interaction patterns. Representative binding modes of compound **18** within the active sites of 3GN2 and 3RG9 are illustrated in Figure 11A,B, highlighting key amino acid residues involved in ligand recognition.

Notably, the predicted binding modes of compounds **10** and **18** differed from those of the respective co‐crystallized ligands, which is consistent with the distinct chemical scaffolds of the test compounds compared to the native ligands. These differences were also reflected in the involvement of alternative active‐site residues in hydrogen‐bonding interactions, particularly those engaging the nitroheterocyclic moiety. Such variations are expected in exploratory docking studies of chemically unrelated ligands and further emphasize the qualitative, hypothesis‐generating nature of the present docking analysis rather than definitive target validation.

Accordingly, the docking results are intended to provide supportive qualitative insight into possible ligand–protein interactions rather than to quantitatively explain differences in biological potency. Given the inherent limitations of rigid docking approaches and the absence of direct biochemical validation, the predicted binding energies should be interpreted cautiously. Nevertheless, the observed interaction patterns are consistent with the experimental SAR and support the relevance of the nitroheterocyclic scaffold and amide‐linked substituents for antitrypanosomal activity.

### 2.5 In Silico Analysis of Physicochemical Parameters

In the field of medicinal chemistry, using computational tools has become an indispensable part of the early stages of drug discovery and development to elucidate the pharmacokinetic profile of the substituted 1‐[5‐(5‐nitrofuran‐2‐yl)‐1,3,4‐thiadiazol‐2‐yl]piperidine‐4‐carboxamides **8–20**, we utilized the SwissADME web‐based tool (Table 5) [41]. This pivotal step allowed us to explore the molecular properties which are crucial for evaluating the drug‐likeness according to Lipinski's Rule of Five. All compounds have successfully met Lipinski's Rule of Five criteria, indicating their strong potential for drug‐likeness. The Polar Surface Area (PSA) is a vital molecular descriptor that influences a drug's ability to permeate cell membranes and impact biological activity. Among 13 compounds, 8 derivatives possess PSA values slightly above the optimal threshold at around 145, while 5 remainders have PSA values below 140, indicating closer alignment with the recommended optimum. The Number of Rotatable Bonds (NRB) offers insights into the compound's flexibility and conformational freedom, which impact bioavailability and metabolic stability. All compounds exhibited approved NRB amounts of less than 10.

**Table 5:** Swiss ADME pharmacokinetics prediction for the compounds **8–20**.

Compound	MW	clogP	H‐bond donors	H‐bond Acceptors	PSA (Å2)	NRB
**8**	399.42	2.45	1	6	145.32	6
**9**	413.45	2.52	1	6	145.32	7
**10**	443.52	2.50	1	6	145.32	8
**11**	441.50	3.12	1	6	145.32	9
**12**	377.42	1.89	0	6	136.53	5
**13**	391.44	2.18	0	6	136.53	5
**14**	405.47	2.35	0	6	145.76	5
**15**	474.58	2.55	0	7	136.53	5
**16**	393.42	1.31	0	7	145.76	5
**17**	406.46	1.25	0	7	139.77	5
**18**	405.47	2.59	1	6	145.32	6
**19**	351.38	1.58	1	6	145.32	6
**20**	365.41	1.88	1	6	145.32	6
**OPd**	< 500	< 5	≤ 5	≤ 10	≤ 140	≤ 10

Abbreviations: MW, molecular weight; NRB, number of rotatable bonds; OP, optimal properties for bioavailability and oral absorption; PSA, polar surface area.

**Figure 12:**
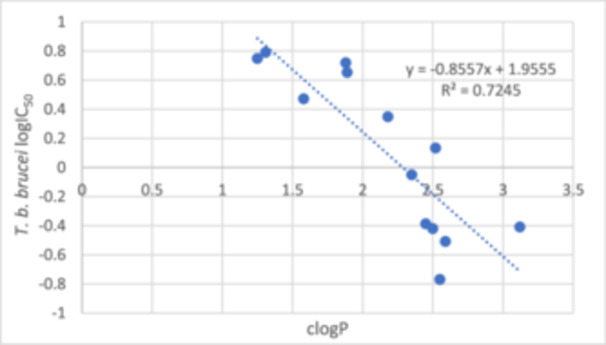
Correlation between lipophilicity (clogP values) and activity against *T. b. brucei* (logIC_50_ values) of compounds **8–20**.

To explore the impact of lipophilicity on the antiparasitic efficacy of the substituted 1‐[5‐(5‐nitrofuran‐2‐yl)‐1,3,4‐thiadiazol‐2‐yl]piperidine‐4‐carboxamides **8–20**, correlation plot was outlined (Figure 12), illustrating the relationship between logIC_50_ values for each parasite and clogP values. Apart from *T. b. brucei*, which exhibited an *R* ^2^ value of 0.72, all other parasites demonstrated *R* ^2^ values below 0.60. This indicates a limited correlation between antiparasitic activity and the lipophilicity of the compounds **8–20**.

## 3 Conclusions

In conclusion, this study highlights the continued need for novel antiparasitic agents and underscores the potential of nitrofuran‐based compounds in the treatment of neglected tropical diseases. We designed and synthesized a series of substituted 1‐[5‐(5‐nitrofuran‐2‐yl)‐1,3,4‐thiadiazol‐2‐yl]piperidine‐4‐carboxamides (**8–20**) using a straightforward, chromatography‐free synthetic route from readily available materials. These compounds were evaluated in vitro against multiple kinetoplastid parasites, including *L. infantum*, *L. tropica*, *T. b. gambiense*, *T. b. brucei*, and *T. congolense*, with a focus on both efficacy and selectivity.

Among the tested derivatives, compound **18** emerged as the most potent antileishmanial and antitrypanosomal agent, surpassing even Hygromycin B against *T. b. gambiense* and *T. b. brucei*. Additionally, compound **10** demonstrated strong inhibitory activity with an exceptional selectivity profile. Mechanistic studies revealed that while **10** and **18** induced elevated ROS levels in *T. b. brucei*, they did not trigger apoptosis. Instead, these compounds caused a reversible cytostatic effect, likely by inducing metabolic arrest.

These findings not only validate the therapeutic potential of compounds **18** and **10** but also offer mechanistic insight into their antiparasitic action. Further studies exploring their molecular targets, *in vivo* efficacy, and comprehensive toxicity are warranted to support their progression toward preclinical development.

## 4 Experimental

### 4.1 Chemistry

#### 4.1.1 General remarks

All starting materials, reagents, and solvents were purchased from Merck and Aldrich companies without any purification. The reaction progress and the purity of synthesized compounds were monitored by thin‐layer chromatography (TLC) on silica gel 250‐μm F254 plastic sheets; zones were detected visually under UV light (254 nm). The melting points were determined by the Electrothermal IA9100 apparatus. IR spectra were obtained on PerkinElmer Spectrum Version 10.03.06 (potassium bromide disks). ^1^H and ^13^C NMR spectra were measured (CDCl_3_ or DMSO‐*d*_6_ solution) with Bruker DRX‐300 (at 300.1 and 75.5 MHz) and Bruker DRX‐500 AVANCE (at 500.1 and 125.8 MHz) instruments. Chemical shifts were reported in parts per million (ppm), downfield from tetramethylsilane. Proton coupling patterns were described as singlet (s), doublet (d), triplet (t), multiplet (m), and broad (br.). Elemental analyses for C, H, and N were performed using a Heraeus CHN‐O‐Rapid analyzer. HRMS analysis was performed using a Waters Synapt G1 HDMS High Definition mass spectrometer equipped with an electrospray ionization (ESI) source. The samples were prepared by diluting the isolated compounds in methanol to a final concentration of 10 µg/mL. The analysis was conducted in positive ion mode with a mass range of *m/z* 100–1000. The chromatographic purity of the compounds was determined using HPLC‐UV and was always at least 95%; chromatograms are available from the supplementary information.

#### 4.1.2 Synthesis of 1‐[5‐(5‐Nitrofuran‐2‐yl)‐1,3,4‐Thiadiazol‐2‐yl]piperidine‐4‐Carboxylic Acid (3)

To the stirring solution of (5‐nitrofuran‐2‐yl)methylene diacetate **1** (8.68 g, 35.73 mmol) and thiosemicarbazide **2** (3.25 g, 35.73 mmol) in EtOH (50 mL) under the reflux conditions, Conc. HCl (1.8 mL) was added dropwise, and the resultant mixture was heated within 3 h. Upon completion of the reaction (confirmed by TLC), the mixture was allowed to cool to room temperature. An orange solid precipitate was subsequently filtered and washed with cold EtOH to give the desirable compound **3** (7.26 g, 33.94 mmol, 95%).

#### 4.1.3 Synthesis of 5‐(5‐Nitrofuran‐2‐yl)‐1,3,4‐Thiadiazol‐2‐Amine (4)

Compound **3** (7.26 g, 33.94 mmol) was added to NH_4_Fe(SO_4_)_2_.12H_2_O (16.36 g, 33.94 mmol) along with water (88 mL), then refluxed for 1 h. Afterwards, an additional amount of iron salt (34.52 g, 71.61 mmol) and water (149.31 mL) were added, and heating continued for a further 1 h. After completion of the reaction and cooling to room temperature, the resulting precipitate was filtered, washed with cold water, and recrystallized with DMF to yield the pure product **4** as a yellow solid (4.60 g, 21.72 mmol, 64%).

#### 4.1.4 Synthesis of 2‐Chloro‐5‐(5‐Nitrofuran‐2‐yl)‐1,3,4‐Thiadiazole (5)

Compound **4** (4.60 g, 21.72 mmol) was ground with an excess amount of NaNO_2_ (4.77 g, 69.10 mmol) to be added under the ice bath conditions to a mixture of Conc. HCl (64.5 mL) and Cu powder (1.15 g, 17.97 mmol) in water (27.64 mL) within 30 min. Afterwards, the stirring continued for 20 min at 60°C. Upon completion of the reaction, which was monitored by TLC, the mixture was cooled and extracted three times with CHCl_3_ (3 × 100 mL). The combined organic extracts were washed with brine, dried over Na_2_SO_4_, and then concentrated. The orange precipitate was filtered and washed with Et_2_O to afford pure product **5** (4.11 g, 17.81 mmol, 82%) [25].

#### 4.1.5 Synthesis of 1‐(5‐(5‐Nitrofuran‐2‐yl)‐1,3,4‐Thiadiazol‐2‐yl)Piperidine‐4‐Carboxylic Acid (7)

To a stirring solution containing compound **5** (2.31 g, 10.0 mmol) and piperidine‐4‐carboxylic acid **6** (1.42 g, 11.0 mmol), Et_3_N (1.52 g, 15.0 mmol) was added and refluxed for 1.5 h. As the reaction was completed, the mixture was put in the refrigerator for the appropriate time to precipitate. The solid residue was filtered and washed with EtOH to give the pure product **7** as a pale orange powder (4.21 g, 13.0 mmol, 73%). Orange solid, mp 278°C–279°C. IR (KBr) (*ν*_max_/cm^–1^): 3350–3100 (br., OH), 1718 (CO), 1548, 1509, 1417, 1367, 1308, 1298, 1207, 1143, 1054, 972, 848, 755, 740, 623. ^1^H NMR (500.1 MHz, DMSO‐*d*_6_): *δ* 12.38 (s, 1H, CO_2_H), 7.84 (d, *J* = 4.0 Hz, 1H, CH‐furan), 7.35 (d, *J* = 4.0 Hz, 1H, CH‐furan), 3.94–3.87 (m, 2H, 2CHN‐piperidine), 3.32–3.20 (m, 2H, 2CHN‐piperidine), 3.39–3.31 (m, 2H, CH_2_NH), 2.62–2.53 (m, 1H, CH‐piperidine), 2.02–1.92 (m, 2H, CH_2_‐piperidine), 1.71–1.61 (m, 2H, CH_2_‐piperidine). ^13^C NMR (125.1 MHz, DMSO‐*d*_6_) *δ* 173.81, 172.80, 151.92, 147.89, 145.50, 142.19, 128.76, 128.73, 126.18, 115.62, 112.34, 50.10, 42.86, 41.37, 32.97, 31.40, 28.03. Anal. Calcd. for C_12_H_12_N_4_O_5_S: C, 44.44; H, 3.73; N, 17.28; found: C, 44.58; H, 3.98; N, 17.42%. HRMS (ESI) *m/z* for C_12_H_13_N_4_O_5_S^+^ [M + H]^+^, calculated: 325.0601, found: 325.0608.

#### 4.1.6 General Procedure for the Synthesis of Substituted 1‐[5‐(5‐Nitrofuran‐2‐yl)‐1,3,4‐Thiadiazol‐2‐yl]Piperidine‐4‐Carboxamides (**8–20**)

A mixture of compound **7** (0.324 g, 1.0 mmol) and TBTU (0.493 g, 1.3 mmol) in DMF (5.0 mL) was stirred at ambient temperature for 45 min. Simultaneously, in another container, the corresponding amine (1.3 mmol) and Et_3_N (0.202 g, 2.0 mmol) in DMF (5.0 mL) were stirred under the same conditions. Afterwards, the mixture in the second container was added dropwise to the stirring solution in the first container, and the reaction continued stirring overnight to complete. Then, the reaction mixture was quenched with water to solidify. The resulting precipitate was isolated by filtration, washed with the appropriate amount of water, and dried under vacuum. The obtained residue was finally purified by recrystallization in EtOH, yielding the targeted compounds **8–20** in moderate to great yields ranging from 54% to 76%.

1‐[5‐(5‐Nitrofuran‐2‐yl)‐1,3,4‐thiadiazol‐2‐yl]‐*N*‐phenylpiperidine‐4‐carboxamide (**8**): Yellow solid, mp 254°C–256°C. IR (KBr) (*ν*_max_/cm^–1^): 3169 (NH), 1640 (CO), 1521, 1509, 1492, 1457, 1427, 1383, 1369, 1351, 1296, 1254, 1241, 1183, 1158, 1097, 1010, 988, 934, 808. ^1^H NMR (300.1 MHz, CDCl_3_): *δ* 9.61 (s, 1H, NH‐amide), 7.52 (d, *J* = 7.8 Hz, 2H, 2CH‐Ph), 7.43 (d, *J* = 3.8 Hz, 1H, CH‐furan), 7.40–7.25 (m, 3H, 3CH‐Ph), 7.16 (d, *J* = 3.8 Hz, 1H, CH‐furan), 4.22–4.08 (m, 2H, 2CHN‐piperidine), 3.46–3.20 (m, 2H, 2CHN‐piperidine), 2.65–2.45 (m, 1H, CH‐piperidine), 2.17–1.94 (m, 4H, 2CH_2_‐piperidine). ^13^C NMR (125.1 MHz, DMSO‐d6) δ 172.96 (C), 172.77 (C), 151.92 (CO), 147.86 (C), 145.54 (C), 139.66 (C), 129.12 (2×CH), 123.59 (CH), 119.61 (2 × CH), 115.60 (CH), 112.36 (CH), 49.99 (2 × CH2N), 49.84 (2 × CH2N), 42.26 (CH), 27.89 (CH2), 27.39 (CH2). Anal. Calcd. for C_18_H_17_N_5_O_4_S: C, 54.13; H, 4.29; N, 17.53; found: C, 53.96; H, 4.44; N, 17.27%. HRMS (ESI) *m/z* for C_18_H_18_N_5_O_4_S^+^ [M + H]^+^, calculated: 400.1074, found: 400.1086. HPLC purity: 99.93% (Rt = 24.78 min).

*N*‐Benzyl‐1‐[5‐(5‐nitrofuran‐2‐yl)‐1,3,4‐thiadiazol‐2‐yl]piperidine‐4‐carboxamide (**9**): Orange solid, mp 271°C–273°C. IR (KBr) (*ν*_max_/cm^–1^): 3195 (NH), 1668 (CO), 1585, 1511, 1447, 1412, 1398, 1356, 1312, 1302, 1288, 1246, 1178, 1139, 1085, 1014, 991, 940, 877, 823. ^1^H NMR (300.1 MHz, CDCl_3_): *δ* 7.43 (d, *J* = 3.9 Hz, 1H, CH‐furan), 7.40–7.23 (m, 5H, 5CH‐Ph), 7.15 (d, *J* = 3.9 Hz, 1H, CH‐furan), 5.91 (t, *J* = 5.6 Hz, 1H, NH‐amide), 4.46 (d, *J* = 5.6 Hz, 2H, CH_2_‐Ph), 4.16–4.05 (m, 2H, 2CHN‐piperidine), 3.38–3.23 (m, 2H, 2CHN‐piperidine), 2.57–2.37 (m, 1H, CH‐piperidine), 2.08–1.86 (m, 4H, 2CH_2_‐piperidine). ^13^C NMR (75.5 MHz, CDCl3): δ 173.20 (C), 172.73 (C), 150.09 (CO), 148.34 (C), 145.83 (C), 137.92 (C), 128.81 (2 × CH), 127.77 (2 × CH), 127.70 (CH), 113.81 (CH), 110.39 (CH), 49.79 (2 × CH2N), 43.65 (CH2NH), 42.28 (CH), 27.84 (2 × CH2). Anal. Calcd. for C_19_H_19_N_5_O_4_S: C, 55.19; H, 4.63; N, 16.94; found: C, 55.38; H, 4.37; N, 16.82%. HRMS (ESI) *m/z* for C_19_H_20_N_5_O_4_S^+^ [M + H]^+^, calculated: 414.1231, found: 414.1248. HPLC purity: 99.74% (Rt = 24.27 min).

1‐[5‐(5‐Nitrofuran‐2‐yl)‐1,3,4‐thiadiazol‐2‐yl]‐*N*‐phenethylpiperidine‐4‐carboxamide (**10**): Orange solid, mp 289°C–293°C. IR (KBr) (*ν*_max_/cm^–1^): 3215 (NH), 1651 (CO), 1539, 1515, 1493, 1454, 1371, 1358, 1325, 1310, 1274, 1250, 1226, 1209, 1193, 1100, 1030, 1019, 1004, 992, 943, 894. ^1^H NMR (500.1 MHz, CDCl_3_): *δ* 7.45 (d, *J* = 3.9 Hz, 1H, CH‐furan), 7.35 (t, *J* = 7.4 Hz, 2H, 2CH‐Ph), 7.26 (t, *J* = 7.6 Hz, 1H, CH‐Ph), 7.21 (d, *J* = 7.3 Hz, 2H, 2CH‐Ph), 7.18 (d, *J* = 3.9 Hz, 1H, CH‐furan), 5.50 (t, *J* = 5.7 Hz, 1H, NH‐amide), 4.15–4.03 (m, 2H, 2CHN‐piperidine), 3.65–3.53 (m, 2H, CH_2_NH), 3.40–3.20 (m, 2H, 2CHN‐piperidine), 2.87 (t, *J* = 6.8 Hz, 2H, CH_2_‐Ph), 2.40–2.25 (m, 1H, CH‐piperidine), 2.05–1.75 (m, 4H, 2CH_2_‐piperidine). ^13^C NMR (125.1 MHz, DMSO‐d6) δ 173.75 (C), 172.77 (C), 151.92 (CO), 147.87 (C), 145.48 (C), 139.90 (C), 129.11 (2 × CH), 128.71 (2 × CH), 126.49 (CH), 115.60 (CH), 112.33 (CH), 50.03 (2 × CH2N), 42.52 (CH), 41.25 (NCH2CH2), 35.55 (NCH2CH2), 27.93 (2 × CH2). Anal. Calcd. for C_20_H_21_N_5_O_4_S: C, 56.19; H, 4.95; N, 16.38; found: C, 56.42; H, 5.15; N, 16.66%. HRMS (ESI) *m/z* for C_20_H_22_N_5_O_4_S^+^ [M + H]^+^, calculated: 428.1387, found: 428.1372. HPLC purity: 99.71% (Rt = 24.42 min).

1‐[5‐(5‐Nitrofuran‐2‐yl)‐1,3,4‐thiadiazol‐2‐yl]‐*N*‐(3‐phenylpropyl)piperidine‐4‐carboxamide (**11**): Orange solid, mp 305°C–308°C. IR (KBr) (*ν*_max_/cm^–1^): 3289 (NH), 1668 (CO), 1568, 1563, 1524, 1501, 1498, 1437, 1415, 1378, 1343, 1302, 1295, 1232, 1178, 1144, 1085, 1041, 992, 936, 829. ^1^H NMR (500.1 MHz, DMSO‐*d*_6_): *δ* 7.90 (t, *J* = 5.0 Hz, 1H, NH‐amide), 7.86 (d, *J* = 4.0 Hz, 1H, CH‐furan), 7.36 (d, *J* = 4.0 Hz, 1H, CH‐furan), 7.27 (t, *J* = 7.4 Hz, 2H, 2CH‐Ph), 7.24–7.10 (m, 3H, 3CH‐Ph), 4.08–3.90 (m, 2H, 2CHN‐piperidine), 3.32–3.20 (m, 2H, 2CHN‐piperidine), 3.12–3.00 (m, 2H, CH_2_NH), 2.56 (t, *J* = 7.6 Hz, 2H, CH_2_‐Ph), 2.48–2.35 (m, 1H, CH‐piperidine), 1.88–1.79 (m, 2H, CH_2_CH_2_CH_2_), 1.75–1.60 (m, 4H, 2CH_2_‐piperidine). ^13^C NMR (125.1 MHz, DMSO‐d6) δ 173.81 (C), 172.80 (C), 151.92 (CO), 147.89 (C), 145.50 (C), 142.19 (C), 128.76 (2 × CH), 128.73 (2 × CH), 126.18 (CH), 115.62 (CH), 112.34 (CH), 50.10 (2 × CH_2_N), 42.86 (CH), 41.37 (NCH_2_CH_2_CH_2_), 32.97 (NCH_2_CH_2_CH_2_), 31.40 (NCH_2_CH_2_CH_2_), 28.03 (2×CH_2_). Anal. Calcd. for C_21_H_23_N_5_O_4_S: C, 57.13; H, 5.25; N, 15.86; found: C, 57.38; H, 5.39; N, 15.64%. HRMS (ESI) *m/z* for C_21_H_24_N_5_O_4_S^+^ [M + H]^+^, calculated: 442.1544, found: 442.1536. HPLC purity: 98.04% (Rt = 23.75 min).

{1‐[5‐(5‐Nitrofuran‐2‐yl)‐1,3,4‐thiadiazol‐2‐yl]piperidin‐4‐yl}(pyrrolidin‐1‐yl)methanone (**12**): Orange solid, mp 243°C–245°C. IR (KBr) (*ν*_max_/cm^–1^): 3298 (NH), 1643 (CO), 1595, 1498, 1434, 1398, 1366, 1346, 1291, 1226, 1181, 1139, 1085, 1014, 991, 956, 839. ^1^H NMR (300.1 MHz, CDCl_3_): *δ* 7.43 (d, *J* = 3.9 Hz, 1H, CH‐furan), 7.15 (d, *J* = 3.9 Hz, 1H, CH‐furan), 4.20–4.00 (m, 2H, 2CHN‐piperidine), 3.60–3.45 (m, 4H, 2CH_2_N‐pyrrolidine), 3.43–3.23 (m, 2H, 2CHN‐piperidine), 2.75–2.50 (m, 1H, CH‐piperidine), 2.15–1.75 (m, 8H, 2CH_2_‐piperidine and 2CH_2_‐pyrrolidine). ^13^C NMR (125.1 MHz, CDCl3) δ 172.80 (C), 172.14 (C), 151.63 (CO), 148.48 (C), 145.60 (C), 113.87 (CH), 110.36 (CH), 49.84 (2 × CH_2_N‐piperidine), 46.47 (CH_2_N‐pyrrolidine), 45.97 (CH_2_N‐pyrrolidine), 39.64 (CH), 27.20 (2×CH_2_‐piperidine), 26.20 (CH_2_‐pyrrolidine), 24.22 (CH_2_‐pyrrolidine). Anal. Calcd. for C_16_H_19_N_5_O_4_S: C, 50.92; H, 5.07; N, 18.56.; found: C, 51.09; H, 5.35; N, 18.78%. HRMS (ESI) *m/z* for C_16_H_20_N_5_O_4_S^+^ [M + H]^+^, calculated: 378.1231, found: 378.1241. HPLC purity: 99.87% (Rt = 28.86 min).

{1‐[5‐(5‐Nitrofuran‐2‐yl)‐1,3,4‐thiadiazol‐2‐yl]piperidin‐4‐yl}(piperidin‐1‐yl)methanone (**13**): Orange solid, mp 252°C–254°C. IR (KBr) (*ν*_max_/cm^–1^): 3287 (NH), 1636 (CO), 1537, 1503, 1466, 1443, 1385, 1372, 1350, 1309, 1258, 1225, 1209, 1184, 1156, 1091, 1011, 973, 810. ^1^H NMR (300.1 MHz, CDCl_3_): *δ* 7.43 (d, *J* = 3.9 Hz, 1H, CH‐furan), 7.15 (d, *J* = 3.9 Hz, 1H, CH‐furan), 4.12 (t, *J* = 3.4 Hz, 2H, CH_2_N), 4.07 (t, *J* = 3.6 Hz, 2H, CH_2_N), 3.56 (t, *J* = 4.9 Hz, 2H, CH_2_N), 3.47 (t, *J* = 4.3 Hz, 2H, CH_2_N), 3.40–3.20 (m, 2H, CH_2_N), 2.95–2.74 (m, 1H, CH‐piperidine), 2.10–1.80 (m, 4H, 2CH_2_‐piperidine), 1.72–1.45 (m, 6H, 3CH_2_‐piperidine). ^13^C NMR (75.1 MHz, CDCl_3_) δ 172.80 (C), 171.73 (C), 151.61 (CO), 148.47 (C), 145.64 (C), 113.85 (CH), 110.31 (CH), 49.80 (2 × CH_2_N), 46.55 (2 × CH_2_N), 42.94 (CH), 27.69 (2 × CH_2_), 26.85 (2 × CH_2_), 25.55 (2 × CH_2_), 24.54 (2 × CH_2_). Anal. Calcd. for C_17_H_21_N_5_O_4_S: C, 52.16; H, 5.41; N, 17.89; found: C, 52.32; H, 5.26; N, 18.13%. HRMS (ESI) *m/z* for C_17_H_22_N_5_O_4_S^+^ [M + H]^+^, calculated: 392.1387, found: 392.1368. HPLC purity: 98.00% (Rt = 26.75 min).

(4‐Methylpiperidin‐1‐yl){1‐[5‐(5‐nitrofuran‐2‐yl)‐1,3,4‐thiadiazol‐2‐yl]piperidin‐4‐yl}methanone (**14**): Orange solid, mp 268°C–270°C. IR (KBr) (*ν*_max_/cm^–1^): 3312 (NH), 1644 (CO), 1538, 1500, 1447, 1385, 1372, 1355, 1313, 1283, 1259, 1236, 1216, 1195, 1158, 1098, 1066, 1014, 974, 893. ^1^H NMR (300.1 MHz, CDCl_3_): *δ* 7.43 (d, *J* = 3.8 Hz, 1H, CH‐furan), 7.15 (d, *J* = 3.8 Hz, 1H, CH‐furan), 4.18–4.00 (m, 2H, 2CHN‐piperidine), 3.50–3.20 (m, 2H, 2CHN‐piperidine), 3.06 (t, *J* = 7.0 Hz, 2H, CH_2_N‐piperidine), 2.85–2.70 (m, 1H, CH‐piperidine), 2.56 (t, *J* = 7.2 Hz, 2H, CH_2_N), 2.10–1.50 (m, 9H, 4CH_2_ and CH‐CH_3_), 0.97 (d, *J* = 6.3 Hz, 3H, CH_3_). ^13^C NMR (125.1 MHz, DMSO‐d6) δ 172.75 (C), 171.98 (C), 151.93 (CO), 147.91 (C), 145.47 (C), 115.62 (CH), 112.33 (CH), 49.99 (2 × CH_2_N), 45.44 (2 × CH_2_N), 41.90 (CH), 30.98 (2 × CH_2_), 28.08 (CHCH_3_), 27.93 (2 × CH_2_), 22.08 (CH_3_). Anal. Calcd. for C_18_H_23_N_5_O_4_S: C, 53.32; H, 5.72; N, 17.27; found: C, 53.59; H, 5.58; N, 17.13%. HRMS (ESI) *m/z* for C_18_H_24_N_5_O_4_S^+^ [M + H]^+^, calculated: 406.1544, found: 406.1531. HPLC purity: 99.86% (Rt = 25.51 min).

[1,4′‐Bipiperidin]‐1′‐yl{1‐[5‐(5‐nitrofuran‐2‐yl)‐1,3,4‐thiadiazol‐2‐yl]piperidin‐4‐yl}methanone (**15**): Yellow solid, mp 288°C–291°C. IR (KBr) (*ν*_max_/cm^–1^): 3264 (NH), 1648 (CO), 1597, 1546, 1523, 1502, 1437, 1411, 1379, 1358, 1329, 1292, 1256, 1143, 1077, 1035, 991, 943, 869, 847. ^1^H NMR (500.1 MHz, DMSO‐*d*_6_) *δ* 7.85 (d, *J* = 3.9 Hz, 1H, CH‐furan), 7.36 (d, *J* = 3.9 Hz, 1H, CH‐furan), 4.10–3.90 (m, 2H, 2CHN‐piperidine), 3.52–3.17 (m, 10H, 5CH_2_), 3.05–2.93 (m, 2H, 2CH) 2.48–2.33 (m, 8H, 4CH_2_), 1.90–1.50 (m, 6H, 3CH_2_). ^13^C NMR (125.1 MHz, DMSO‐d6) δ 172.76 (C), 171.96 (C), 151.93 (CO), 147.90 (C), 145.48 (C), 115.63 (CH), 112.36 (CH), 50.14 (2 × CH_2_N), 49.99 (2 × CH_2_N), 44.79 (2 × CH_2_N), 43.51 (CHN), 41.32 (CHCO), 29.12 (2 × CH_2_), 27.93 (2 × CH_2_), 26.54 (2 × CH_2_), 25.02 (CH_2_). Anal. Calcd. for C_22_H_30_N_6_O_4_S: C, 55.68; H, 6.37; N, 17.71; found: C, 55.89; H, 6.66; N, 17.96%. HRMS (ESI) *m/z* for C_22_H_31_N_6_O_4_S^+^ [M + H]^+^, calculated: 475.2122, found: 475.2128. HPLC purity: 99.98% (Rt = 24.05 min).

Morpholino{1‐[5‐(5‐nitrofuran‐2‐yl)‐1,3,4‐thiadiazol‐2‐yl]piperidin‐4‐yl}methanone (**16**): Yellow solid, mp 269°C–271°C. IR (KBr) (*ν*_max_/cm^–1^): 3329 (NH), 1638 (CO), 1593, 1521, 1498, 1445, 1413, 1368, 1323, 1302, 1294, 1231, 1179, 1143, 1087, 1017, 988, 845. ^1^H NMR (300.1 MHz, CDCl_3_): *δ* 7.42 (d, *J* = 3.6 Hz, 1H, CH‐furan), 7.15 (d, *J* = 3.6 Hz, 1H, CH‐furan), 4.17–3.97 (m, 2H, 2CHN‐piperidine), 3.75–3.48 (m, 8H, 4CH_2_‐morpholine), 3.40–3.20 (m, 2H, 2CHN‐piperidine), 2.90–2.70 (m, 1H, CH‐piperidine), 2.20–1.75 (m, 4H, 2CH_2_‐piperidine). ^13^C NMR (75.1 MHz, CDCl_3_) δ 172.74 (C), 172.18 (C), 151.58 (CO), 148.36 (C), 145.79 (C), 113.84 (CH), 110.40 (CH), 66.91 (CH_2_O), 66.72 (CH_2_O), 49.67 (2 × CH_2_N‐piperidine), 46.01 (2 × CH_2_N‐morpholine), 42.14 (CH), 27.55 (2 × CH_2_‐piperidine). Anal. Calcd. for C_16_H_19_N_5_O_5_S: C, 48.85; H, 4.87; N, 17.80; found: C, 49.03; H, 4.55; N, 17.68%. HRMS (ESI) *m/z* for C_16_H_20_N_5_O_5_S^+^ [M + H]^+^, calculated: 394.1180, found: 394.1190. HPLC purity: 96.12% (Rt = 33.10 min).

(4‐Methylpiperazin‐1‐yl){1‐[5‐(5‐nitrofuran‐2‐yl)‐1,3,4‐thiadiazol‐2‐yl]piperidin‐4‐yl}methanone (**17**): Orange solid, mp 276°C–279°C. IR (KBr) (*ν*_max_/cm^–1^): 3196 (NH), 1644 (CO), 1538, 1500, 1447, 1385, 1372, 1355, 1283, 1258, 1236, 1216, 1195, 1134, 1098, 1066, 1014, 988, 974, 893. ^1^H NMR (500.1 MHz, CDCl_3_): *δ* 7.45 (d, *J* = 3.9 Hz, 1H, CH‐furan), 7.18 (d, *J* = 3.9 Hz, 1H, CH‐furan), 4.22–4.10 (m, 2H, 2CHN‐piperidine), 3.73–3.65 (m, 2H, CH_2_N‐piperazine), 3.62–3.52 (m, 2H, CH_2_N‐piperazine), 3.42–3.28 (m, 2H, 2CHN‐piperidine), 2.88–2.75 (m, 1H, CH‐piperidine), 2.50–2.38 (m, 4H, 2CH_2_N‐piperazine), 2.35 (s, 3H, CH_3_), 2.12–1.95 (m, 2H, CH_2_‐piperdine), 1.93–1.80 (m, 2H, CH_2_‐piperdine). ^13^C NMR (125.1 MHz, DMSO‐d6) δ 172.74 (C), 172.67 (C), 151.93 (CO), 147.86 (C), 145.57 (C), 115.62 (CH), 112.38 (CH), 52.82 (2 × CH_2_N‐piperazine), 49.89 (2 × CH_2_N‐piperidine), 42.72 (2 × CH_2_N‐piperazine), 42.39 (CH), 36.47 (CH_3_), 27.78 (CH). Anal. Calcd. for C_17_H_22_N_6_O_4_S: C, 50.23; H, 5.46; N, 20.68.; found: C, 49.99; H, 5.69; N, 20.55%. HRMS (ESI) *m/z* for C_17_H_23_N_6_O_4_S^+^ [M + H]^+^, calculated: 407.1496, found: 407.1513. HPLC purity: 99.93% (Rt = 33.52 min).

*N*‐Cyclohexyl‐1‐[5‐(5‐nitrofuran‐2‐yl)‐1,3,4‐thiadiazol‐2‐yl]piperidine‐4‐carboxamide (**18**): Orange solid, mp 248°C–252°C. IR (KBr) (*ν*_max_/cm^–1^): 3285 (NH), 1659 (CO), 1582, 1534, 1414, 1367, 1325, 1309, 1293, 1232, 1172, 1132, 1080, 1014, 1080, 1014, 991, 937, 901, 823. ^1^H NMR (300.1 MHz, CDCl_3_): *δ* 7.44 (d, *J* = 3.7 Hz, 1H, CH‐furan), 7.16 (d, *J* = 3.7 Hz, 1H, CH‐furan), 5.38 (d, *J* = 6.4 Hz, NH‐amide), 4.25–4.00 (m, 2H, 2CHN‐piperidine), 3.85–3.60 (m, 1H, CH‐cyclohexyl), 3.38–3.17 (m, 2H, 2CHN‐piperidine), 2.50–2.25 (m, 1H, CH‐piperidine), 2.10–1.53 (m, 10H, 5CH_2_‐cyclohexyl), 1.50–0.95 (m, 4H, 2CH_2_‐piperidine). ^13^C NMR (125.1 MHz, DMSO‐d6) δ 172.92 (C), 172.81 (C), 151.93 (CO), 147.89 (C), 145.48 (C), 115.62 (CH), 112.34 (CH), 50.10 (2 × CH_2_N), 47.73 (CHN), 41.28 (CHCO), 32.89 (2 × CH_2_), 28.04 (2 × CH_2_), 25.71 (CH_2_), 25.04 (2 × CH_2_). Anal. Calcd. for C_18_H_23_N_5_O_4_S: C, 53.32; H, 5.72; N, 17.27; found: C, 53.08; H, 5.48; N, 17.64%. HRMS (ESI) *m/z* for C_18_H_24_N_5_O_4_S^+^ [M + H]^+^, calculated: 406.1544, found: 406.1536. HPLC purity: 96.86% (Rt = 23.75 min).

*N*‐Ethyl‐1‐[5‐(5‐nitrofuran‐2‐yl)‐1,3,4‐thiadiazol‐2‐yl]piperidine‐4‐carboxamide (**19**): Orange solid, mp 217°C–220°C. IR (KBr) (*ν*_max_/cm^–1^): 3245 (NH), 1634 (CO), 1550, 1515, 1496, 1459, 1442, 1372, 1357, 1343, 1331, 1310, 1274, 1255, 1212, 1178, 1090, 1013, 937. ^1^H NMR (300.1 MHz, CDCl_3_): *δ* 7.43 (d, *J* = 3.9 Hz, 1H, CH‐furan), 7.13 (d, *J* = 3.9 Hz, 1H, CH‐furan), 5.82 (t, *J* = 5.3 Hz, 1H, NH‐amide), 4.15–4.00 (m, 2H, 2CHN‐piperidine), 3.40–3.20 (m, 4H, CH_2_NH and 2CHN‐piperidine), 2.46–2.34 (m, 1H, CH‐piperidine), 2.08–1.75 (m, 4H, 2CH_2_‐piperidine), 1.13 (t, *J* = 7.2 Hz, 3H, CH_3_). ^13^C NMR (75.1 MHz, CDCl3) δ 173.33 (C), 172.73 (C), 151.60 (CO), 148.34 (C), 145.69 (C), 113.90 (CH), 110.43 (CH), 49.86 (2 × CH_2_N), 42.23 (CH), 34.41 (NCH_2_CH_3_), 27.83 (2 × CH_2_), 14.81 (NCH_2_CH_3_). Anal. Calcd. for C_14_H_17_N_5_O_4_S: C, 47.85; H, 4.88; N, 19.93; found: C, 48.12; H, 5.08; N, 19.67%. HRMS (ESI) *m/z* for C_14_H_18_N_5_O_4_S^+^ [M + H]^+^, calculated: 352.1074, found: 352.1087. HPLC purity: 99.93% (Rt = 31.13 min).

*N*‐Isopropyl‐1‐[5‐(5‐nitrofuran‐2‐yl)‐1,3,4‐thiadiazol‐2‐yl]piperidine‐4‐carboxamide (**20**): Orange solid, mp 228°C–230°C. IR (KBr) (*ν*_max_/cm^–1^): 3251 (NH), 1653 (CO), 1544, 1499, 1458, 1385, 1370, 1354, 1316, 1276, 1258, 1228, 1207, 1156, 1100, 1011, 969, 802. ^1^H NMR (500.1 MHz, DMSO‐*d*_6_) *δ* 7.45 (d, *J* = 3.8 Hz, 1H, CH‐furan), 7.18 (d, *J* = 3.8 Hz, 1H, CH‐furan), 5.30 (d, *J* = 6.8 Hz, 1H, NH‐amide), 4.16–4.06 (m, 2H, 2CHN‐piperidine), 3.40–3.26 (m, 3H, CHNH and 2CHN‐piperidine), 2.43–2.28 (m, 1H, CH‐piperidine), 2.05–1.88 (m, 4H, 2CH_2_‐piperidine), 1.19 (d, *J* = 6.5 Hz, 6H, 2CH_3_). ^13^C NMR (125.1 MHz, DMSO‐d6) δ 172.90 (C), 171.64 (C), 151.92 (CO), 147.87 (C), 145.48 (C), 115.60 (CH), 112.34 (CH), 50.08 (2 × CH_2_N), 42.67 (CHCO), 41.30 (CHNH), 27.96 (2 × CH_2_), 22.82 (2CH_3_). Anal. Calcd. for C_15_H_19_N_5_O_4_S: C, 49.30; H, 5.24; N, 19.17.; found: C, 49.18; H, 5.46; N, 19.40%. HRMS (ESI) *m/z* for C_15_H_20_N_5_O_4_S^+^ [M + H]^+^, calculated: 367.1304, found: 367.1312. HPLC purity: 99.26% (Rt = 28.93 min).

### 4.2 Biological Assessments

#### 4.2.1 Cytotoxicity Assay

THP‐1 cells (human acute monocytic leukemia cell line) were maintained in RPMI supplemented with 10% FBS (EuroClone), 50 µM 2‐mercaptoethanol, 20 mM Hepes, 2 mM glutamine, at 37°C in 5% CO_2_. THP‐1 cells were plated at 5 × 10^4^ cells/well in 96 wells flat bottom microplates and treated with 0.1 µM phorbol myristate acetate (PMA; Sigma) for 48 h to achieve differentiation into macrophages. Cells were treated with serial dilutions of test compounds for 72 h and cell viability evaluated by the MTT assay. The results are expressed as CC_50_ (cytotoxic concentration of the compound that cause death to 50% of viable cells). Each CC_50_ value is the mean and standard deviation of at least three separate experiments performed in duplicate.

#### 4.2.2 In Vitro Activity Against L. infantum and L. tropica Promastigote Stage

Promastigote stages of *L. infantum* (MHOM/TN/80/IPT1) and *L. tropica* (MHOM/SY/2012/ISS3130) were cultured in Schneider's Drosophila medium supplemented with 2 mM l‐glutamine and 10% heat inactivated FBS (HyClone) at 23°C. To estimate the 50% inhibitory concentration (IC_50_), the MTT (3‐[4.5‐dimethylthiazol‐2‐yl]‐2.5‐diphenyltetrazolium bromide) method was used with modifications [42, 43]. Compounds were dissolved in DMSO and then diluted with complete medium (RPMI1640 supplemented with 10% heat‐inactivated calf serum, 20 mM Hepes, 2 mM l‐Glutamine) to achieve the required concentrations. Drugs were placed in 96 wells round‐bottom microplates and seven serial dilutions made. Amphotericin B was used as the reference antileishmanial drug. Parasites were diluted in complete medium to 5 × 10^6^ parasites/mL and 100 μL of the suspension was seeded into the plates, incubated at 23°C for 72 h and then 20 µL of MTT solution (5 mg/mL in PBS) was added into each well for 3 h. The plates were then centrifuged, the supernatants discarded and the resulting pellets dissolved in 100 µL of lysing buffer consisting of 20% (w/v) of a solution of SDS (Sigma), 40% of N,N‐dimethylformamide (Merck) in H_2_O. The absorbance was measured spectrophotometrically at a test wavelength of 550 nm and a reference wavelength of 650 nm. The results are expressed as IC_50_ which is the dose of compound necessary to inhibit cellular growth by 50%; each IC_50_ value is the mean ± standard deviation of at least three separate experiments performed in duplicate.

#### 4.2.3 In Vitro Assay Against Intramacrophage Amastigotes

THP‐1 cells were plated at 5 × 10^5^ cells/mL in 16 Lab‐Tek culture slides (Nunc) in 100 µL and treated with 0.1 µM PMA for 72 h to achieve differentiation into macrophages. Cells were then washed and infected with stationary phase *L. infantum* promastigotes at a macrophage/promastigote ratio of 1/10 for 24 h. Cell monolayers were then washed to remove non internalized parasites and incubated in the presence of different concentrations of test compounds for 72 h. Slides were fixed with methanol and stained with Giemsa. The percentage of infected macrophages in treated and non‐treated cells was determined by light microscopy [43].

#### 4.2.4 In Vitro Activity Against *T. b. gambiense*, *T. b. brucei*, and *T. congolense* Bloodstreams

The bloodstream forms of *T. b. gambiense* AnTat 1.3 and *T. b. brucei* 427 were grown in HMI‐11 medium supplemented with 10% fetal bovine serum at 37°C and 5% CO_2_ [44]. The bloodstream form of *T. congolense* was cultured in TcBSF‐1 medium supplemented with 20% goat serum at 34°C, 5% CO_2_. The assay for antitrypanosomal activity was performed using the resazurin sodium salt dye (Alamar Blue Assay) according to the published protocol in a 96‐well plate format [45]. Parasites at a number of 5 × 10^3^ per well were incubated with different drug concentrations (2‐fold serial dilutions) in a volume of 200 μL of the medium. The plates were incubated for 48 h at the appropriate temperature. Then, 20 μL of resazurin sodium salt solution (0.125 mg/mL in 1× PBS, pH 7. 4) was added to each well, and the cells were incubated for another 24 h under the same conditions. The fluorescence signal was quantified using a Tecan Infinite M200 plate reader at excitation and emission wavelengths of 560 and 590 nm, respectively. The IC_50_ were calculated using GraphPad Prism 8.0 by nonlinear regression with a variable slope. Each IC_50_ value is the mean ± standard deviation of three separate experiments performed in duplicates.

#### 4.2.5 Cellular and Mitochondrial ROS Assessment

The generation of intracellular and intramitochondrial reactive oxygen species (ROS) was evaluated in accordance with established protocols. To assess intracellular ROS levels, three biological replicates of 5 × 10⁶ untreated *BF T. b. brucei* cells and those treated with the specific concentration of the drug for 24 h were analyzed. The concentrations of the compounds utilized were 0.5×, 1×, 2×, and 4× of the EC_50_ concentrations obtained from the Alamar Blue assays (**10**: EC_50_ 0.38 μM; **11**: EC_50_ 0.39 μM; **15**: EC_50_ 0.17 μM; **18**: EC_50_ 0.31 μM). Following incubation with the compounds, the samples were collected and incubated for 30 min with 10 μM 2′,7′‐dichlorofluorescein diacetate (Sigma), after which they were washed with PBS. A total of 10,000 events were recorded on the BD FACSCanto II instrument using a 488 nm excitation laser and a 530/30 nm detector. To assess mitochondrial ROS production, the MitoSOX indicator (Thermo Fisher Scientific) was employed in both untreated cells and cells subjected to drug treatment. The same compounds used to study intracellular ROS were utilized to assess mitochondrial ROS levels. An equivalent number of cells (5 × 10⁶) for each treatment group was collected, resuspended in HMI‐9 medium containing 5 μM MitoSOX (ThermoFisher), and stained for 30 min at 37°C in a 5% CO₂ environment. Following staining, cells were subjected to centrifugation, resuspension in PBS, and subsequent analysis by flow cytometry using the BD FACS Canto II instrument with a 488 nm excitation laser and a 585/15 nm emission filter. For each sample, 10,000 events were collected. All data were subsequently analyzed using Prism (10.3.1) from GraphPad Software.

#### 4.2.6 Dead Cell Apoptosis Assay

To assess the cytostatic or cytotoxic effect of the selected compounds on the *T. b. brucei* cell population, we performed live‐dead assay using Dead Cell Apoptosis Kit (Invitrogen, V13241). Briefly, 1 × 10^3^ cells/mL were treated with either **10** (EC_50_: 0.38 μM), **11** (EC_50_: 0.39 μM), **15** (EC_50_: 0.17 μM), and **18** (EC_50_: 0.31 μM) at the concentration 2× EC_50_ for 72 h. After the incubation, 1 × 10^6^ cells were collected and incubated in 100 µL of the provided 1× buffer with Alexa Fluor™ 488 Annexin V and PI for 15 min according to the user guide. As a positive control, prior to the staining procedure, 1 × 10^6^ cells were treated with 0.2% Triton for 10 min to induce cell death. The stained cells were analyzed by flow cytometry (BD FACS‐Canto II; BD Biosciences, USA), measuring fluorescence emission at 530 and 575 nm using 488‐nm excitation.

#### 4.2.7 Growth Curves

*T. b. brucei* BSF cell lines were maintained in 5 mL of HMI‐11 medium with 10% FBS at 37°C, 5% CO_2_. Cell density was measured using a Beckman Z2 Cell Counter and cells were maintained in their exponential mid‐log growth phase at between 1 × 10^5^–1 × 10^6^ cells/mL. The exponential growth curves were plotted using the GraphPad Prism (10.4.2) software.

### 4.3 Computational Studies

Molecular docking simulations were performed using AutoDock4 (version 4.2.6). Protein crystal structures were obtained from the Protein Data Bank (PDB IDs: 3CLB, 3GN2, and 3RG9). Prior to docking, protein structures were prepared by removal of crystallographic water molecules, addition of polar hydrogen atoms, and assignment of Gasteiger partial charges following standard AutoDock protocols.

Docking grid boxes were defined to encompass the active‐site regions corresponding to the co‐crystallized ligands. For PDB ID 3CLB, a grid box of 40 × 40 × 40 points was centered at *X* = 21.069, *Y* = 38.486, *Z* = 24.450. For PDB ID 3GN2, grid dimensions of 56 × 52 × 44 points were used with a grid center at *X* = 2.495, *Y *= −12.399, *Z* = 17.164. For PDB ID 3RG9, a grid box of 58 × 50 × 58 points was centered at *X* = −21.959, *Y* = 21.552, *Z* = 8.856. These grid settings were selected to fully cover the binding cavities of the respective proteins.

Docking protocol validation was conducted by re‐docking the native ligands into their corresponding binding sites prior to docking of the test compounds. Ligand structures (**10** and **18**) were prepared and energy‐minimized prior to docking. Docking calculations were carried out using the Lamarckian Genetic Algorithm with 50 independent runs for each ligand–protein pair under default AutoDock4 parameters.

Docking poses were clustered based on root‐mean‐square deviation values, and the most populated clusters with the highest predicted binding energies were selected for further analysis.

## Conflicts of Interest

The authors declare no conflicts of interest.

## Supporting information


**Table 1.** Antileishmanial activities and mammalian cell cytotoxicity of compounds **8‐20**. **Table 2.** *In vitro* antileishmanial activities of compounds **8‐20** against intramacrophages *L. infantum* amastigotes. **Table 3.** *In vitro* antitrypanosomal activities of compounds **8‐20**.

## Data Availability

The data supporting the findings of this study are available within the article and its Supporting Information. Additional data is available from the corresponding author upon request.
