# Supplementary material for: Exploring Novel Nitrofuryl‐1,3,4‐Thiadiazole‐Based Derivatives: Design, Synthesis, and Evaluation of In Vitro Leishmanicidal and Trypanocidal Activity
Source: Arch Pharm (Weinheim). 2026 Mar 22;359(3):e70227. doi: 10.1002/ardp.70227 (PMC13006720; doi:10.1002/ardp.70227)
Supplement: Supplementary file 1 — Table 1. Antileishmanial activities and mammalian cell cytotoxicity of compounds 8‐20. Table 2. In vitro antileishmanial activities of compounds 8‐20 against intramacrophages L. infantum amastigotes. Table 3. In vitro antitrypanosomal activities of compounds 8‐20. [file ARDP-359-e70227-s001.docx]

**Exploring novel nitrofuryl-1,3,4-thiadiazole-based derivatives: design, synthesis, and evaluation of *in vitro* leishmanicidal and trypanocidal activity**

Alireza Mousavi ^a,b^, Martina Slapničková ^c^, Maryam Norouzbahari ^d^, Sarah D’Alessandro ^e^, Parham Foroumadi ^a^, Fariba Peytam ^f^, Federica Perego ^g^, Eva Doleželová ^c^, Zahra Emamgholipour ^b^, Maliheh Barazandeh Tehrani ^b^, Seyed Esmaeil Sadat-Ebrahimi ^a,b^, Elahe Hosseinzadeh ^f^, Elmira Meghrazi Ahadi ^b^, Loghman Firoozpour ^b,f^, Hamidreza Bijanzadeh ^h^, Nicoletta Basilico ^g,*^, Alena Zíková ^c,*^ , Alireza Foroumadi ^b,f,*^

^a^ International Campus-School of Pharmacy, Tehran University of Medical Sciences, Tehran, Iran

^b^ Department of Medicinal Chemistry, Faculty of Pharmacy, Tehran University of Medical Sciences, Tehran, Iran

^c^ Institute of Parasitology, Biology Centre, Czech Academy of Sciences, Ceske Budejovice, Czech Republic

^d^ Faculty of Pharmacy, Final International University, Catalkoy, Kyrenia via Mersin 10 Turkey, TRNC

^e^ Department of Pharmacological and Biomolecular Sciences “Rodolfo Paoletti”, University of Milan, Milan, Italy

^f^ Drug Design and Development Research Centre, The Institute of Pharmaceutical Sciences, Tehran University of Medical Sciences, Tehran, Iran

^g^ Department of Biomedical, Surgical and Dental Sciences, University of Milan, Milan, Italy

^h^ Department of Environmental Sciences, Faculty of Natural Resources and Marine Sciences, Tarbiat Modares University, Tehran, Iran

**Corresponding authors**: Dr. Nicoletta Basilico ([nicoletta.basilico@unimi.it](mailto:nicoletta.basilico@unimi.it)), Dr. Alena Zíková ([azikova@paru.cas.cz](mailto:azikova@paru.cas.cz)), and Dr. Alireza Foroumadi (aforoumadi@yahoo.com)

^1^H NMR spectrum of 1-(5-(5-nitrofuran-2-yl)-1,3,4-thiadiazol-2-yl)piperidine-4-carboxylic acid (**7**)


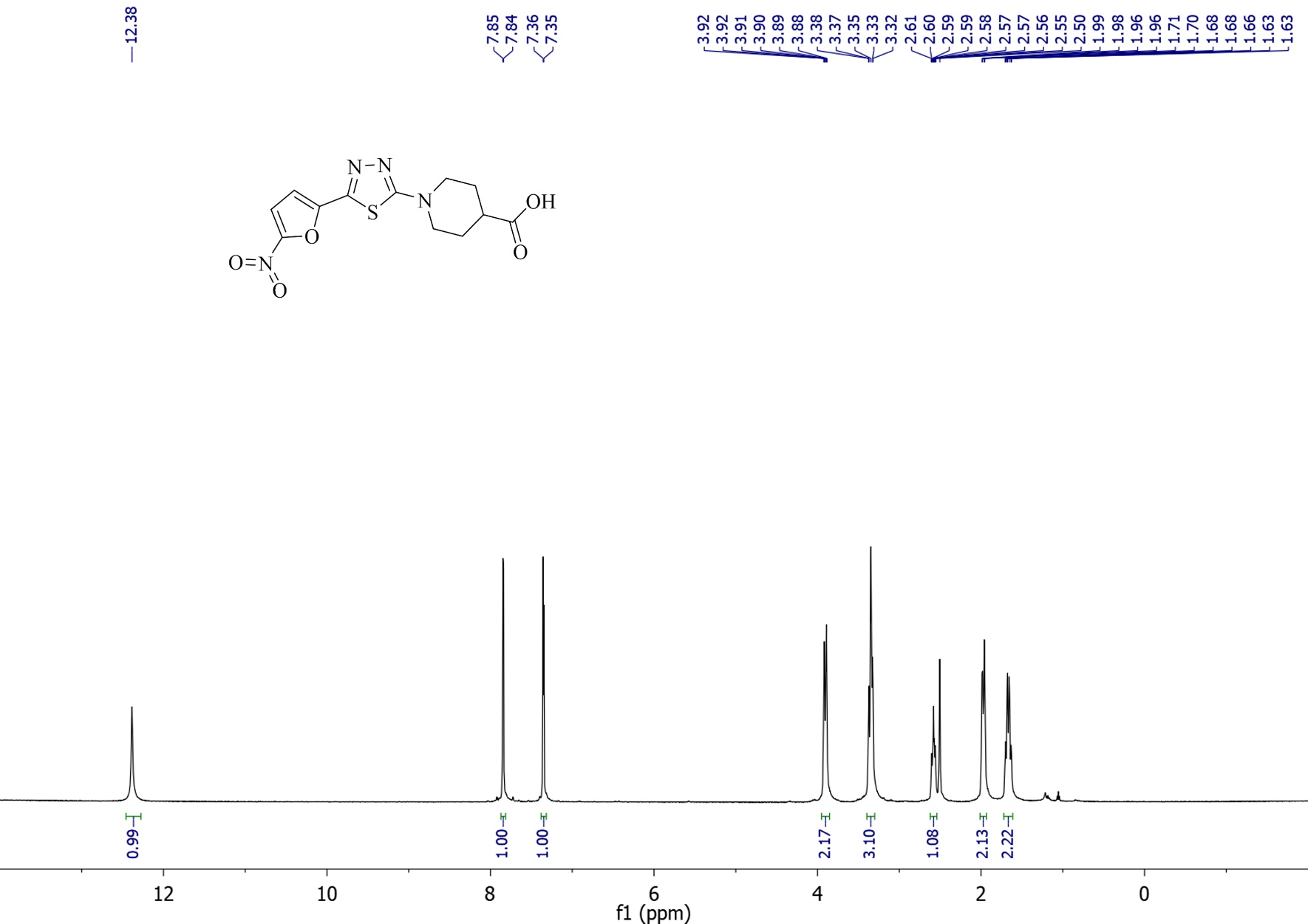


Expanded ^1^H NMR spectrum of 1-(5-(5-nitrofuran-2-yl)-1,3,4-thiadiazol-2-yl)piperidine-4-carboxylic acid (**7**)


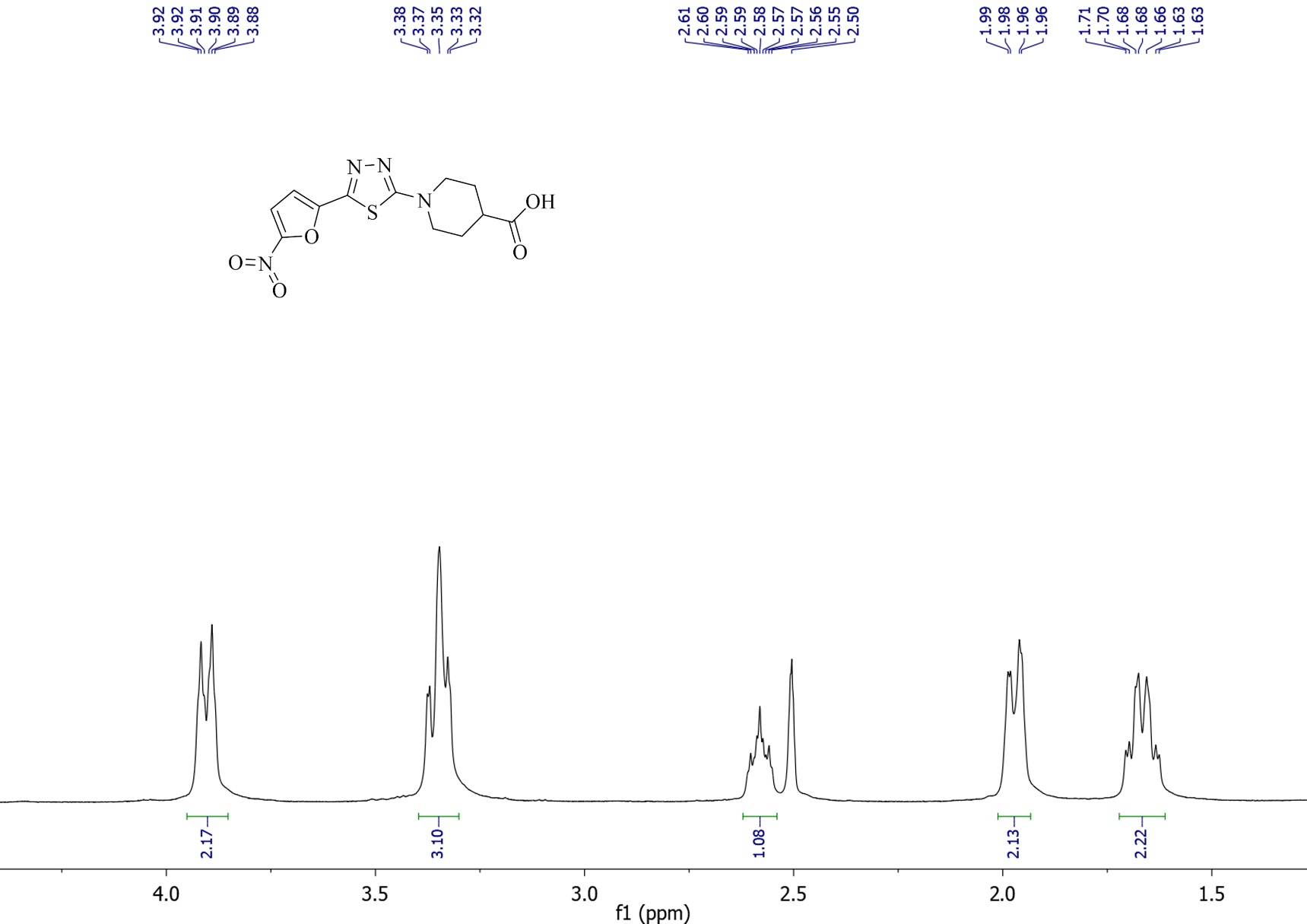


^13^C NMR spectrum of 1-(5-(5-nitrofuran-2-yl)-1,3,4-thiadiazol-2-yl)piperidine-4-carboxylic acid (**7**)


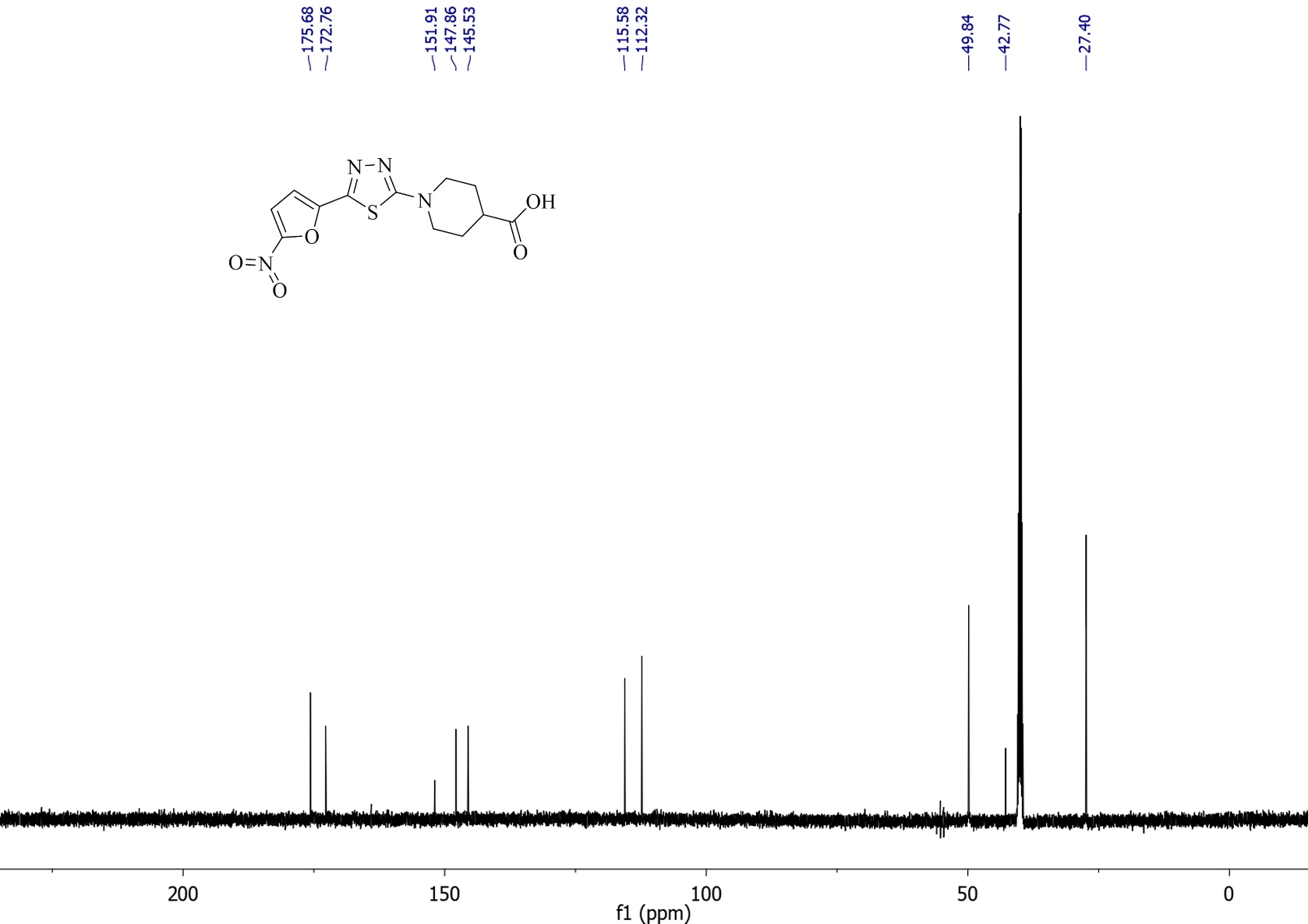


HRMS spectrum of 1-(5-(5-nitrofuran-2-yl)-1,3,4-thiadiazol-2-yl)piperidine-4-carboxylic acid (**7**)


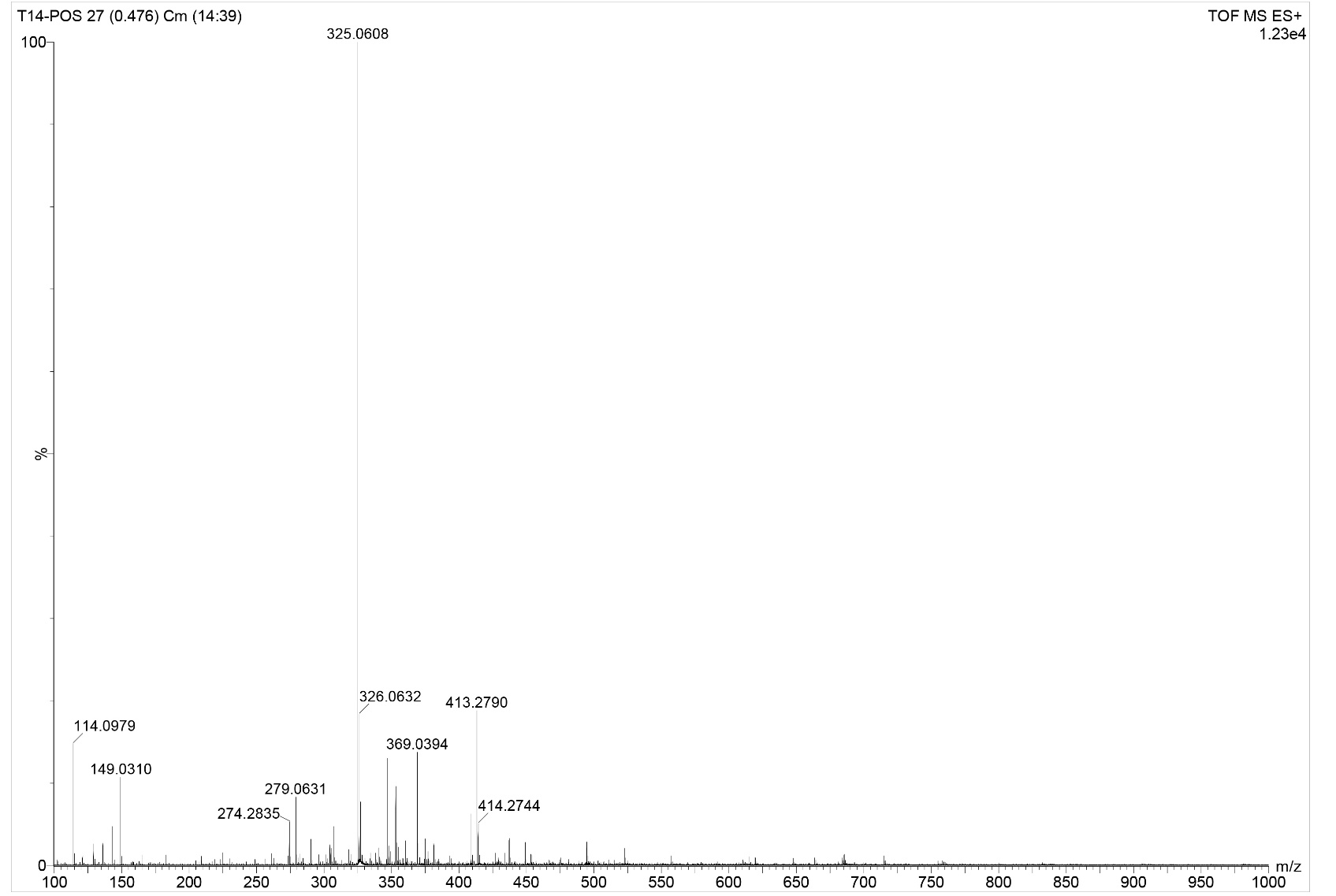


^1^H NMR spectrum of 1-(5-(5-nitrofuran-2-yl)-1,3,4-thiadiazol-2-yl)-*N*-phenylpiperidine-4-carboxamide (**8**)

^
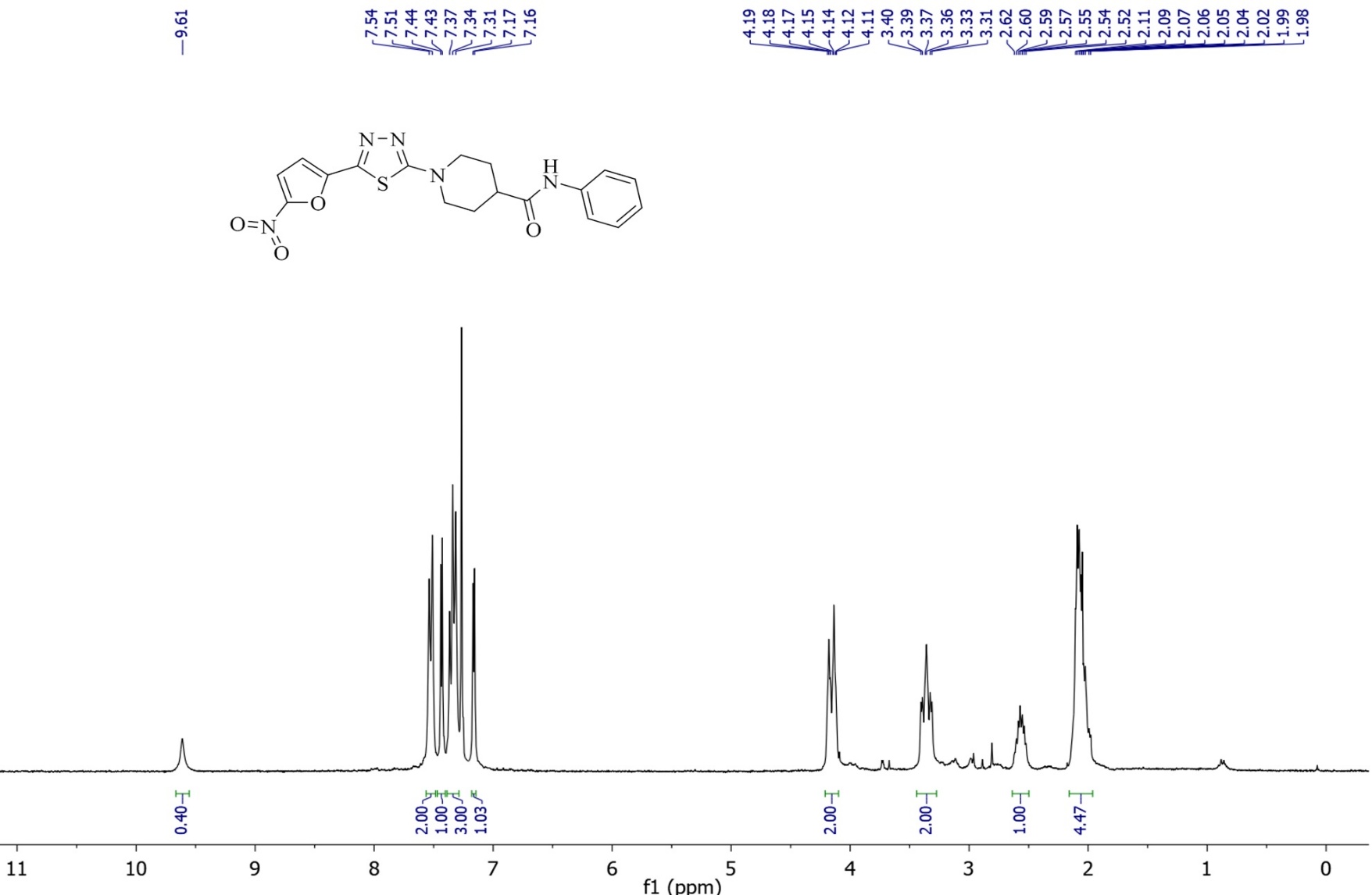
^Expanded ^1^H NMR spectrum of 1-(5-(5-nitrofuran-2-yl)-1,3,4-thiadiazol-2-yl)-*N*-phenylpiperidine-4-carboxamide (**8**)


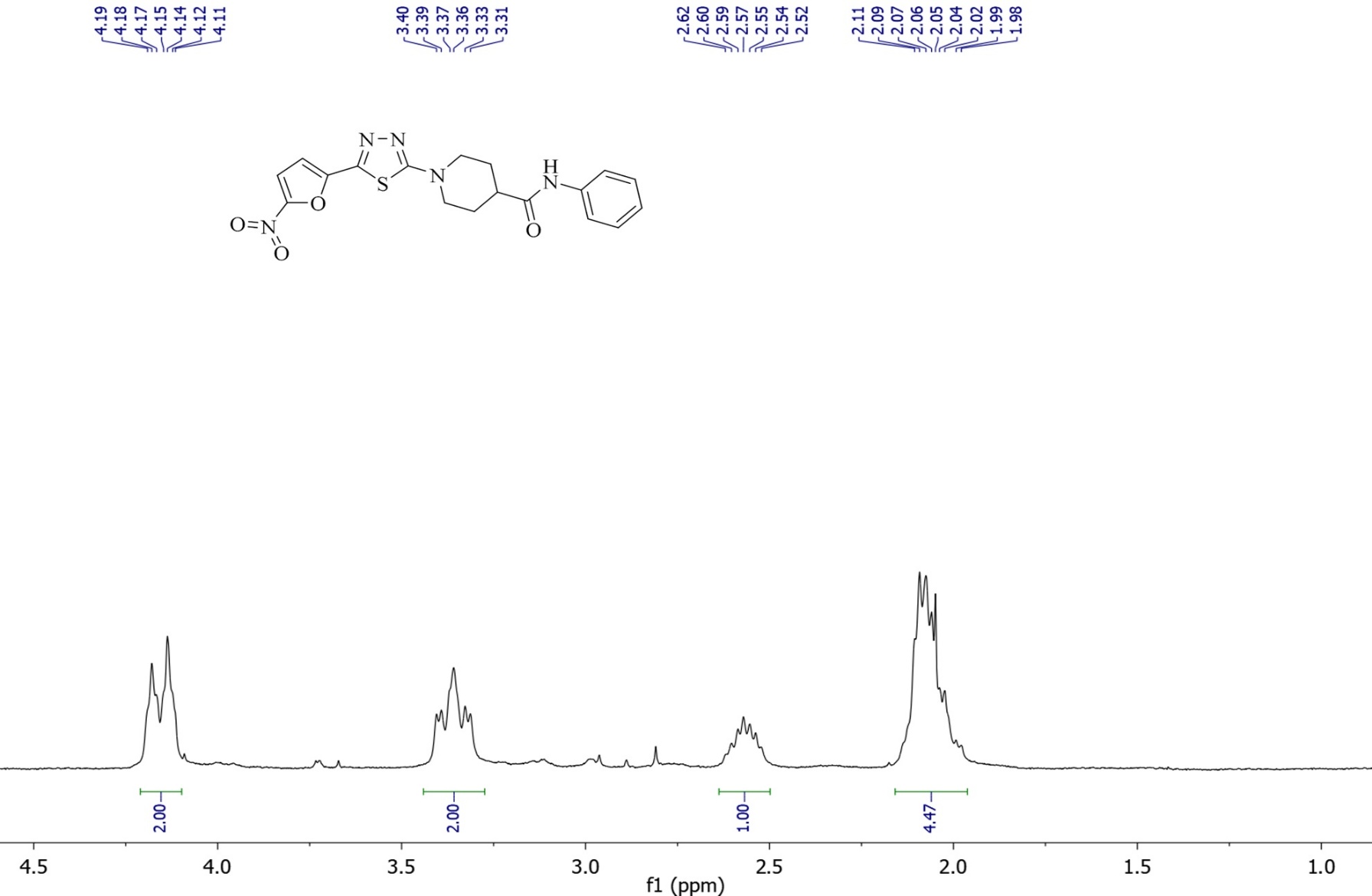


^13^C NMR spectrum of 1-(5-(5-nitrofuran-2-yl)-1,3,4-thiadiazol-2-yl)-*N*-phenylpiperidine-4-carboxamide (**8**)

^
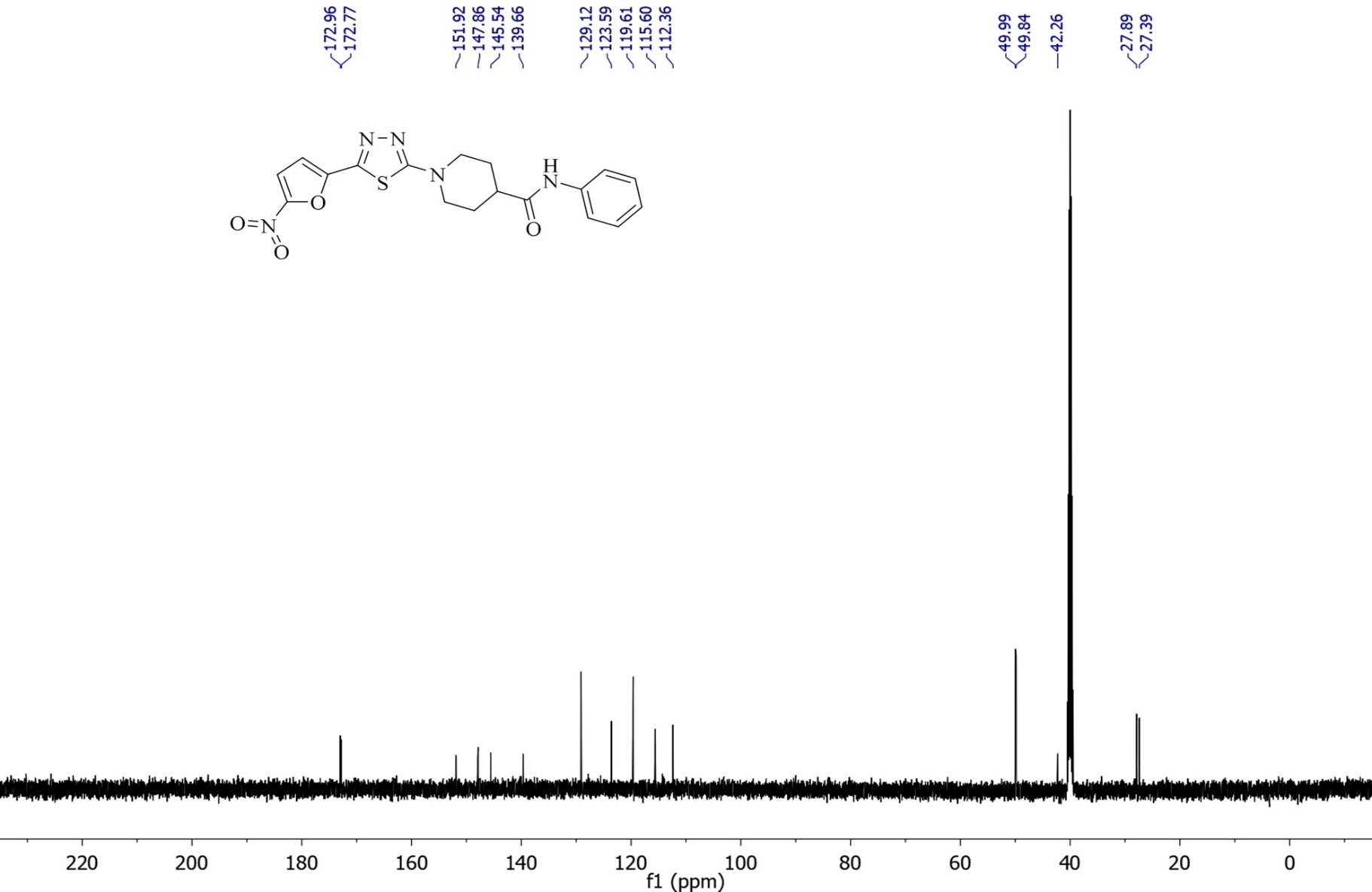
^HRMS spectrum of 1-(5-(5-nitrofuran-2-yl)-1,3,4-thiadiazol-2-yl)-*N*-phenylpiperidine-4-carboxamide (**8**)
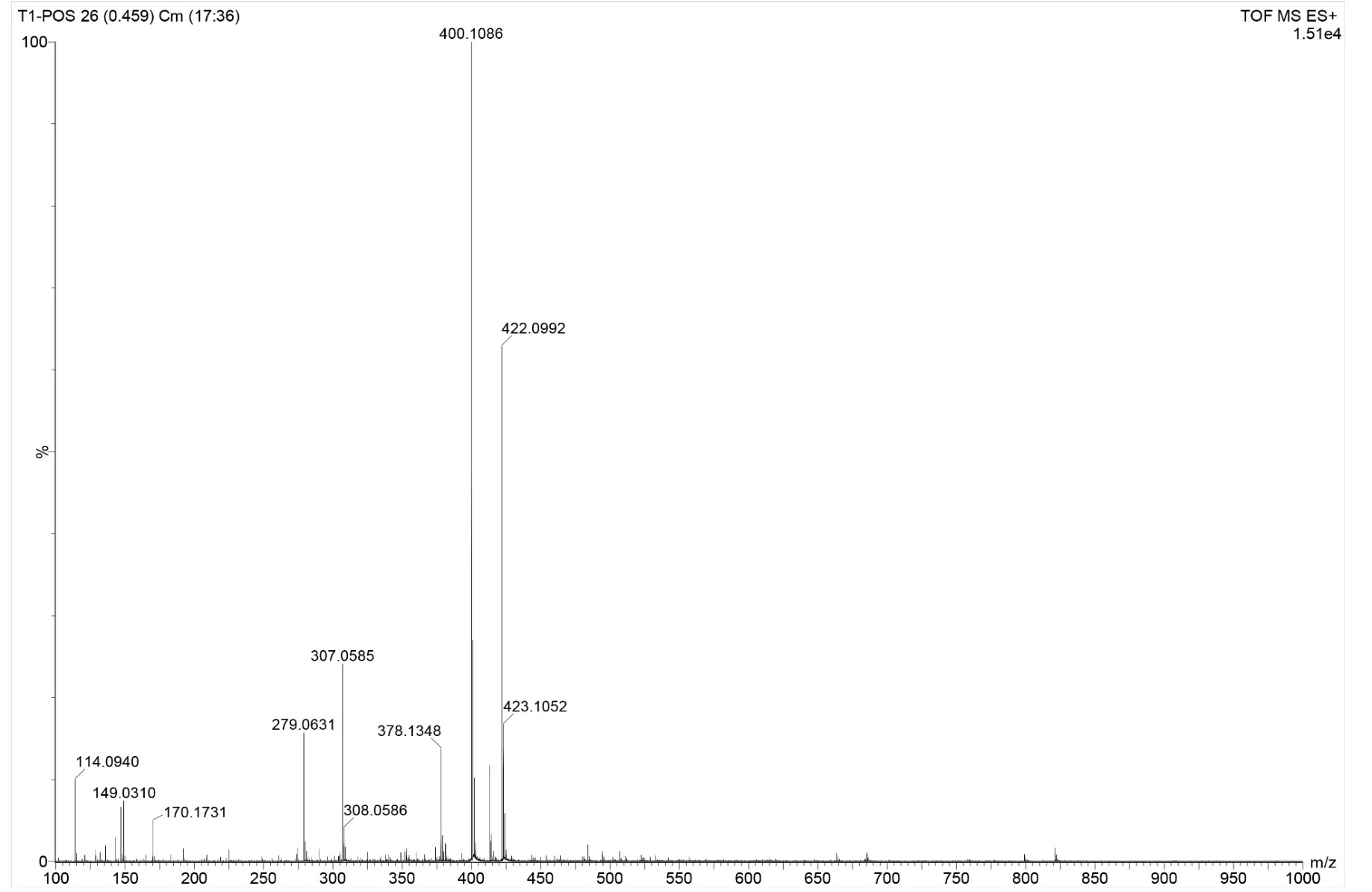


HPLC spectrum of 1-(5-(5-nitrofuran-2-yl)-1,3,4-thiadiazol-2-yl)-N-phenylpiperidine-4-carboxamide (**8**)

^
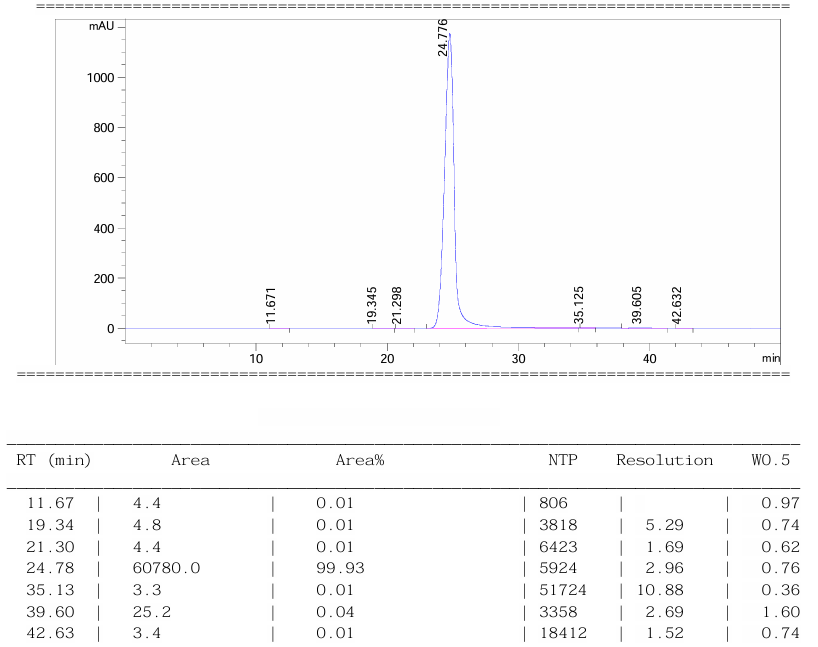
^


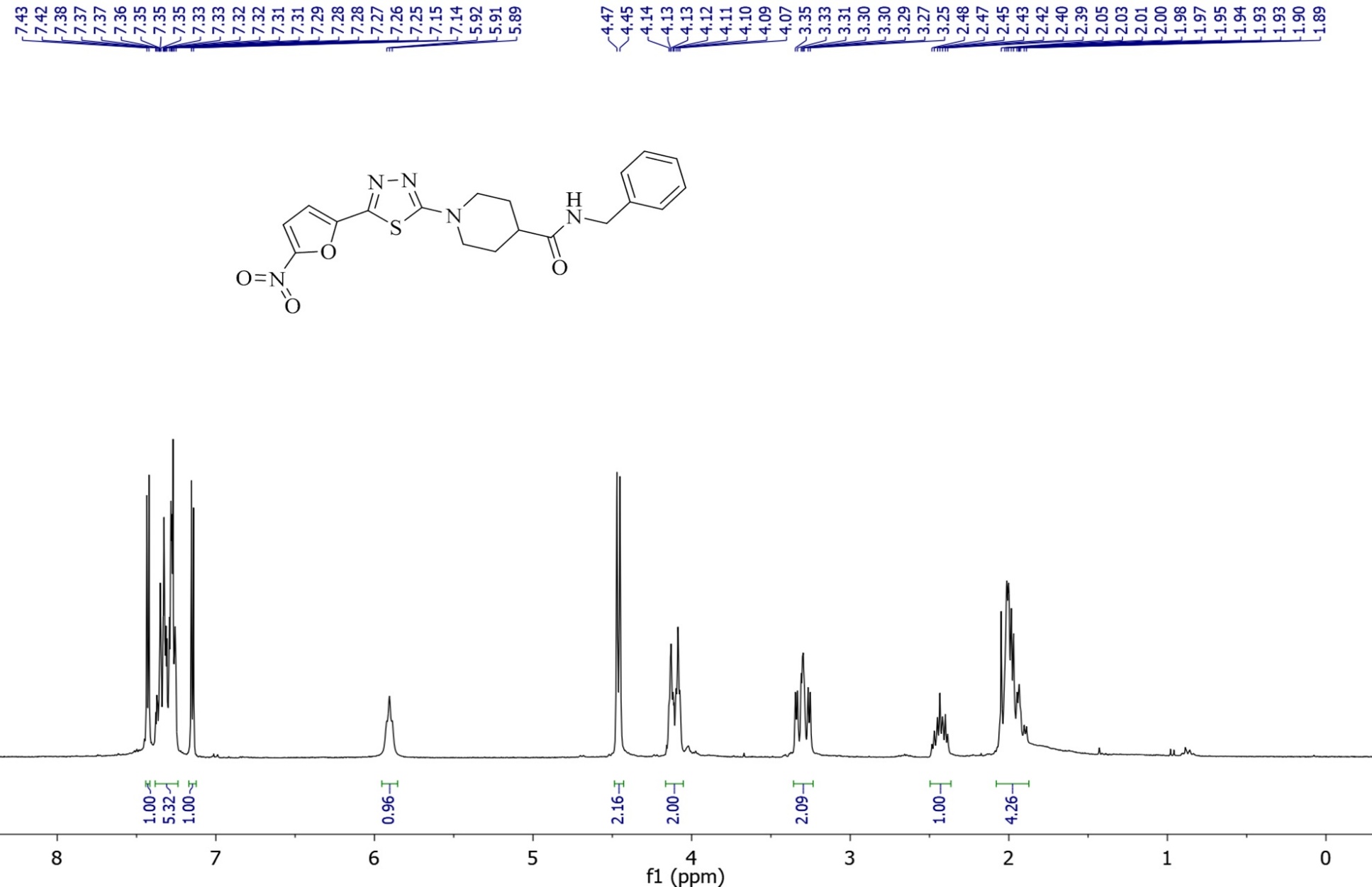
^1^H NMR spectrum of *N*-benzyl-1-(5-(5-nitrofuran-2-yl)-1,3,4-thiadiazol-2-yl)piperidine-4-carboxamide (**9**)

Expanded ^1^H NMR spectrum of *N*-benzyl-1-(5-(5-nitrofuran-2-yl)-1,3,4-thiadiazol-2-yl)piperidine-4-carboxamide (**9**)


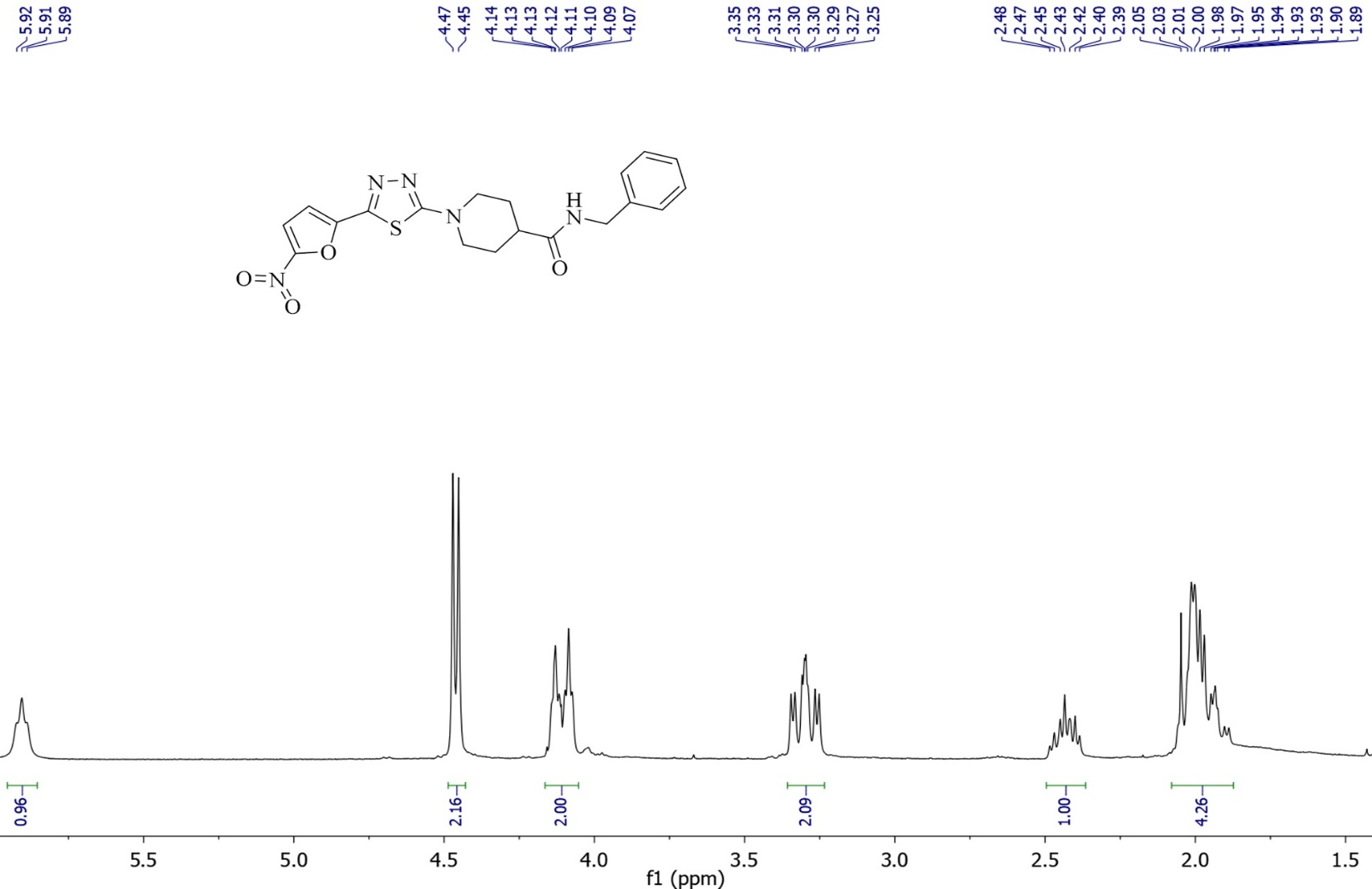


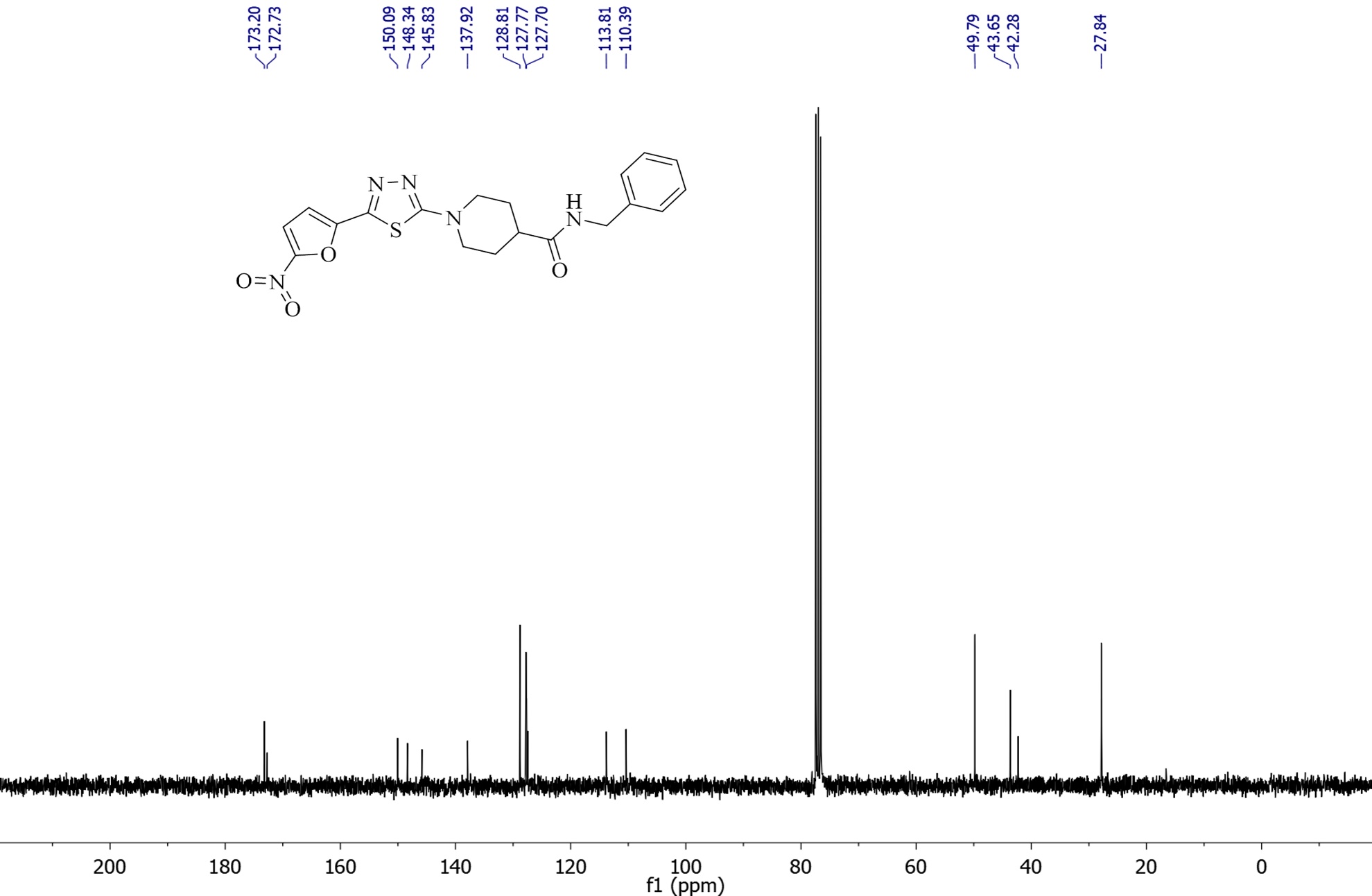
^13^C NMR spectrum of *N*-benzyl-1-(5-(5-nitrofuran-2-yl)-1,3,4-thiadiazol-2-yl)piperidine-4-carboxamide (**9**)


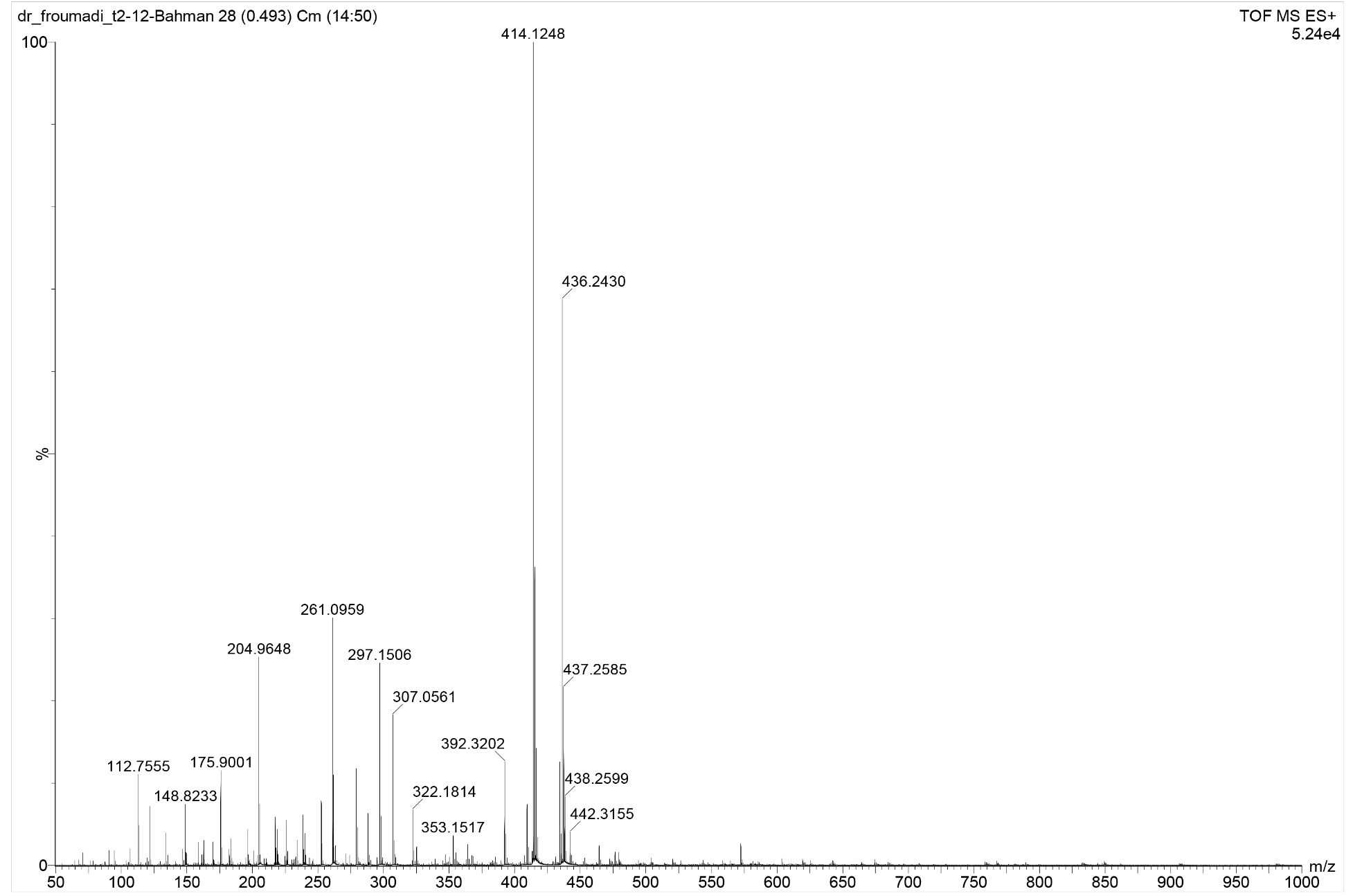
HRMS spectrum of *N*-benzyl-1-(5-(5-nitrofuran-2-yl)-1,3,4-thiadiazol-2-yl)piperidine-4-carboxamide (**9**)

HPLC spectrum of N-benzyl-1-(5-(5-nitrofuran-2-yl)-1,3,4-thiadiazol-2-yl)piperidine-4-carboxamide (**9**)


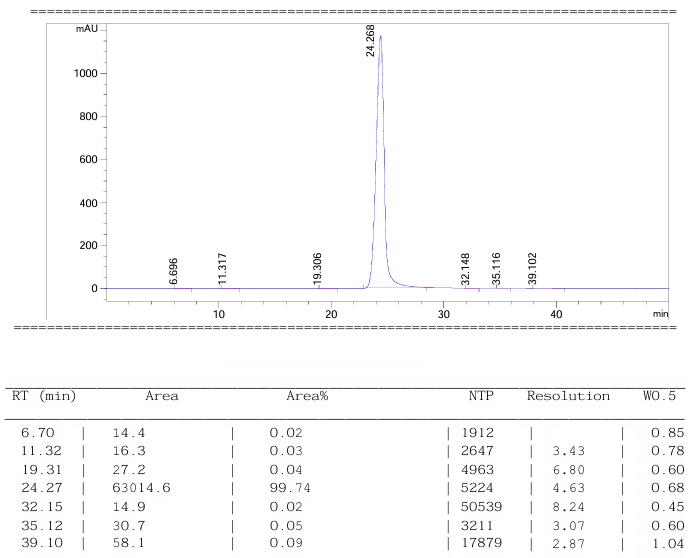


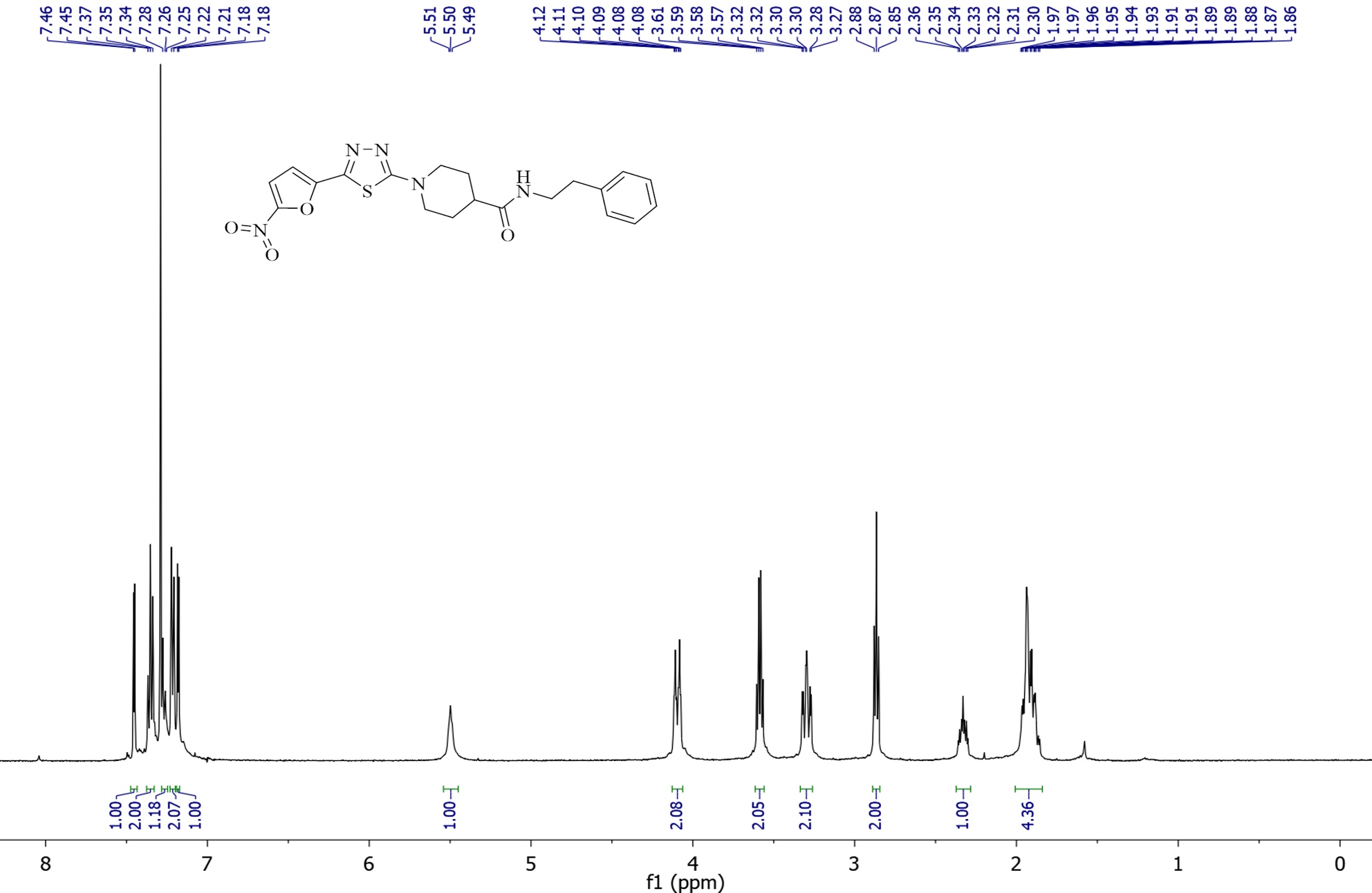
^1^H NMR spectrum of 1-(5-(5-nitrofuran-2-yl)-1,3,4-thiadiazol-2-yl)-*N*-phenethylpiperidine-4-carboxamide (**10**)


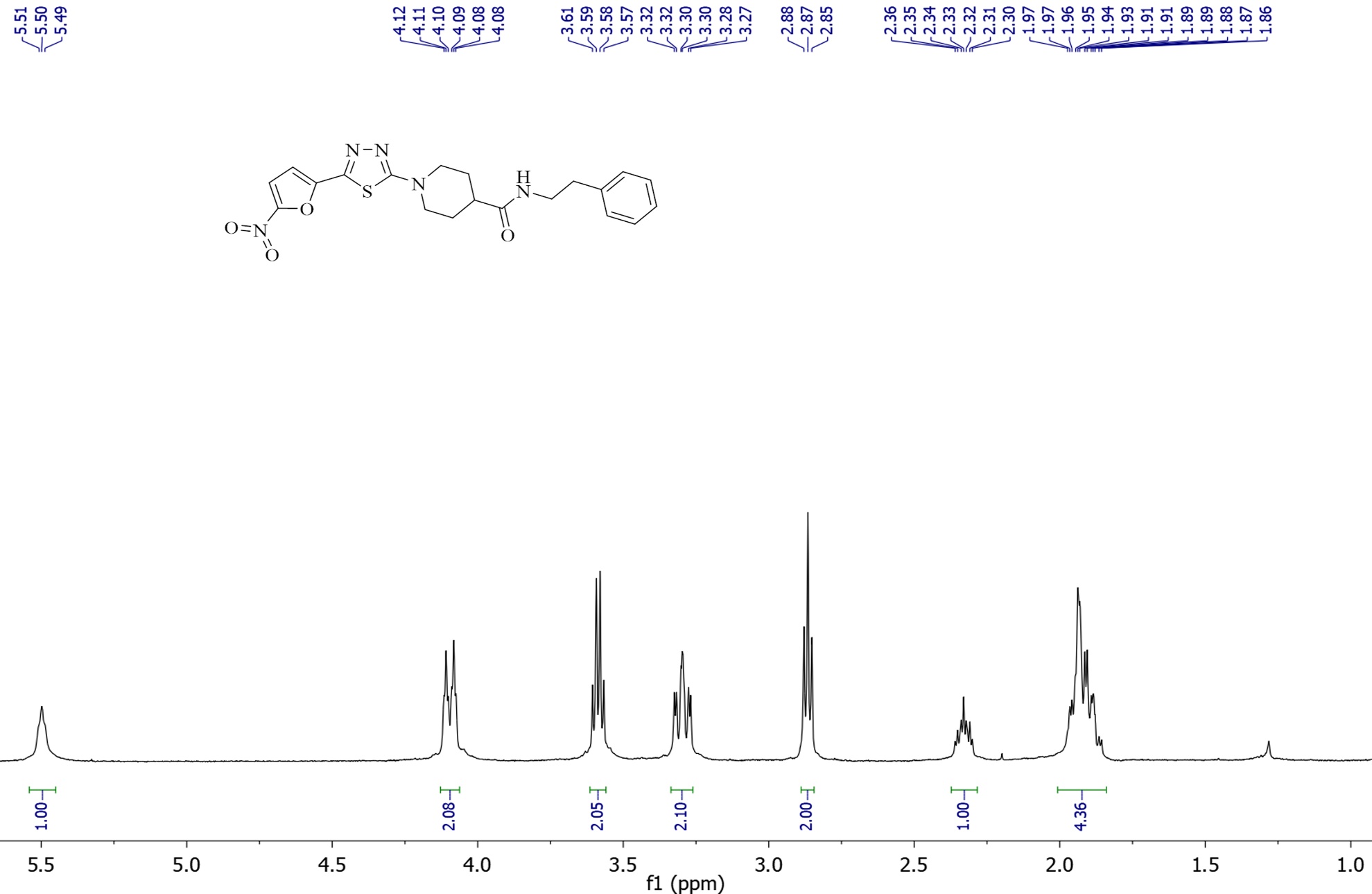
Expanded ^1^H NMR spectrum of 1-(5-(5-nitrofuran-2-yl)-1,3,4-thiadiazol-2-yl)-*N*-phenethylpiperidine-4-carboxamide (**10**)


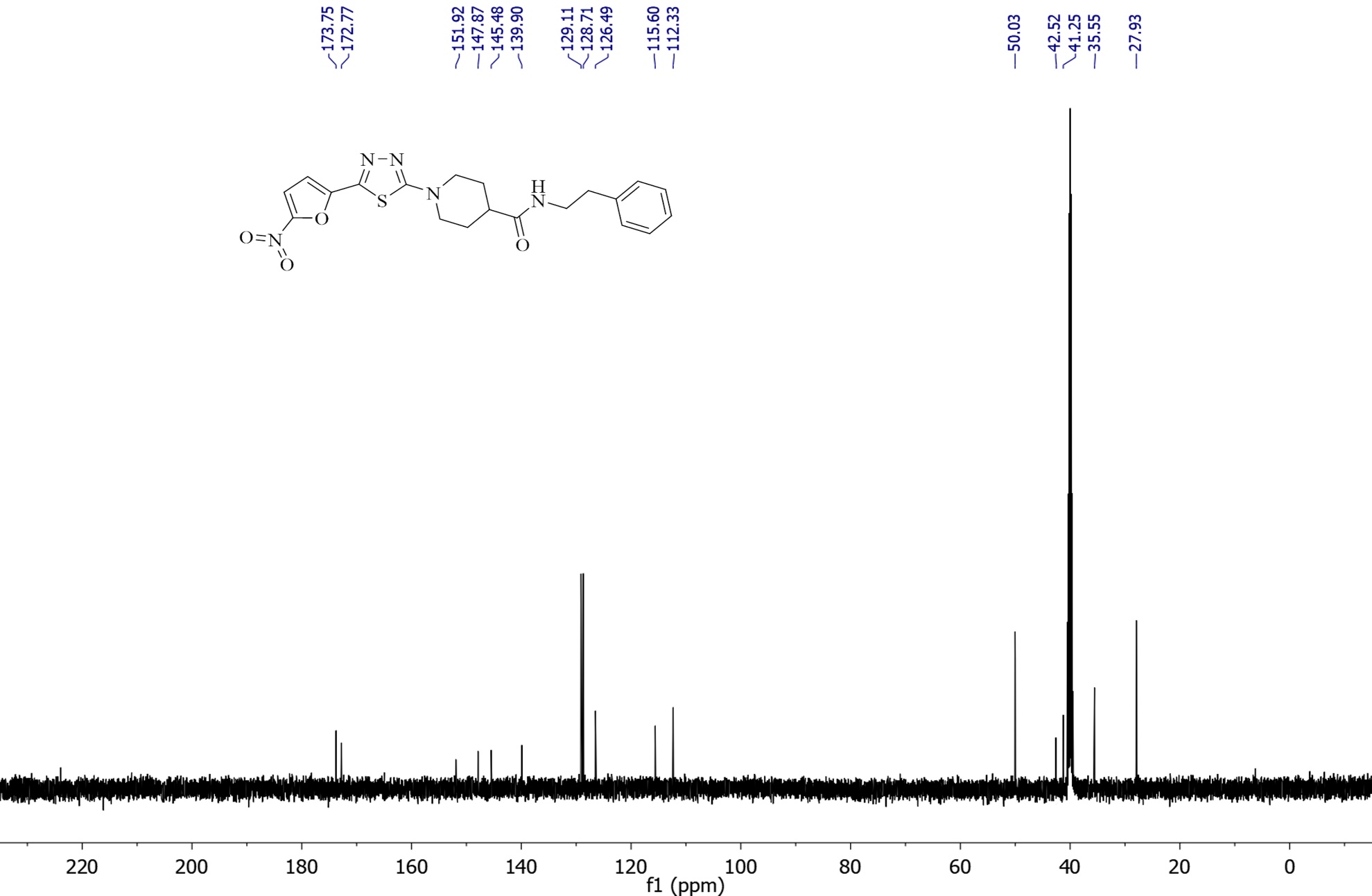
^13^C NMR spectrum of 1-(5-(5-nitrofuran-2-yl)-1,3,4-thiadiazol-2-yl)-*N*-phenethylpiperidine-4-carboxamide (**10**)


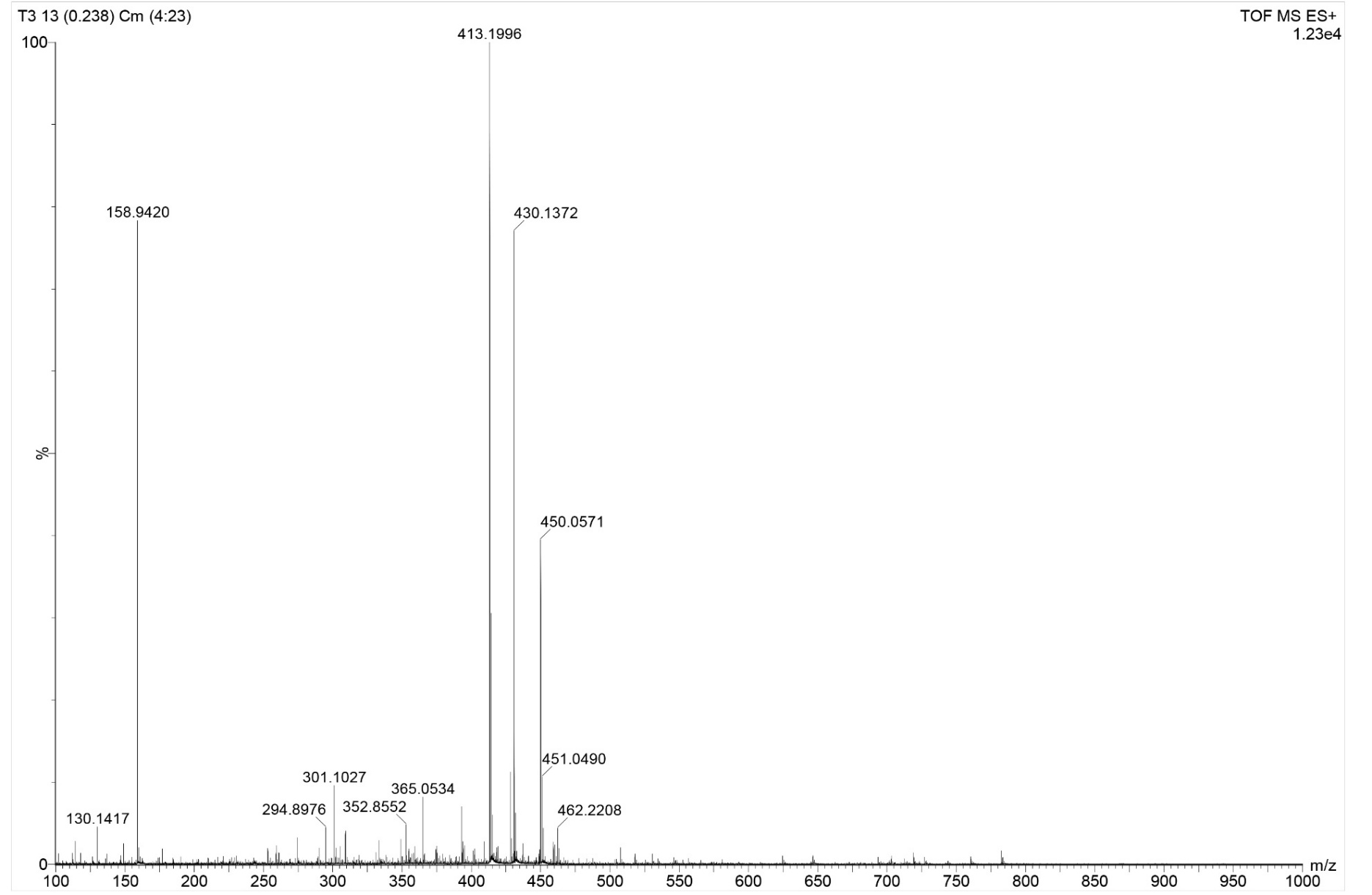
HRMS spectrum of 1-(5-(5-nitrofuran-2-yl)-1,3,4-thiadiazol-2-yl)-*N*-phenethylpiperidine-4-carboxamide (**10**)

HPLC spectrum of 1-(5-(5-nitrofuran-2-yl)-1,3,4-thiadiazol-2-yl)-N-phenethylpiperidine-4-carboxamide (**10**)


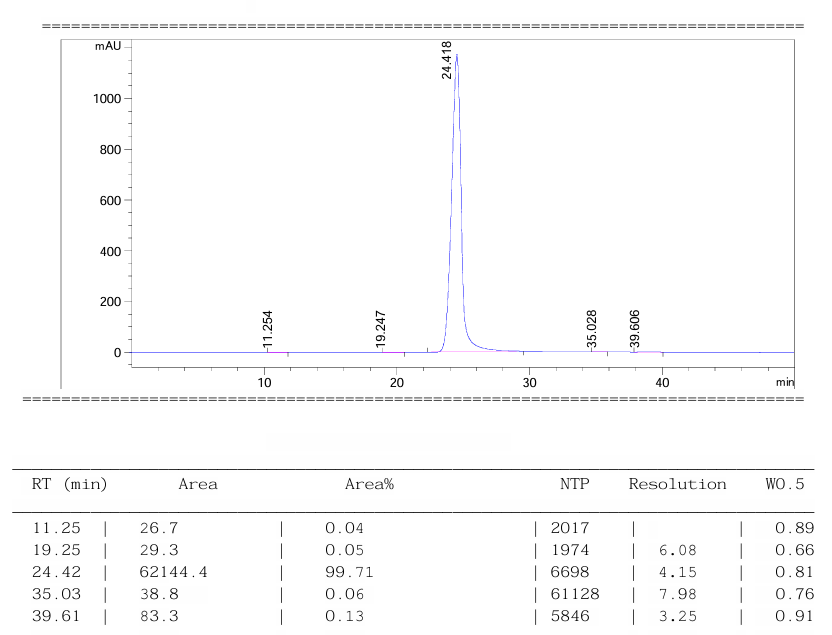


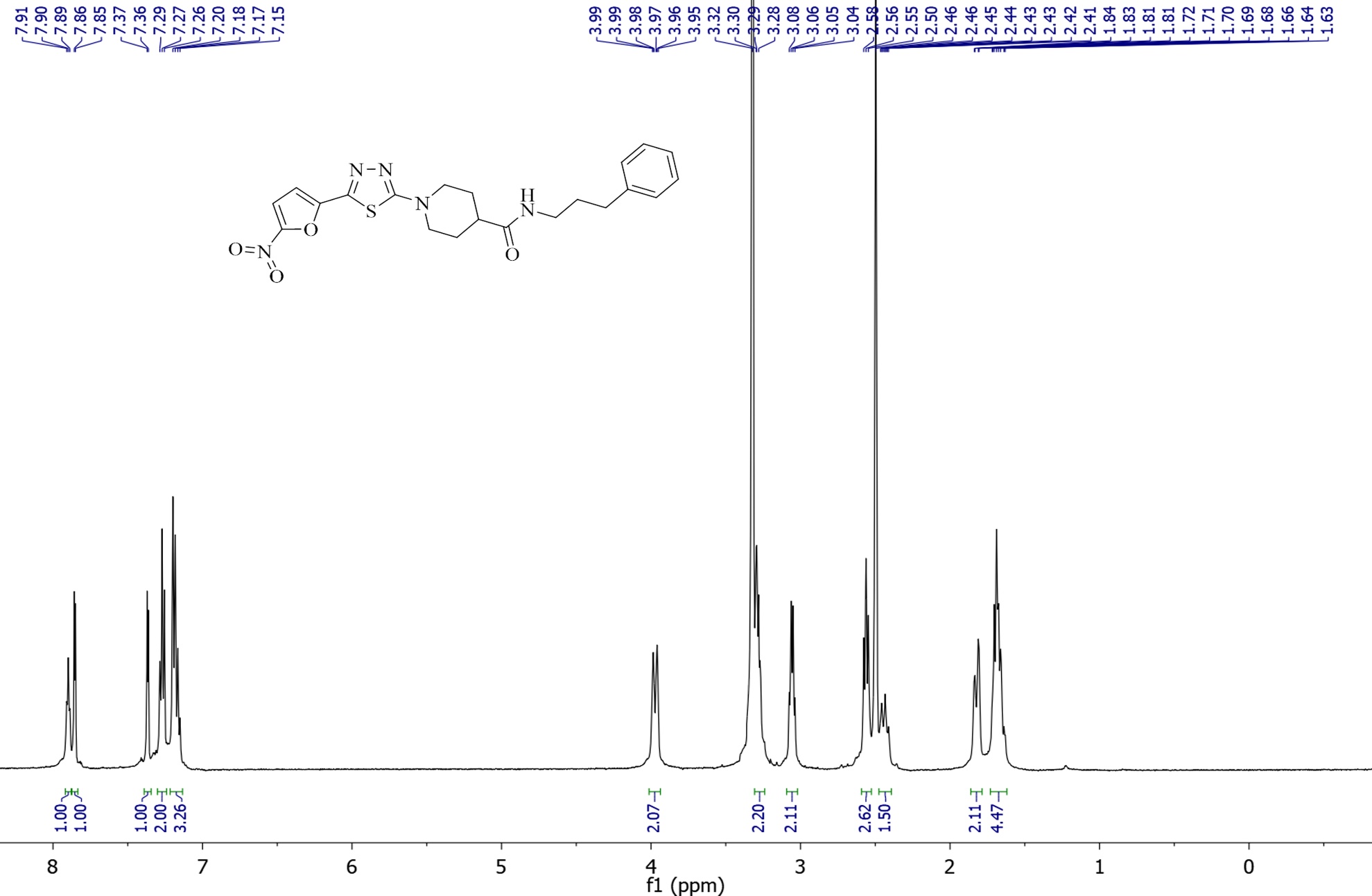
^1^H NMR spectrum of 1-(5-(5-nitrofuran-2-yl)-1,3,4-thiadiazol-2-yl)-*N*-(3-phenylpropyl)piperidine-4-carboxamide (**11**)


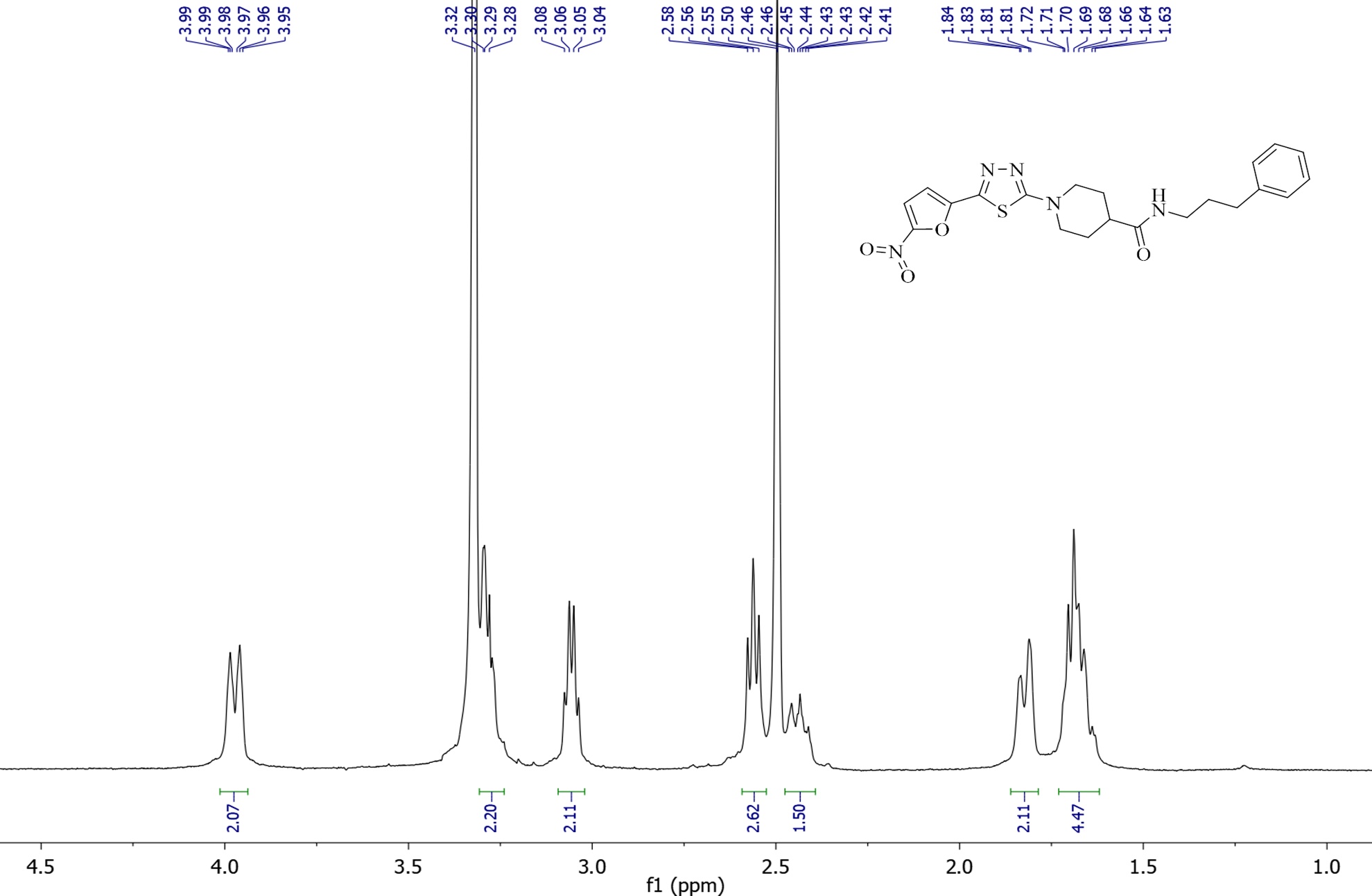
Expanded ^1^H NMR spectrum of 1-(5-(5-nitrofuran-2-yl)-1,3,4-thiadiazol-2-yl)-*N*-(3-phenylpropyl)piperidine-4-carboxamide (**11**)

^
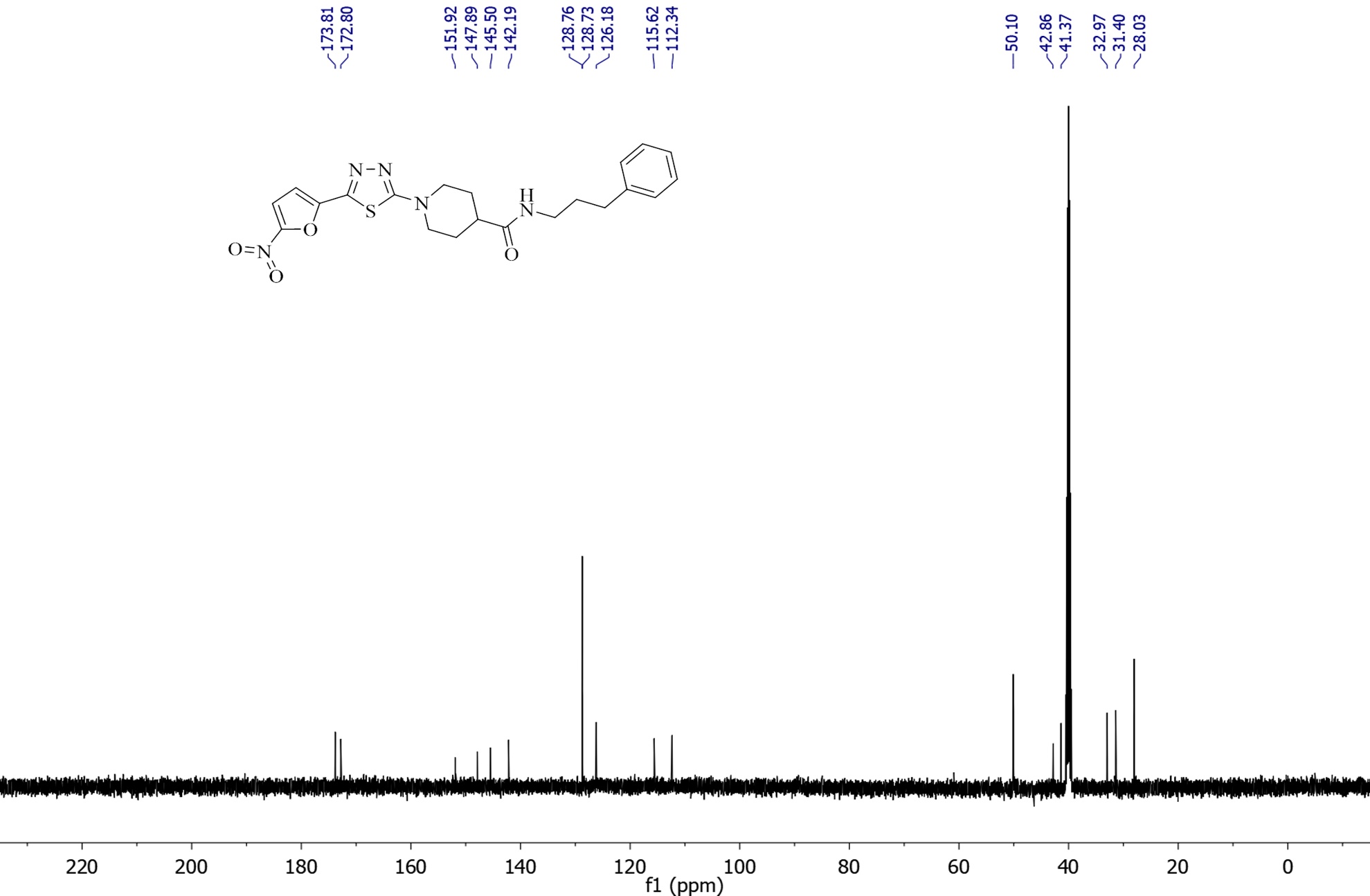
13^C NMR spectrum of 1-(5-(5-nitrofuran-2-yl)-1,3,4-thiadiazol-2-yl)-*N*-(3-phenylpropyl)piperidine-4-carboxamide (**11**)

HRMS spectrum of 1-(5-(5-nitrofuran-2-yl)-1,3,4-thiadiazol-2-yl)-*N*-(3-phenylpropyl)piperidine-4-carboxamide (**11**)


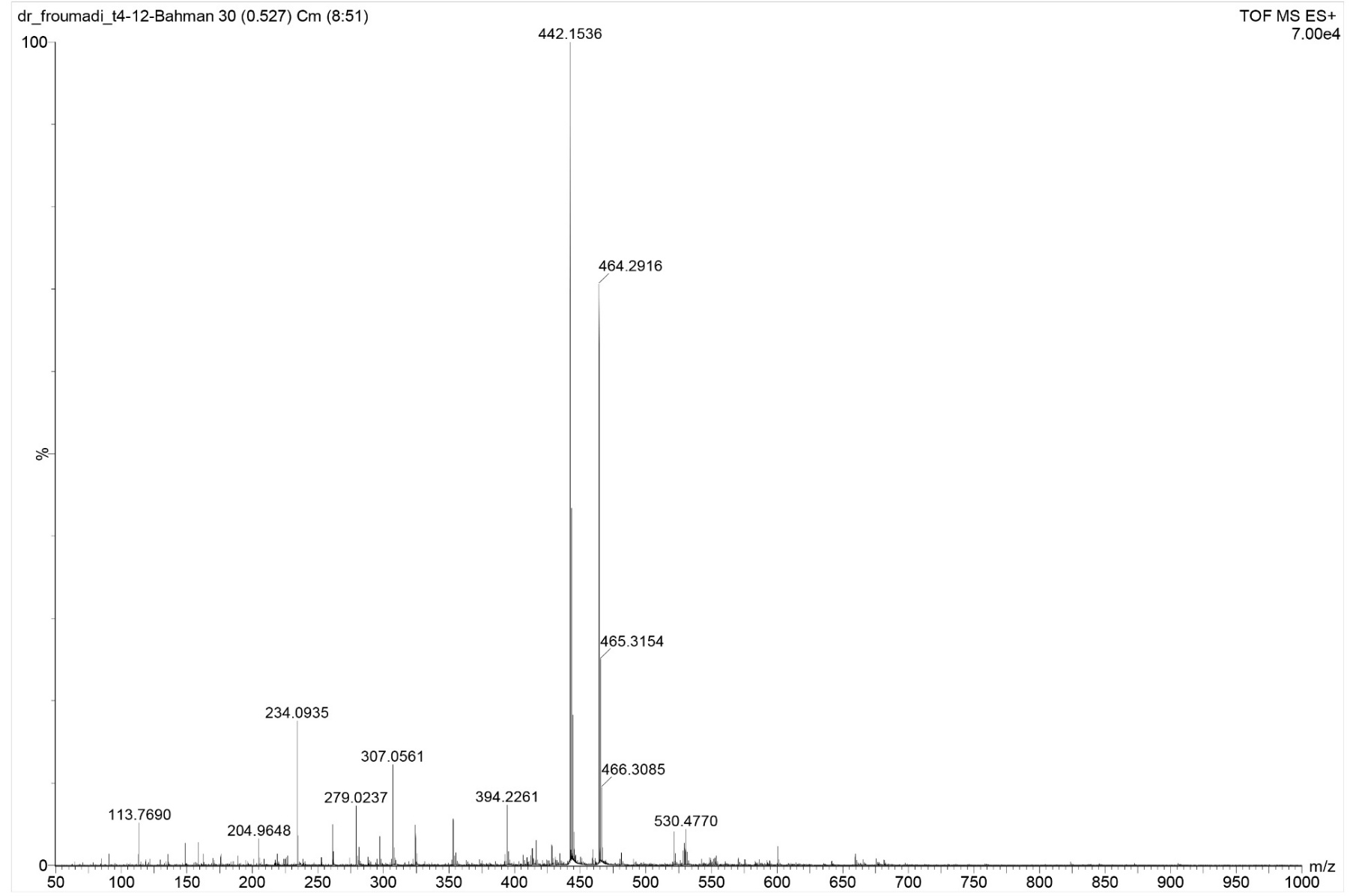


HPLC spectrum of 1-(5-(5-nitrofuran-2-yl)-1,3,4-thiadiazol-2-yl)-N-(3-phenylpropyl)piperidine-4-carboxamide (**11**)


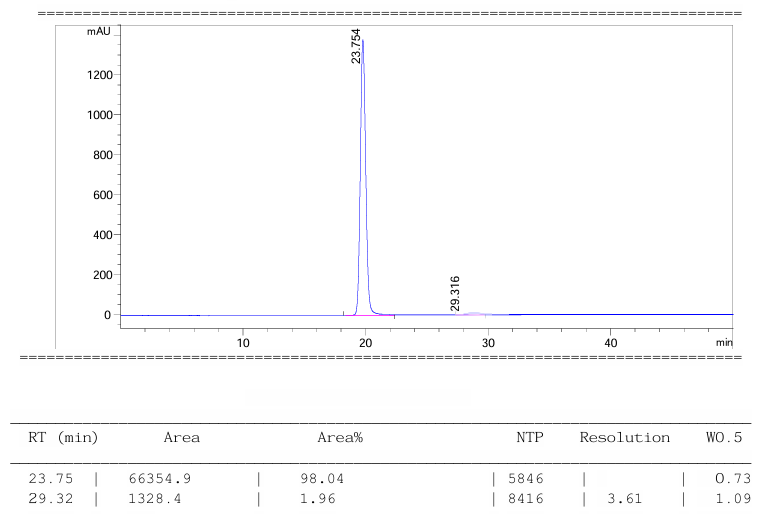


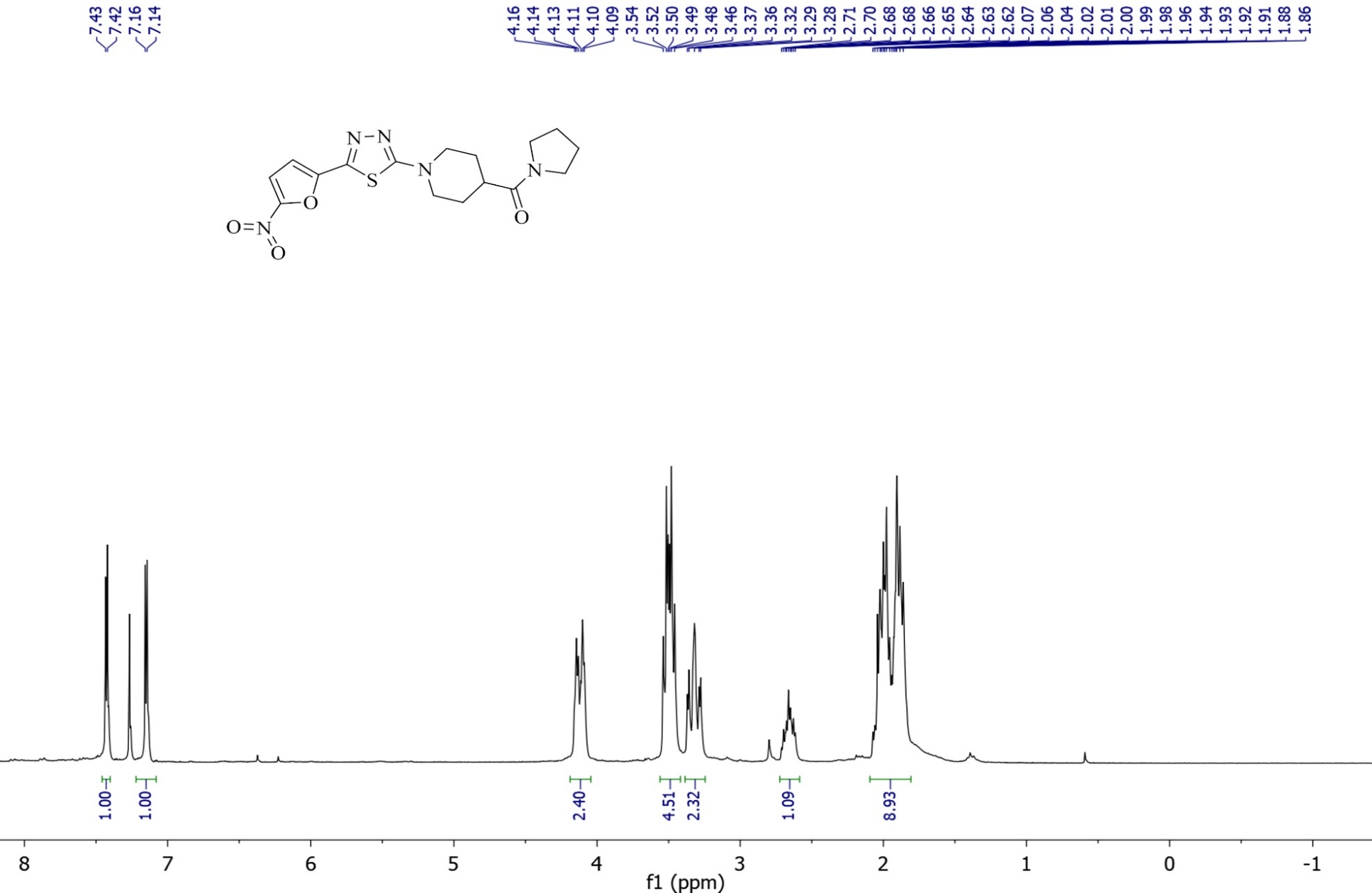
^1^H NMR spectrum of (1-(5-(5-nitrofuran-2-yl)-1,3,4-thiadiazol-2-yl)piperidin-4-yl)(pyrrolidin-1-yl)methanone (**12**)


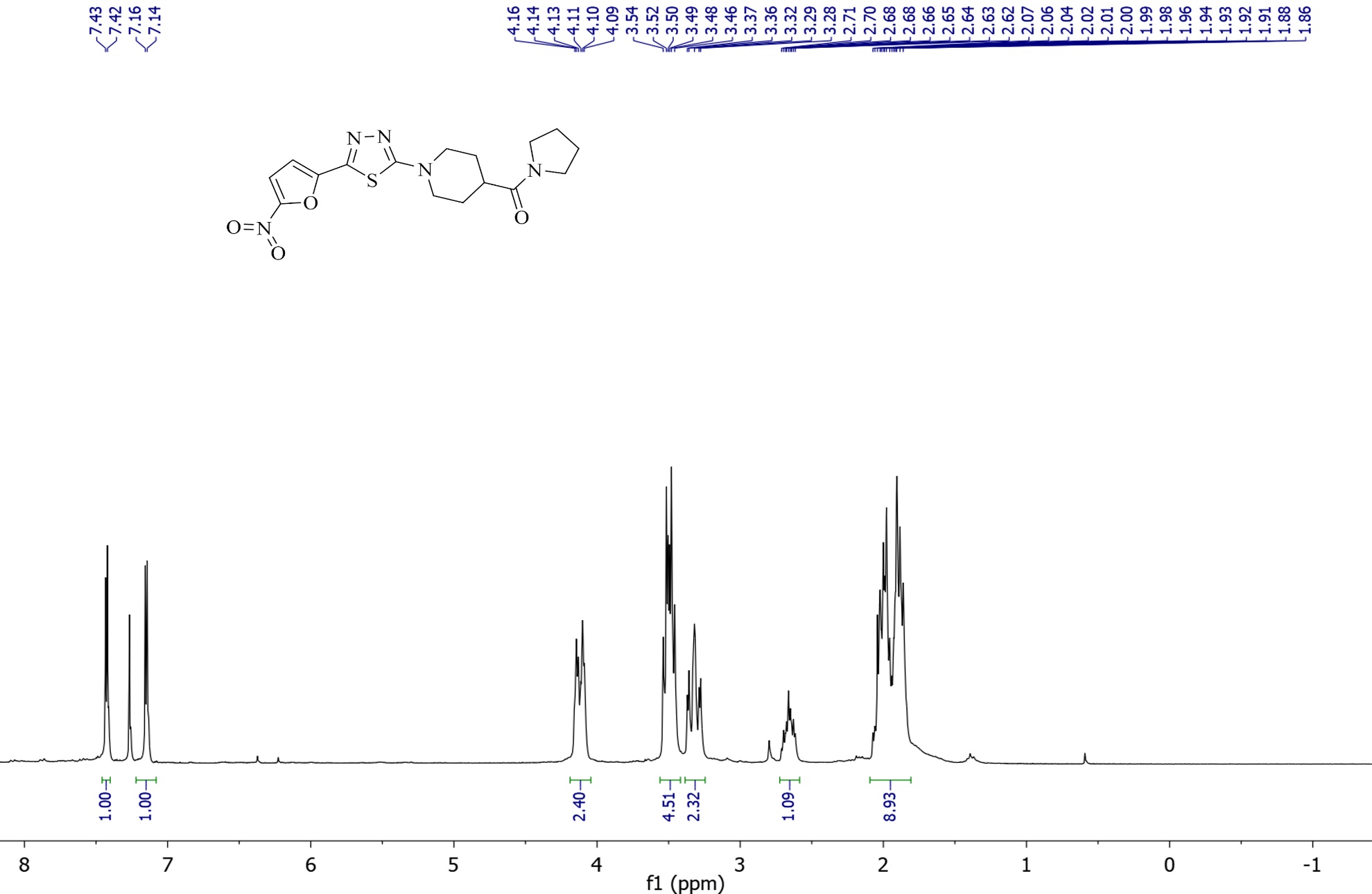
Expanded ^1^H NMR spectrum of (1-(5-(5-nitrofuran-2-yl)-1,3,4-thiadiazol-2-yl)piperidin-4-yl)(pyrrolidin-1-yl)methanone (**12**)


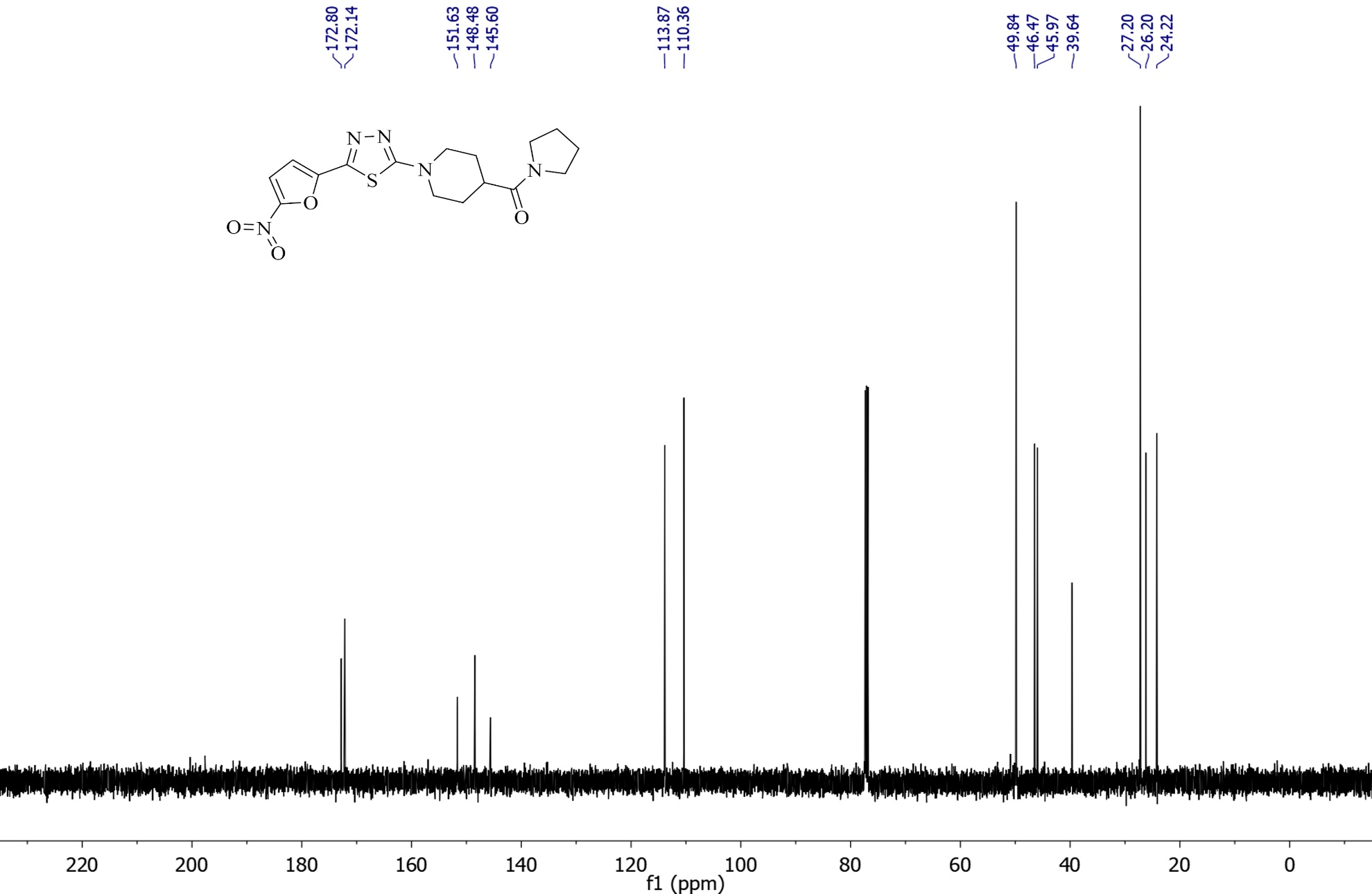
^13^C NMR spectrum of (1-(5-(5-nitrofuran-2-yl)-1,3,4-thiadiazol-2-yl)piperidin-4-yl)(pyrrolidin-1-yl)methanone (**12**)


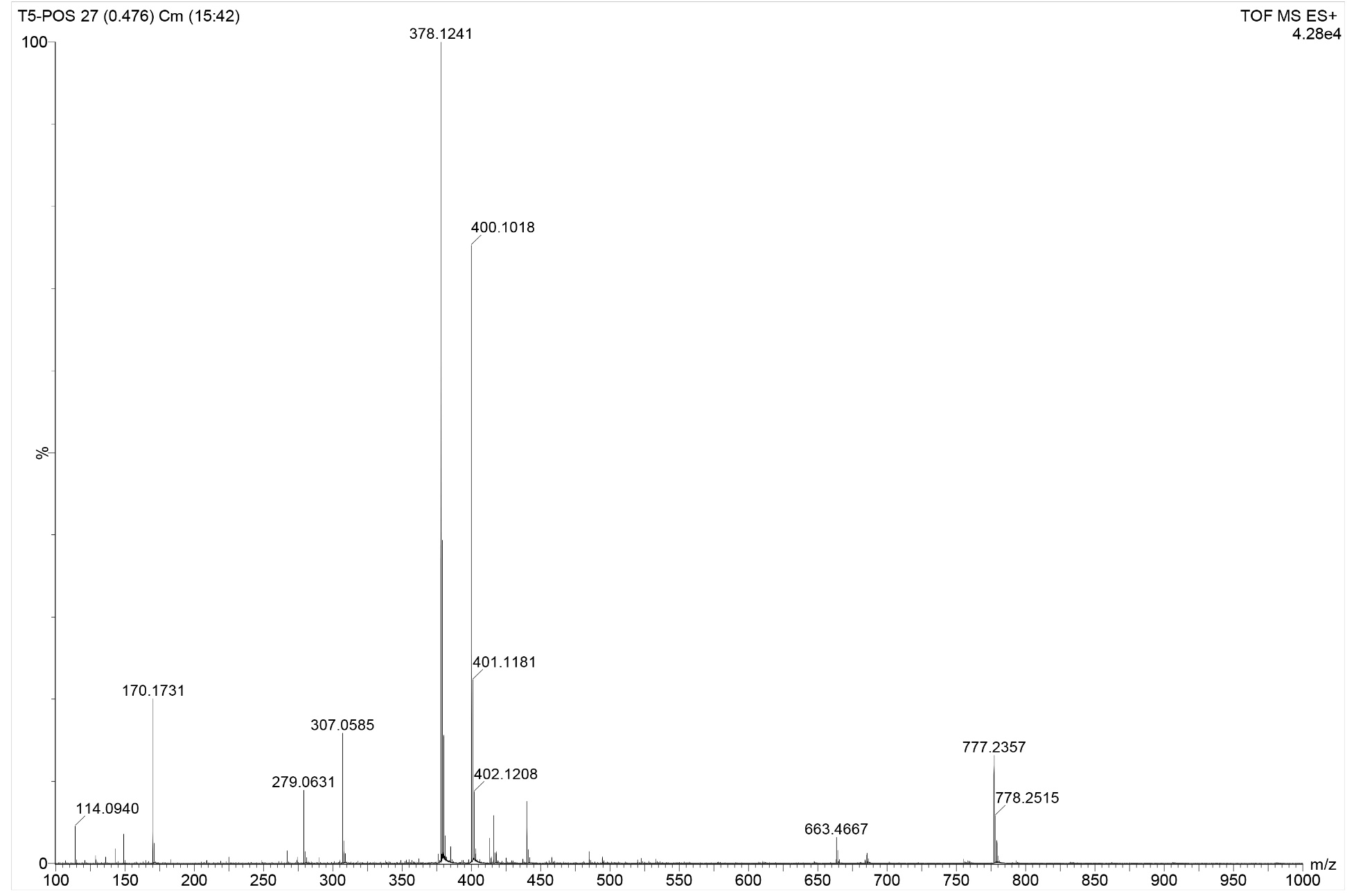
HRMS spectrum of (1-(5-(5-nitrofuran-2-yl)-1,3,4-thiadiazol-2-yl)piperidin-4-yl)(pyrrolidin-1-yl)methanone (**12**)

HPLC spectrum of (1-(5-(5-nitrofuran-2-yl)-1,3,4-thiadiazol-2-yl)piperidin-4-yl)(pyrrolidin-1-yl)methanone (**12**)

**
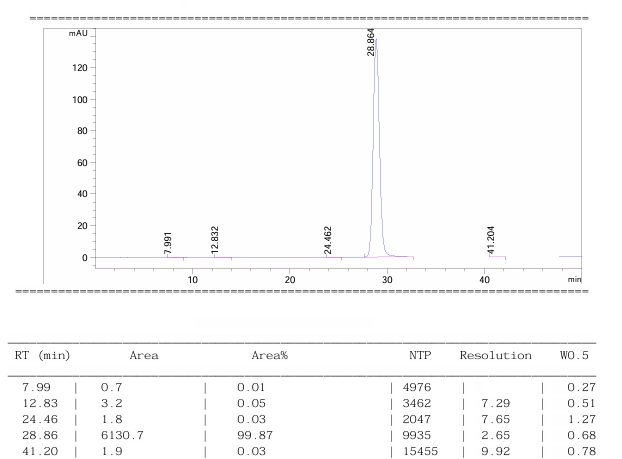
**


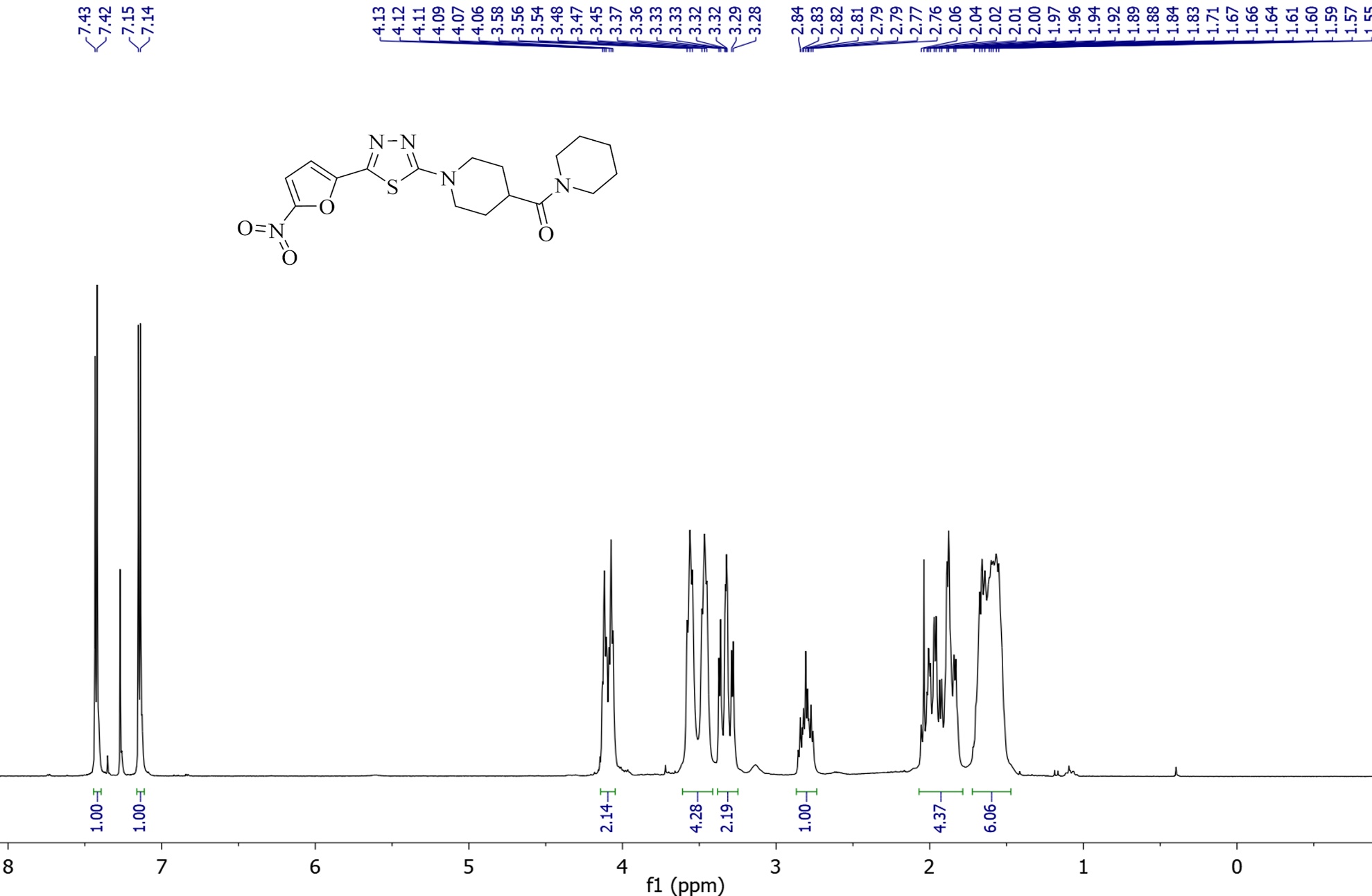
^1^H NMR spectrum of (1-(5-(5-nitrofuran-2-yl)-1,3,4-thiadiazol-2-yl)piperidin-4-yl)(piperidin-1-yl)methanone (**13**)


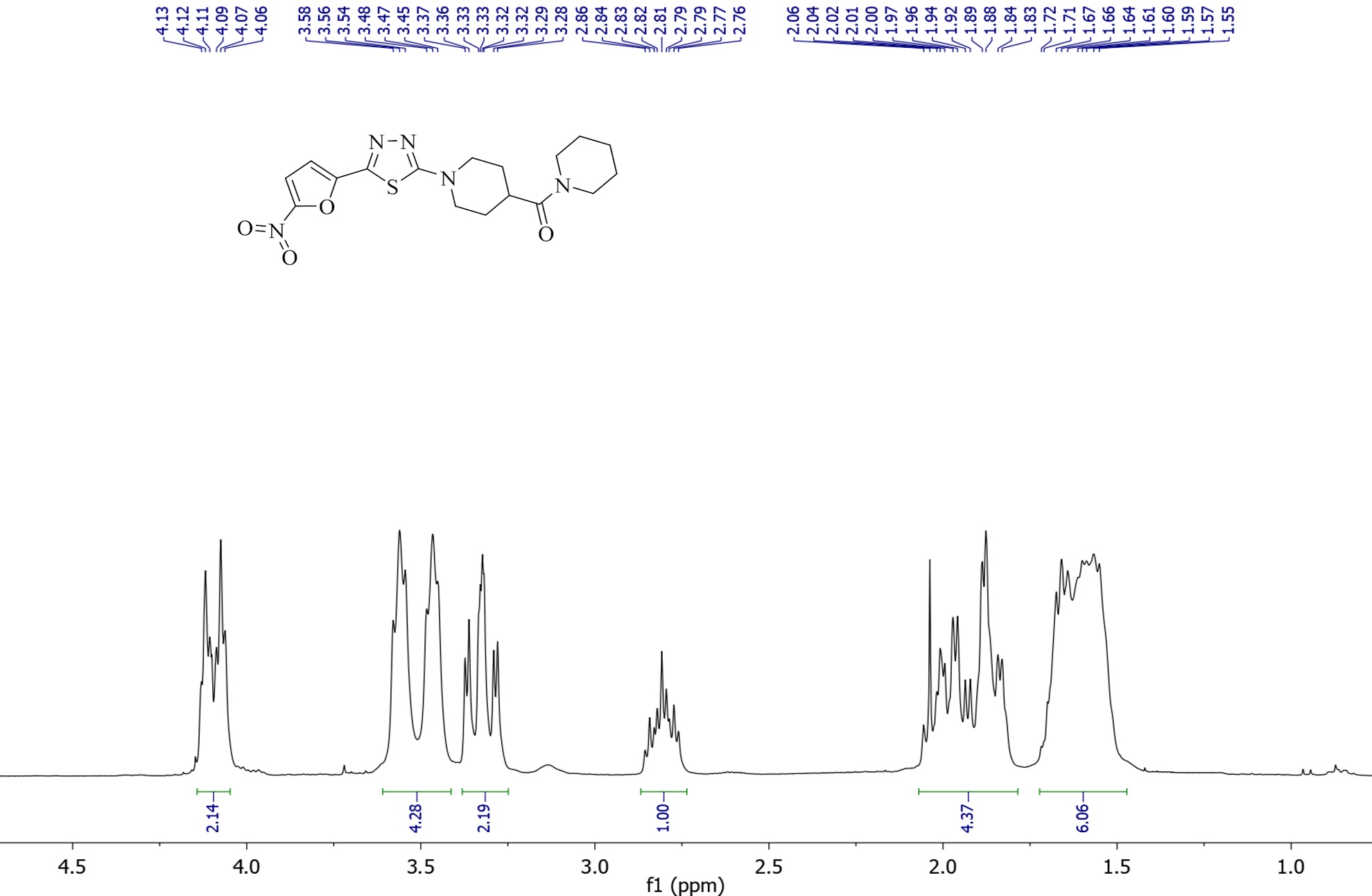
Expanded ^1^H NMR spectrum of (1-(5-(5-nitrofuran-2-yl)-1,3,4-thiadiazol-2-yl)piperidin-4-yl)(piperidin-1-yl)methanone (**13**)


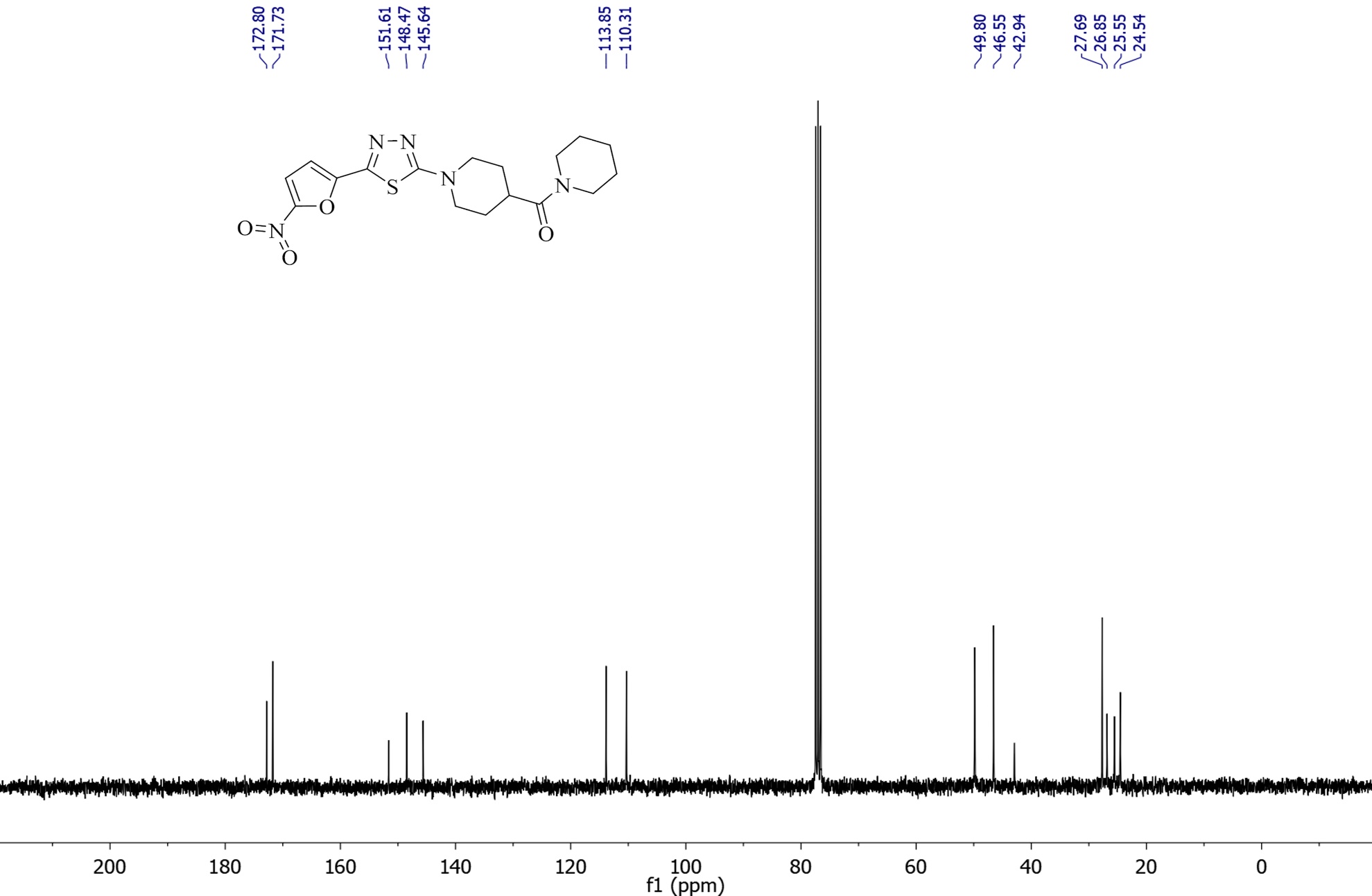
^13^C NMR spectrum of (1-(5-(5-nitrofuran-2-yl)-1,3,4-thiadiazol-2-yl)piperidin-4-yl)(piperidin-1-yl)methanone (**13**)


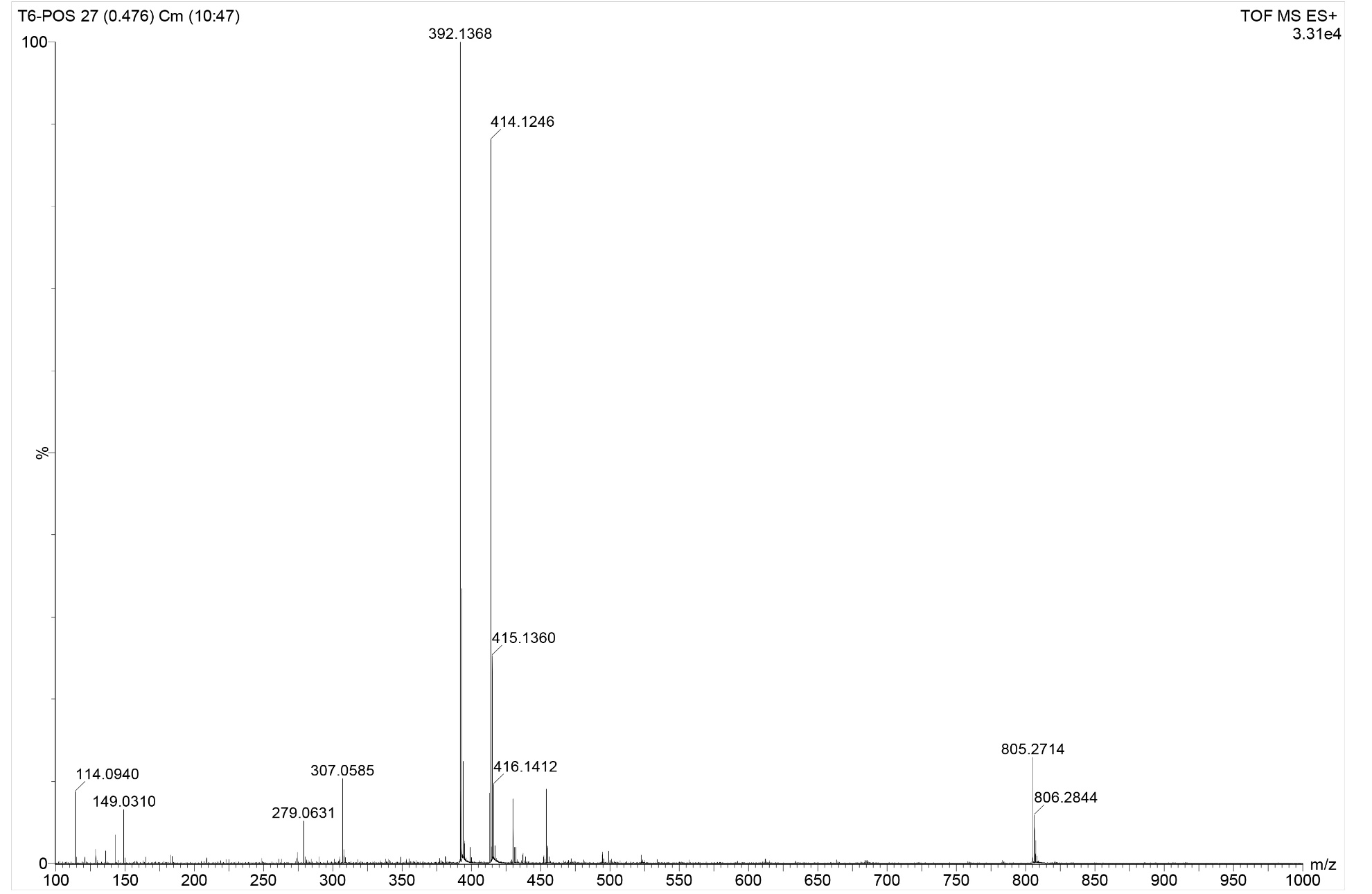
HRMS spectrum of (1-(5-(5-nitrofuran-2-yl)-1,3,4-thiadiazol-2-yl)piperidin-4-yl)(piperidin-1-yl)methanone (**13**)

HPLC spectrum of (1-(5-(5-nitrofuran-2-yl)-1,3,4-thiadiazol-2-yl)piperidin-4-yl)(piperidin-1-yl)methanone (**13**)


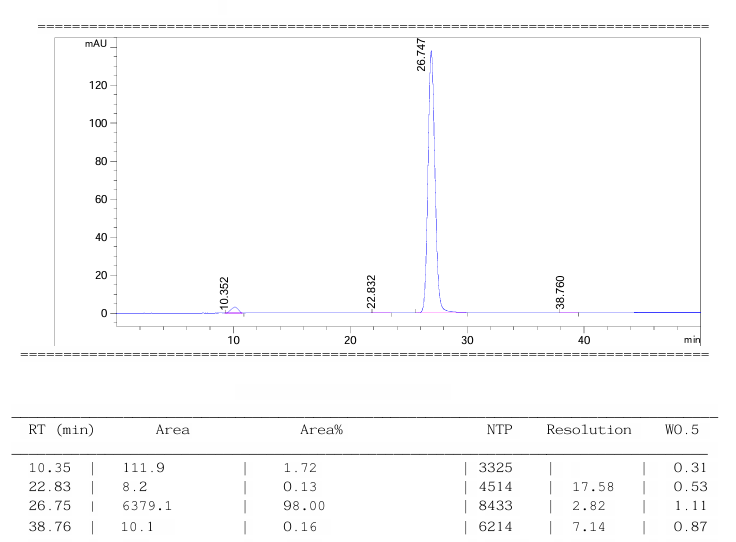


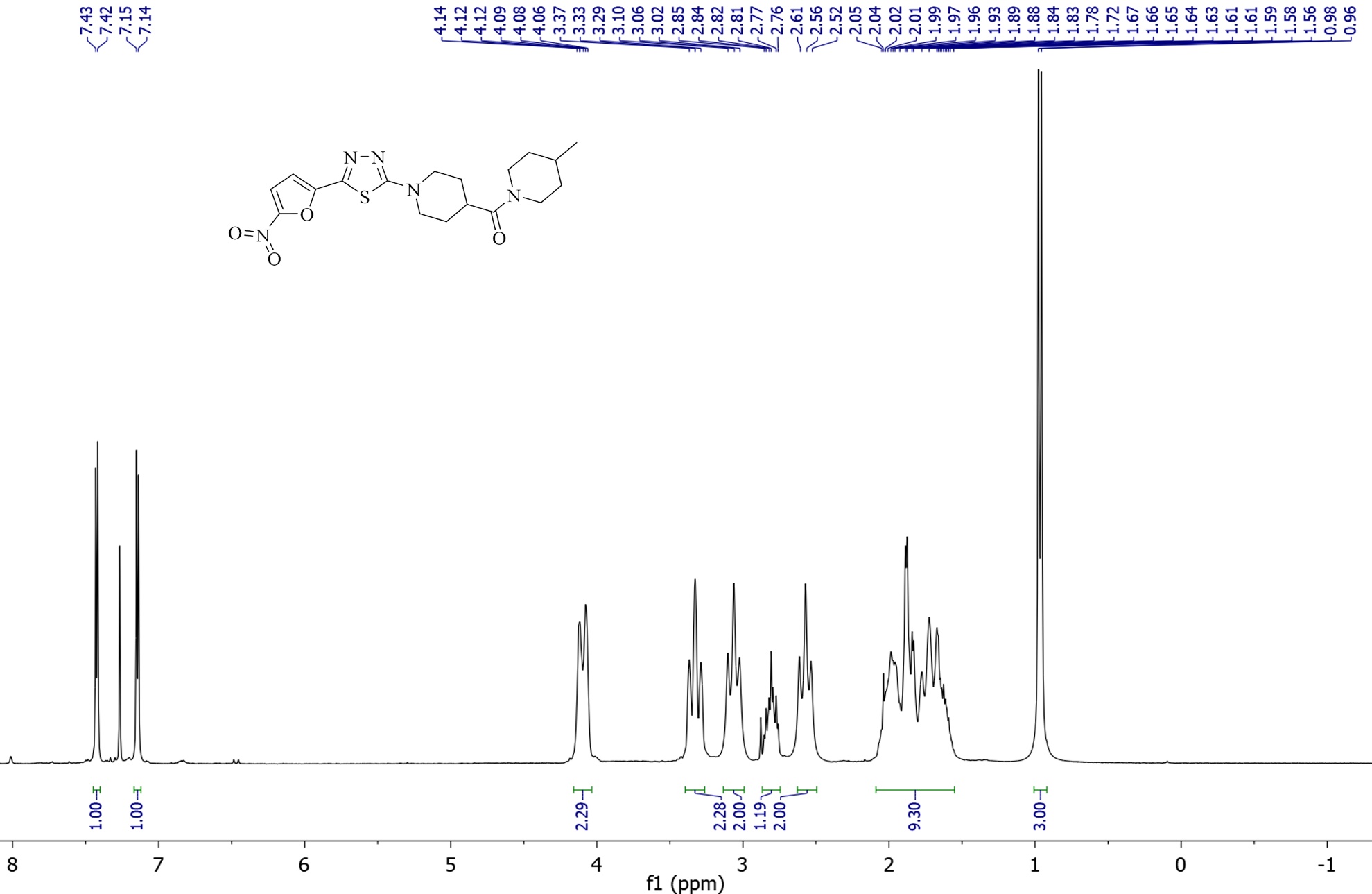
^1^H NMR spectrum of (4-methylpiperidin-1-yl)(1-(5-(5-nitrofuran-2-yl)-1,3,4-thiadiazol-2-yl)piperidin-4-yl)methanone (**14**)


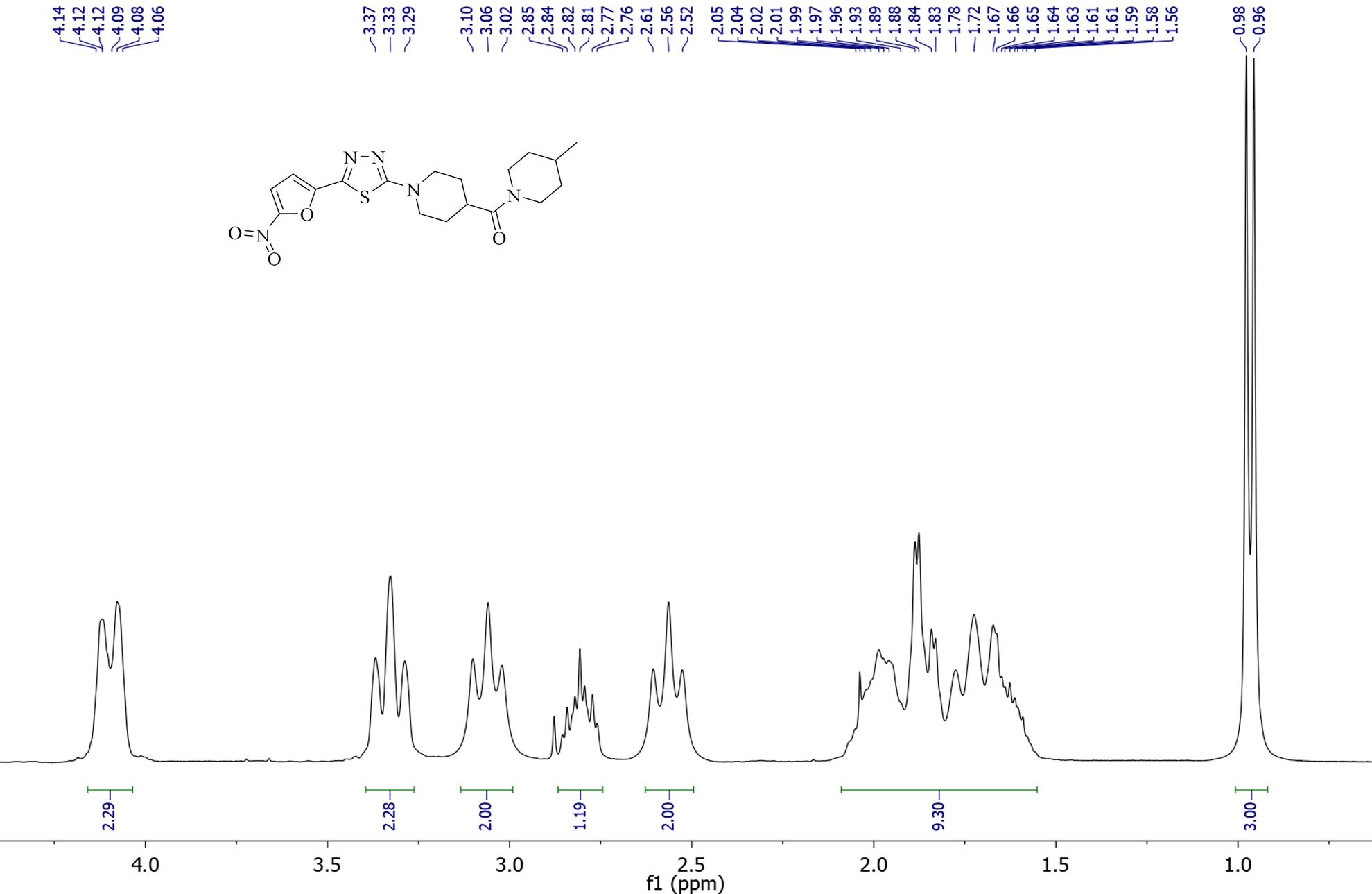
Expanded ^1^H NMR spectrum of (4-methylpiperidin-1-yl)(1-(5-(5-nitrofuran-2-yl)-1,3,4-thiadiazol-2-yl)piperidin-4-yl)methanone (**14**)


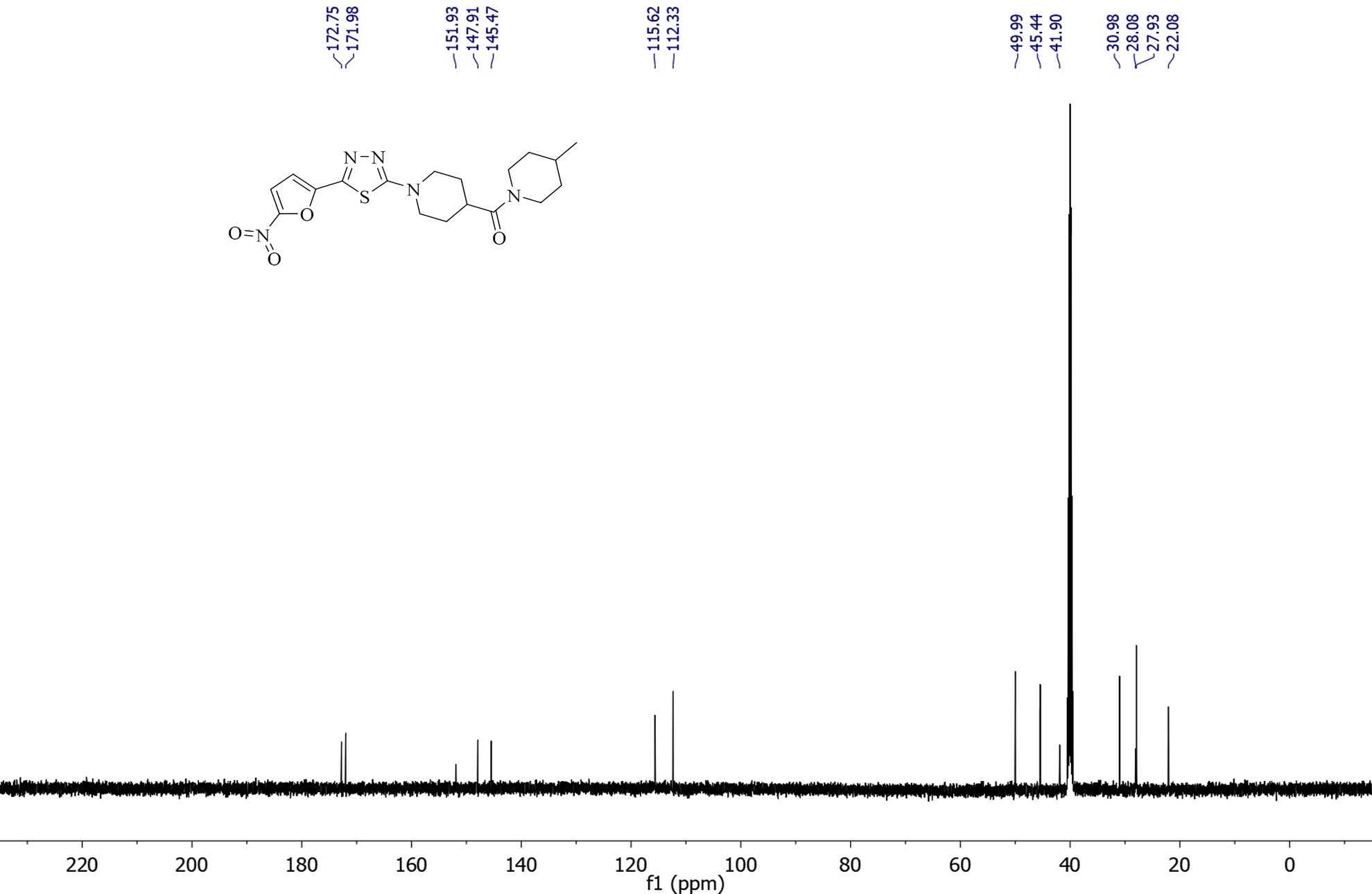
^13^C NMR spectrum of (4-methylpiperidin-1-yl)(1-(5-(5-nitrofuran-2-yl)-1,3,4-thiadiazol-2-yl)piperidin-4-yl)methanone (**14**)


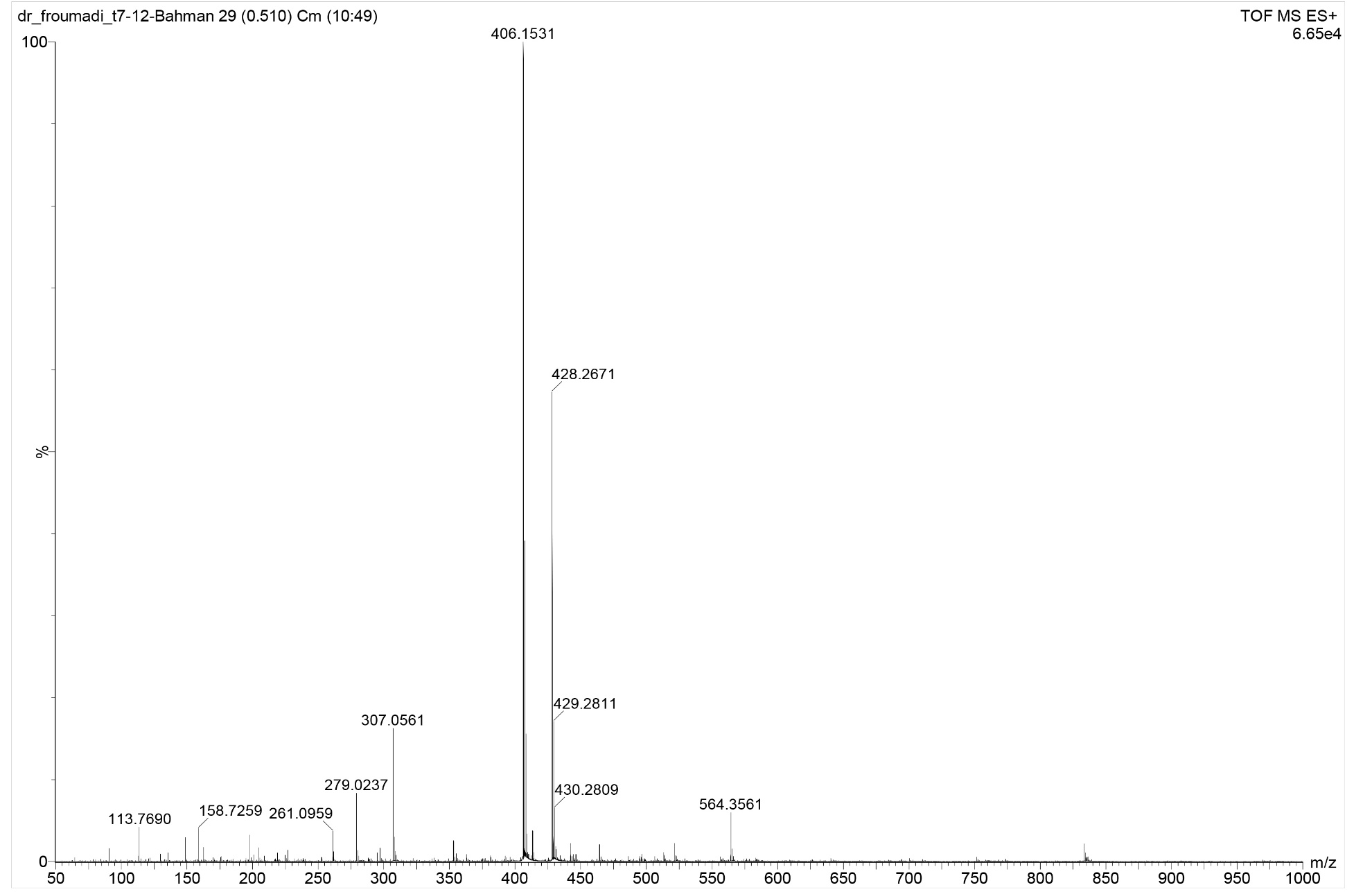
HRMS spectrum of (4-methylpiperidin-1-yl)(1-(5-(5-nitrofuran-2-yl)-1,3,4-thiadiazol-2-yl)piperidin-4-yl)methanone (**14**)

HPLC spectrum of (4-methylpiperidin-1-yl)(1-(5-(5-nitrofuran-2-yl)-1,3,4-thiadiazol-2-yl)piperidin-4-yl)methanone (**14**)


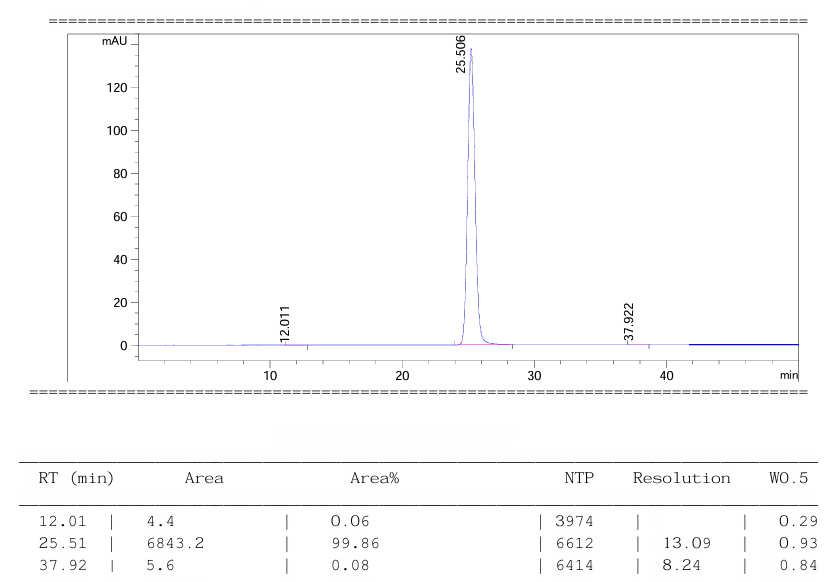


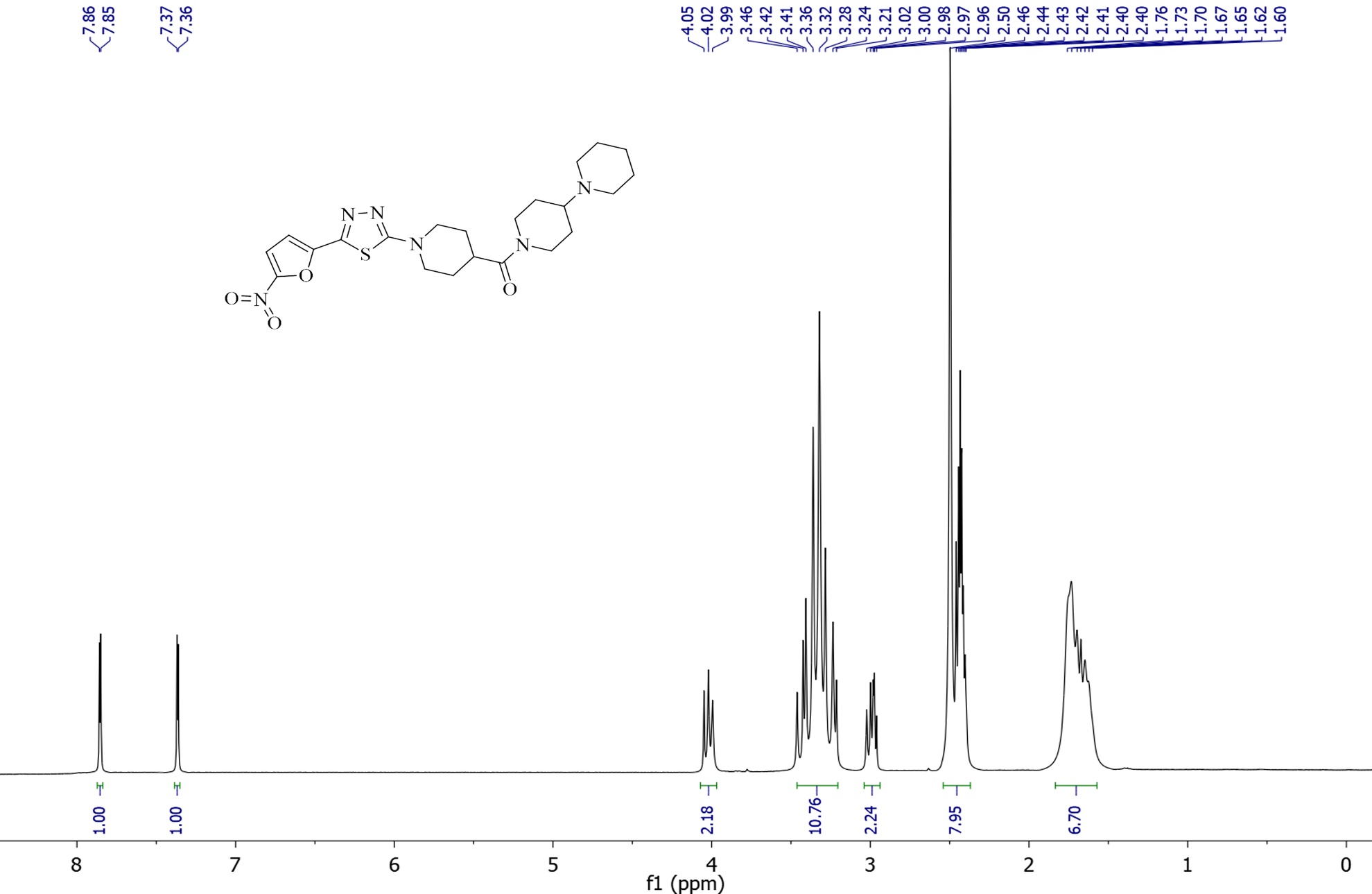
^1^H NMR spectrum of [1,4'-bipiperidin]-1'-yl(1-(5-(5-nitrofuran-2-yl)-1,3,4-thiadiazol-2-yl)piperidin-4-yl)methanone (**15**)


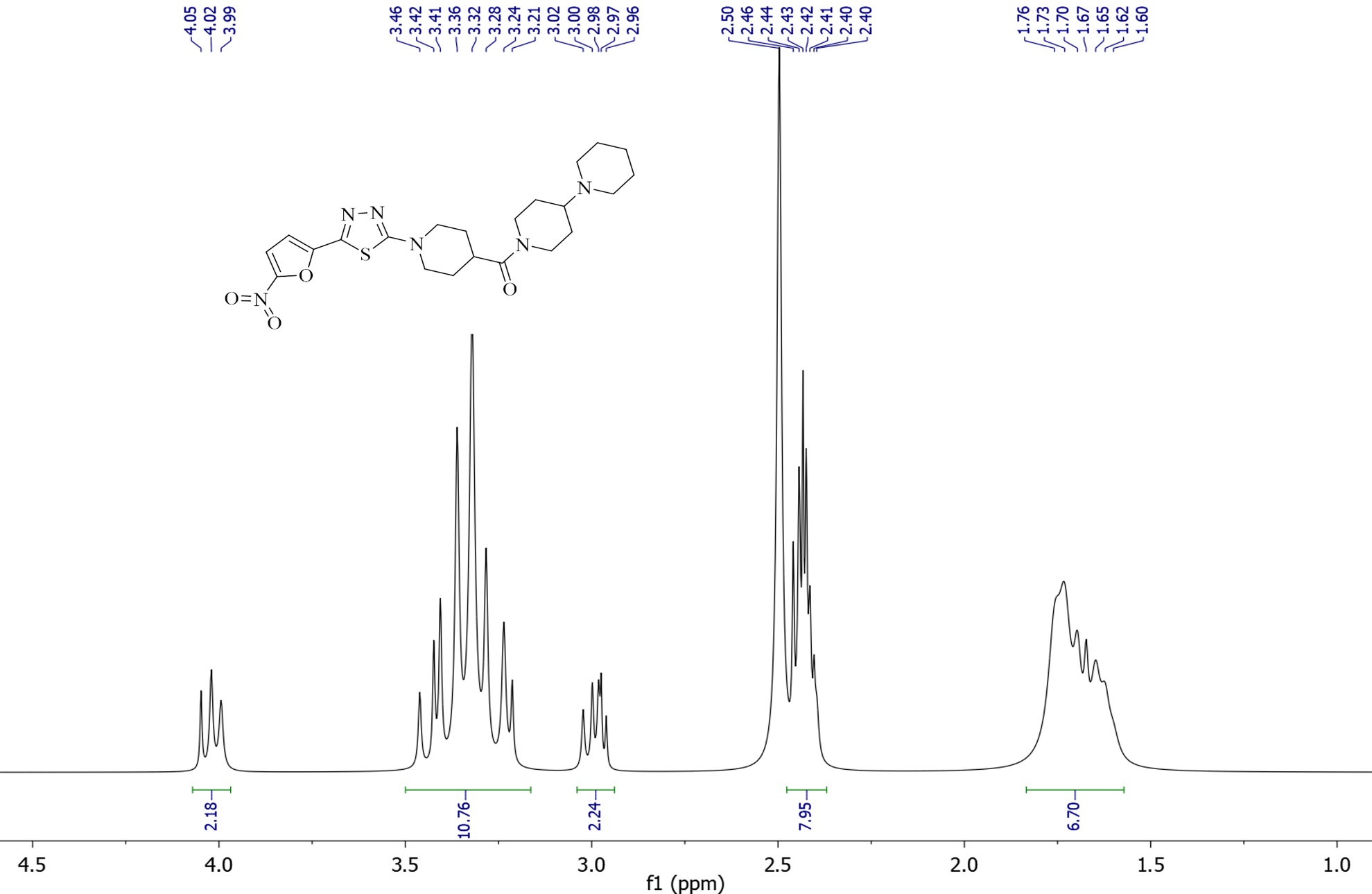
Expanded ^1^H NMR spectrum of [1,4'-bipiperidin]-1'-yl(1-(5-(5-nitrofuran-2-yl)-1,3,4-thiadiazol-2-yl)piperidin-4-yl)methanone (**15**)


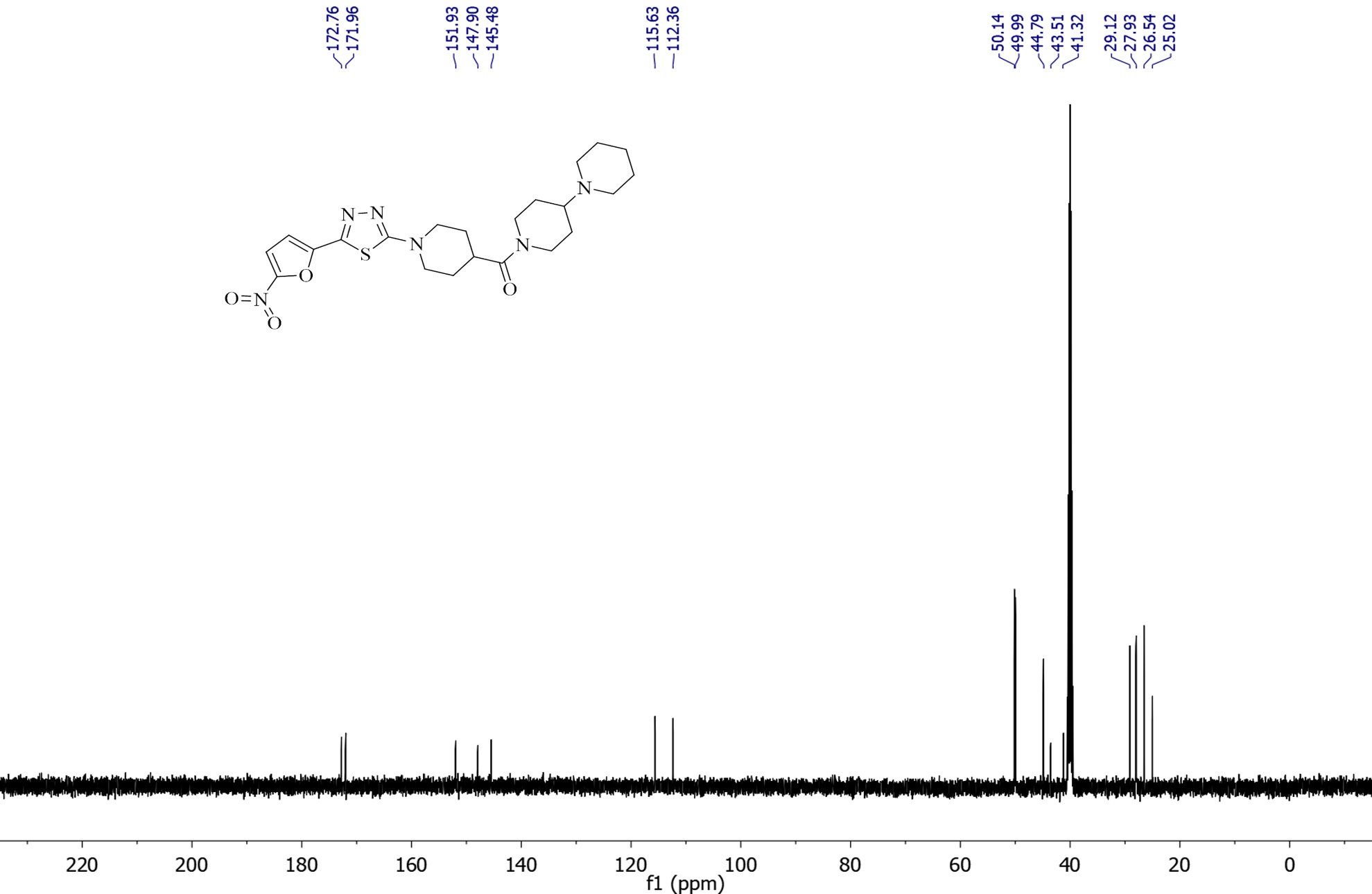
^13^C NMR spectrum of [1,4'-bipiperidin]-1'-yl(1-(5-(5-nitrofuran-2-yl)-1,3,4-thiadiazol-2-yl)piperidin-4-yl)methanone (**15**)


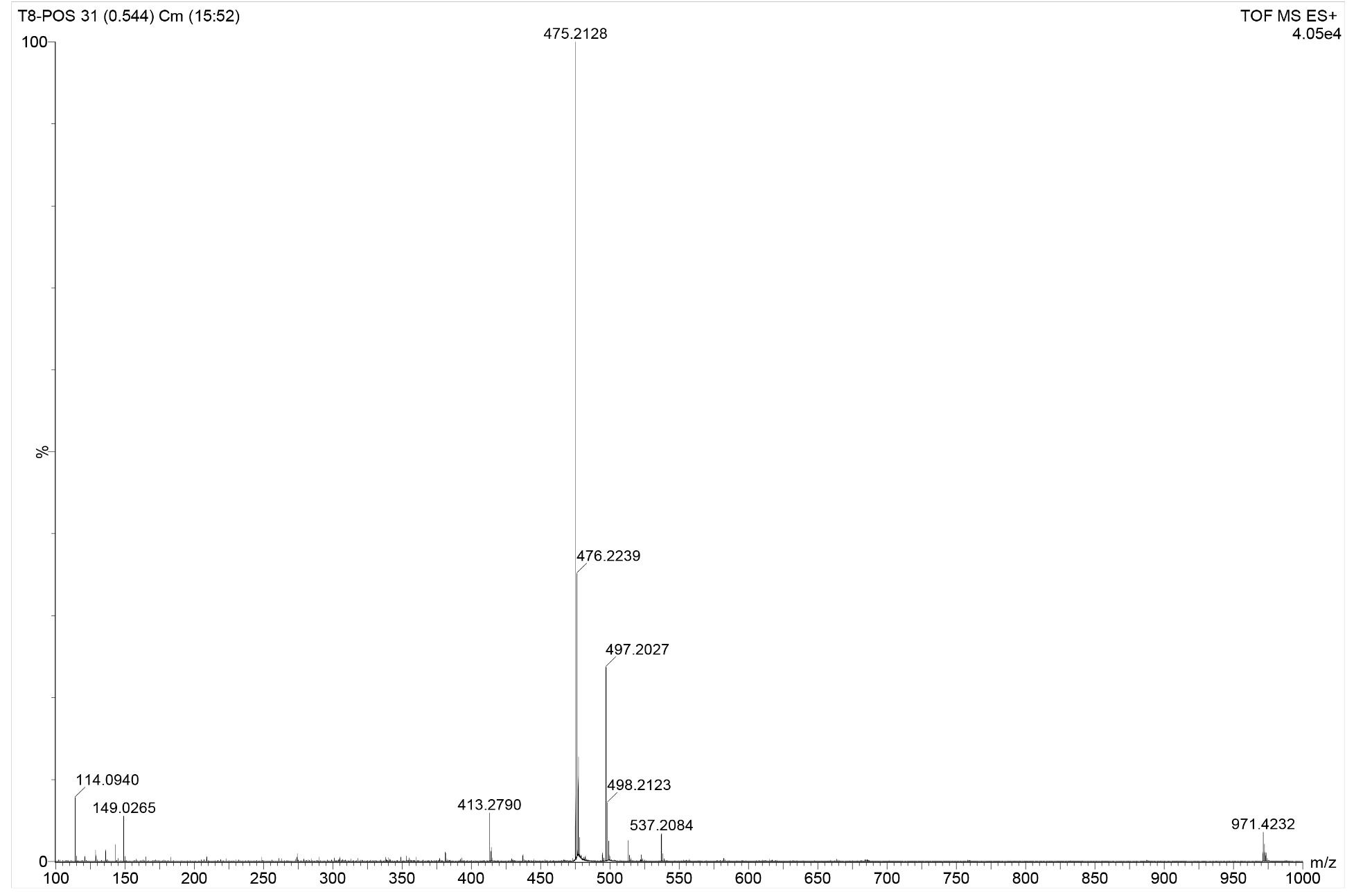
HRMS spectrum of [1,4'-bipiperidin]-1'-yl(1-(5-(5-nitrofuran-2-yl)-1,3,4-thiadiazol-2-yl)piperidin-4-yl)methanone (**15**)

HPLC spectrum of [1,4'-bipiperidin]-1'-yl(1-(5-(5-nitrofuran-2-yl)-1,3,4-thiadiazol-2-yl)piperidin-4-yl)methanone (**15**)


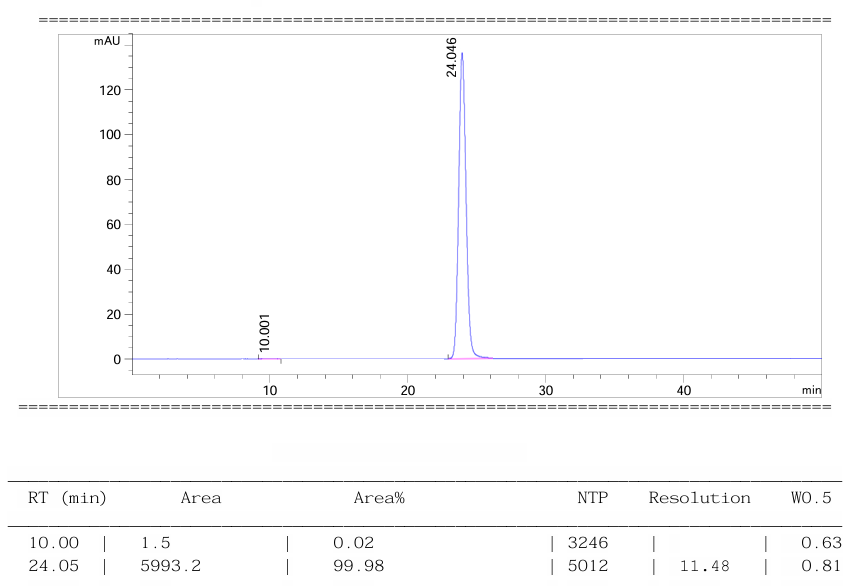


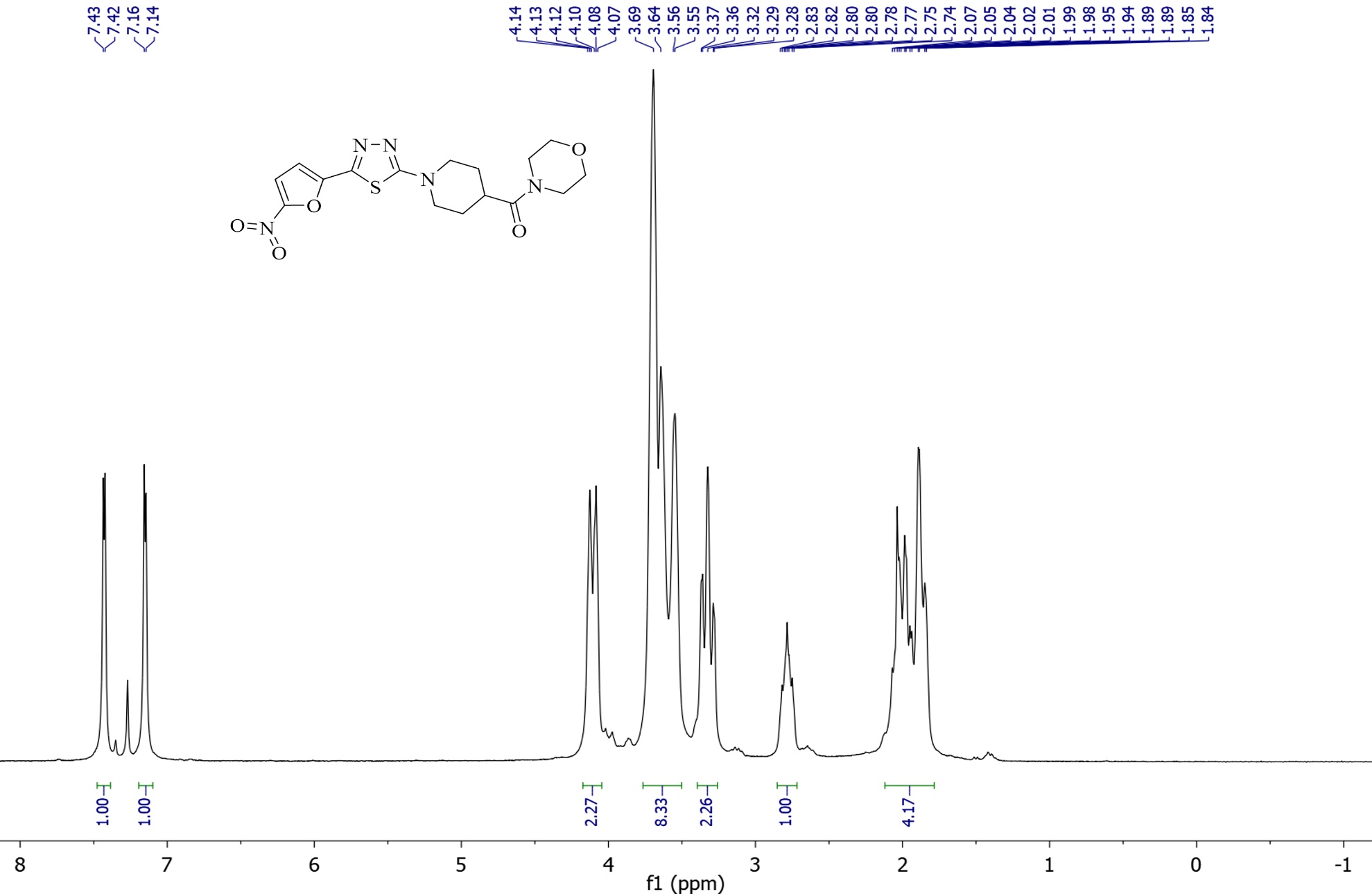
^1^H NMR spectrum of morpholino(1-(5-(5-nitrofuran-2-yl)-1,3,4-thiadiazol-2-yl)piperidin-4-yl)methanone (**16**)


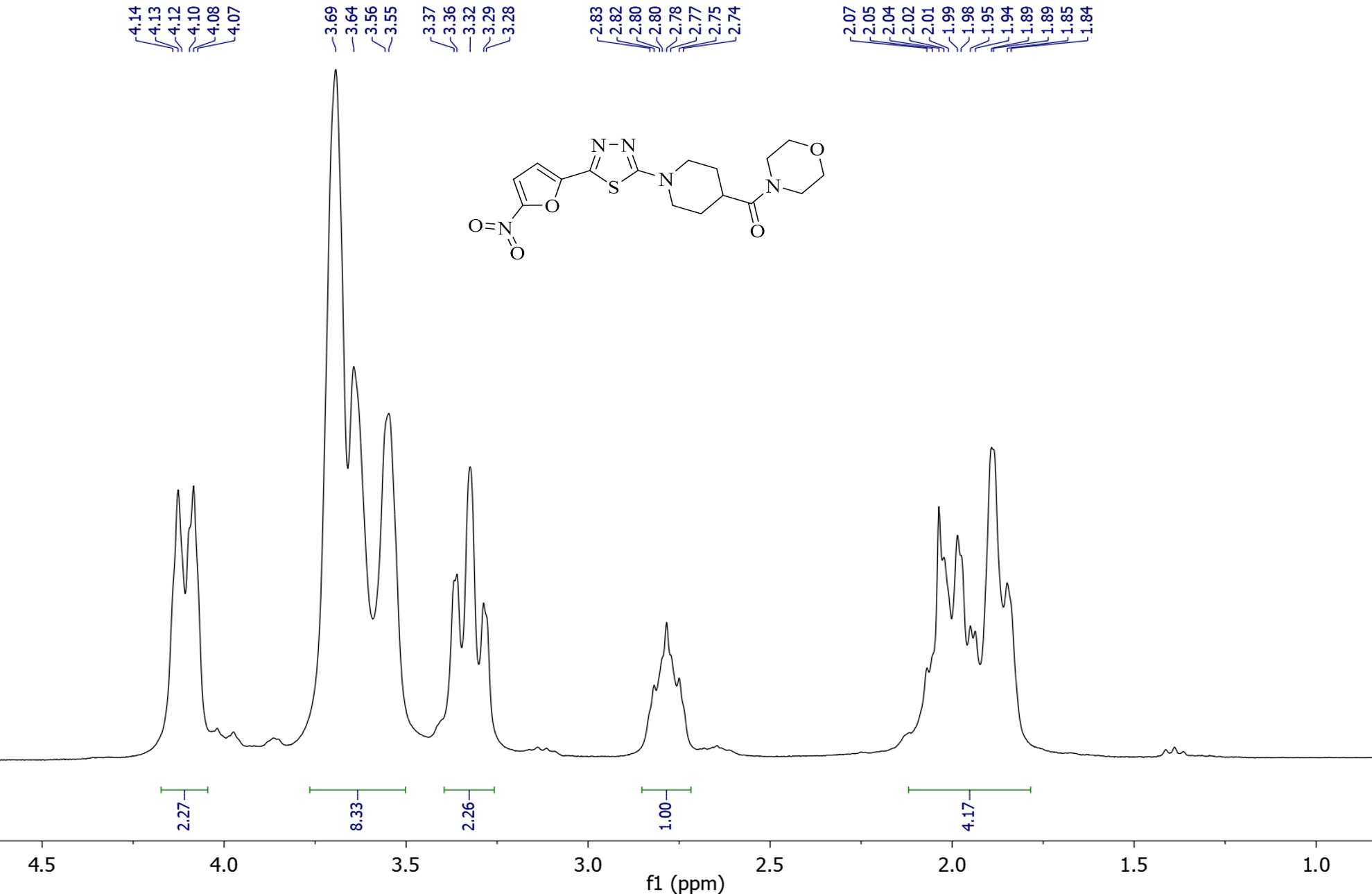
Expanded ^1^H NMR spectrum of morpholino(1-(5-(5-nitrofuran-2-yl)-1,3,4-thiadiazol-2-yl)piperidin-4-yl)methanone (**16**)


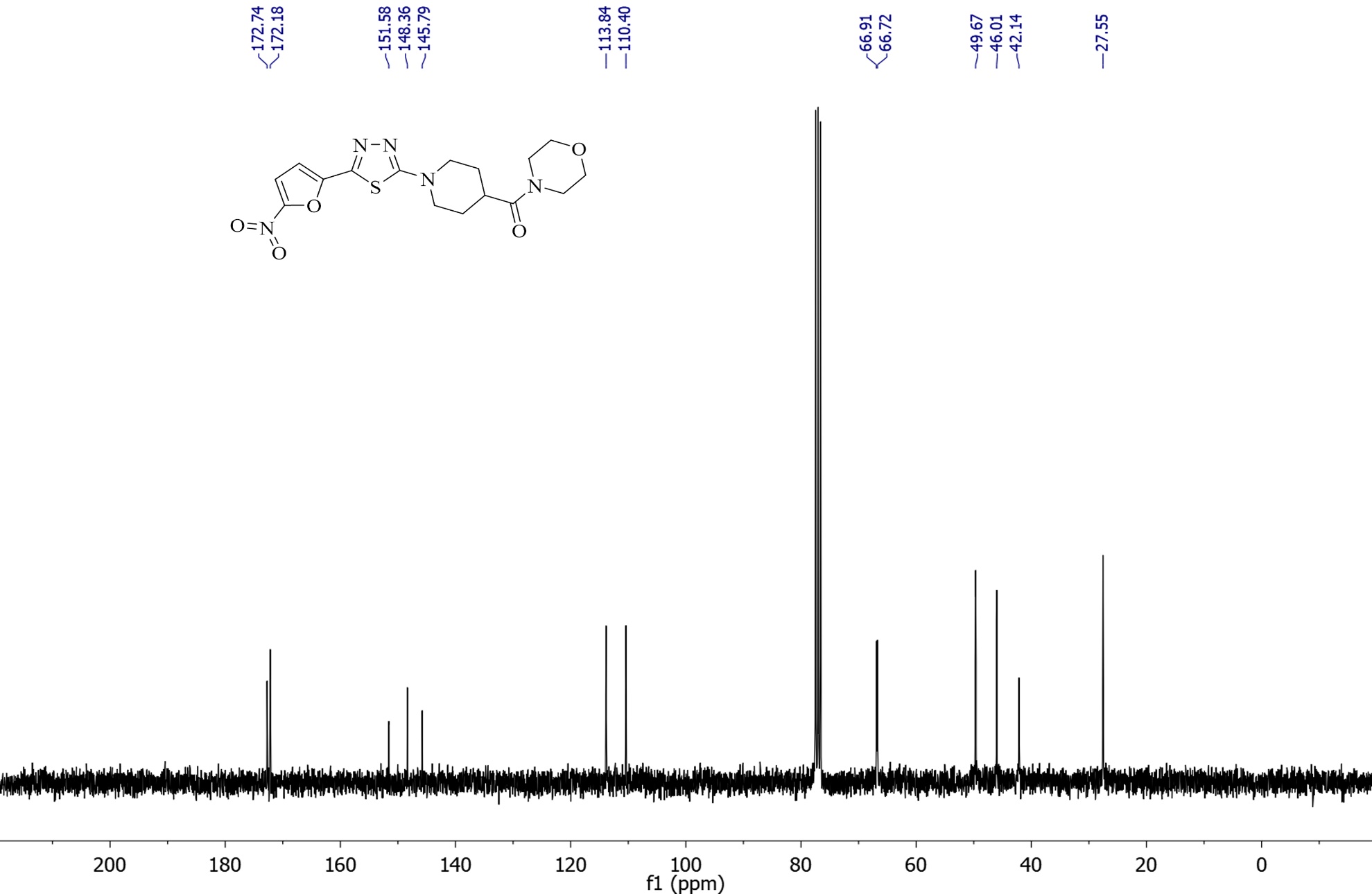
^13^C NMR spectrum of morpholino(1-(5-(5-nitrofuran-2-yl)-1,3,4-thiadiazol-2-yl)piperidin-4-yl)methanone (**16**)


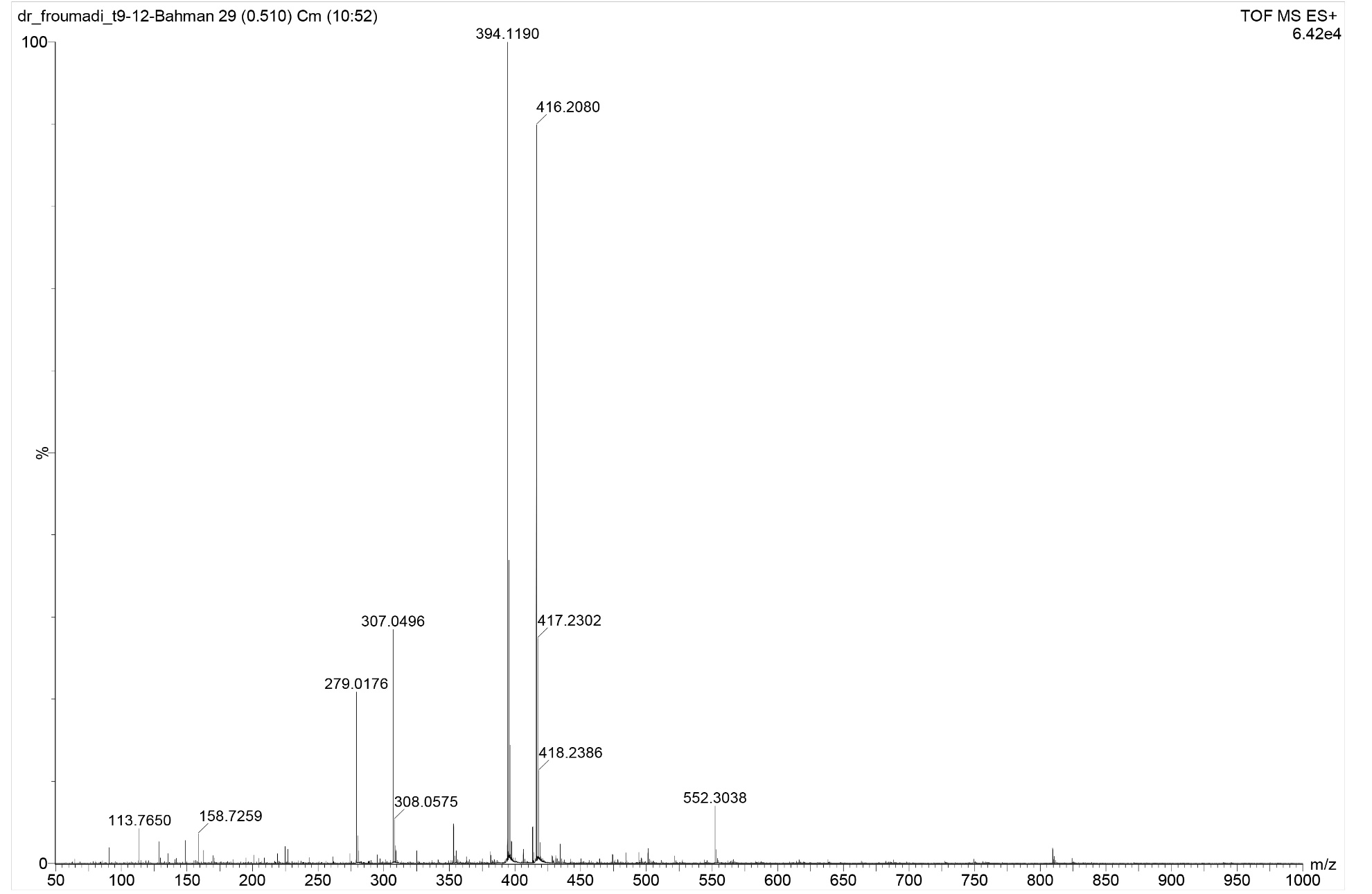
HRMS spectrum of morpholino(1-(5-(5-nitrofuran-2-yl)-1,3,4-thiadiazol-2-yl)piperidin-4-yl)methanone (**16**)

HPLC spectrum of morpholino(1-(5-(5-nitrofuran-2-yl)-1,3,4-thiadiazol-2-yl)piperidin-4-yl)methanone (**16**)

^
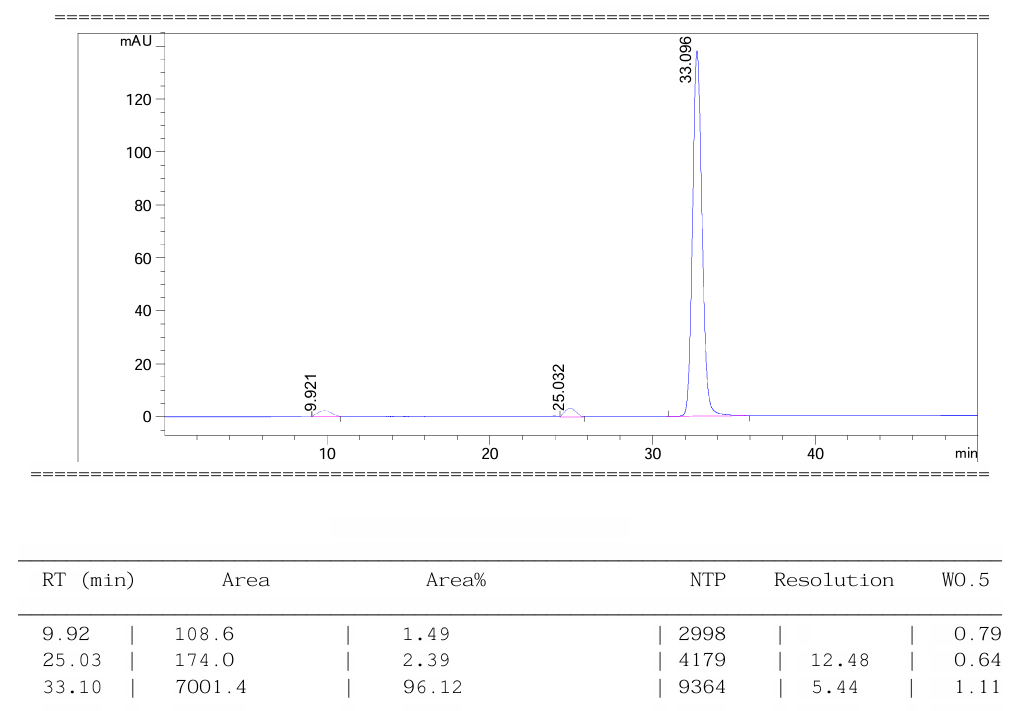
^


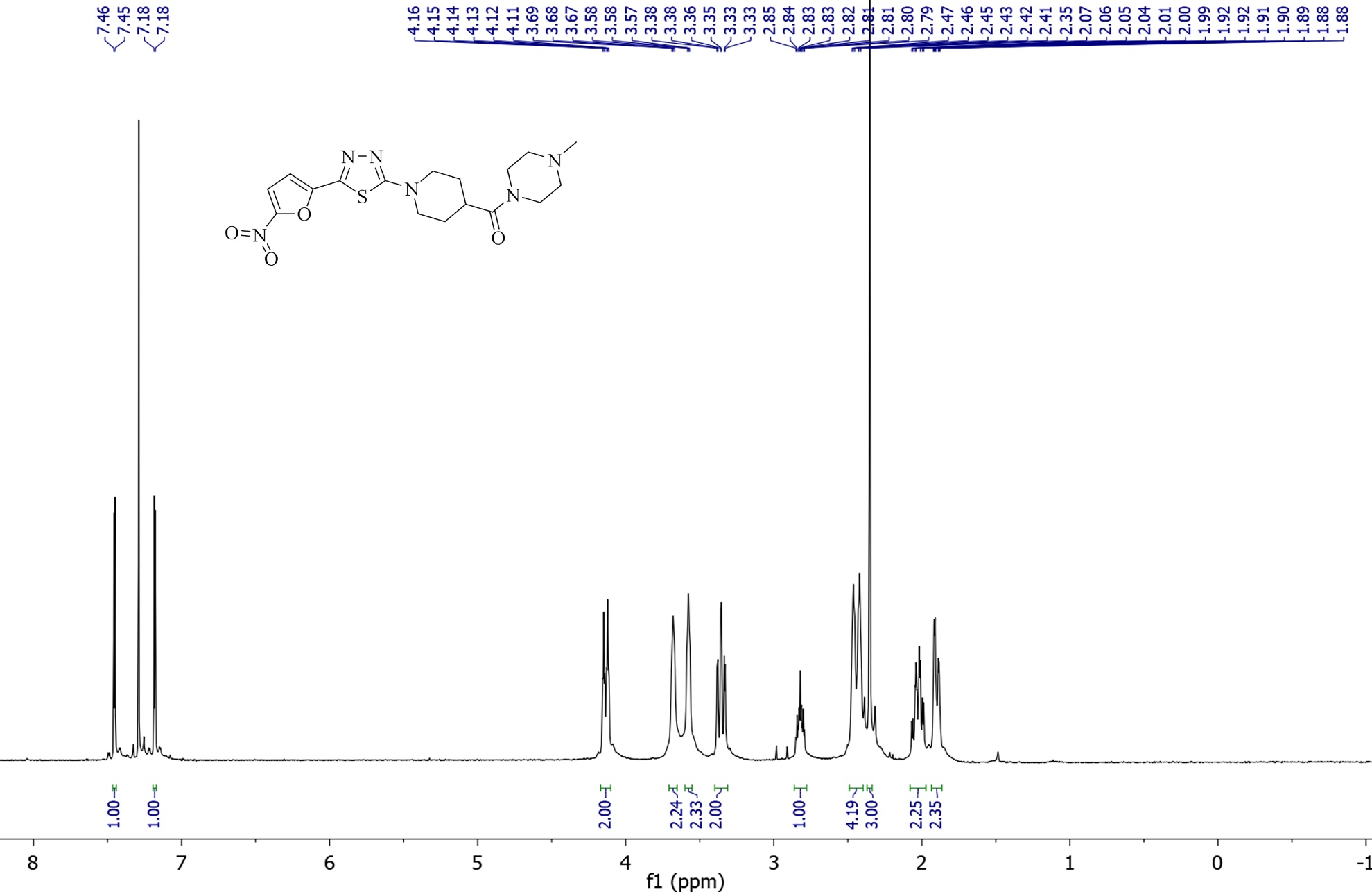
^1^H NMR spectrum of (4-methylpiperazin-1-yl)(1-(5-(5-nitrofuran-2-yl)-1,3,4-thiadiazol-2-yl)piperidin-4-yl)methanone (**17**)


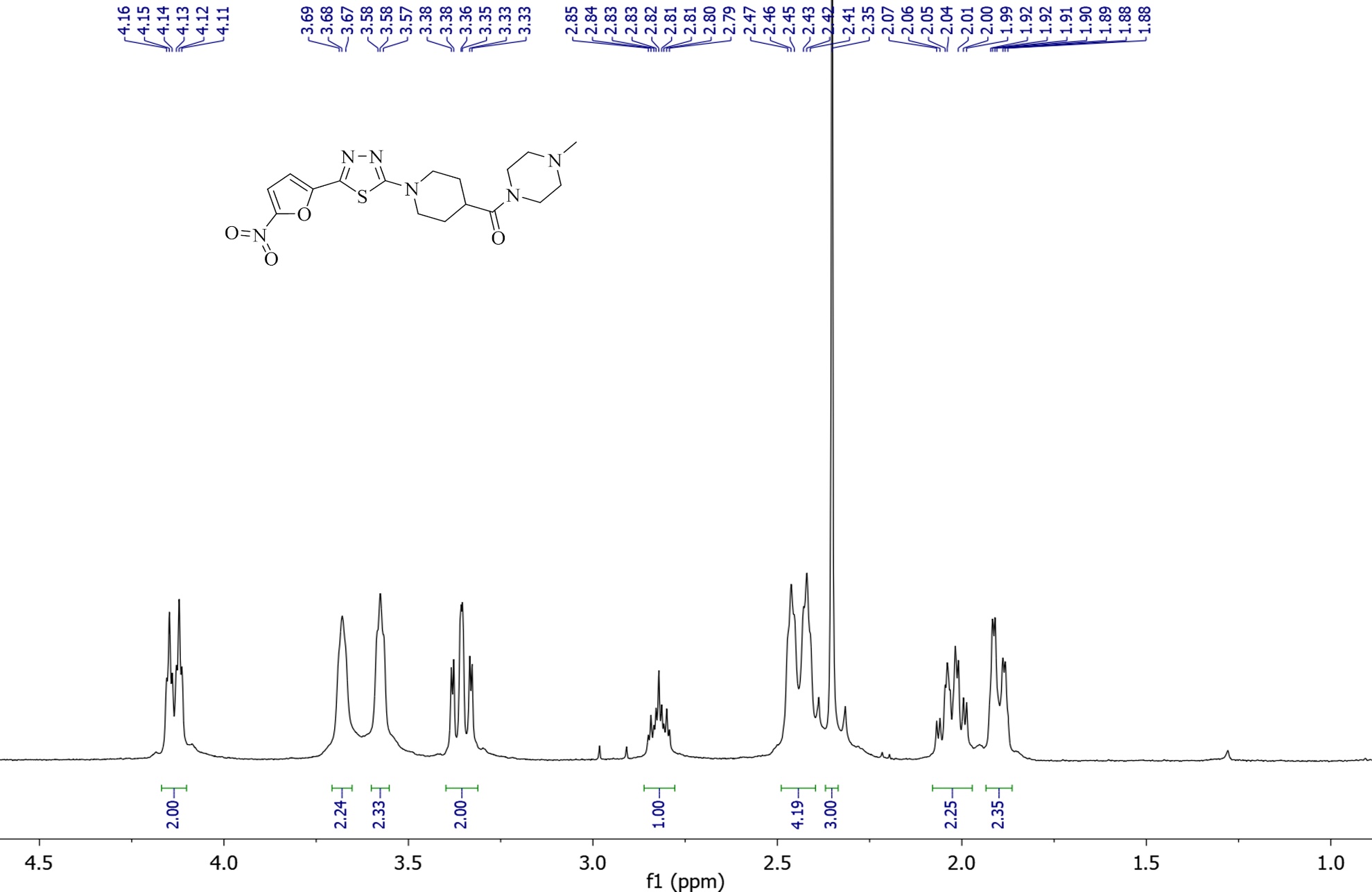
Expanded ^1^H NMR spectrum of (4-methylpiperazin-1-yl)(1-(5-(5-nitrofuran-2-yl)-1,3,4-thiadiazol-2-yl)piperidin-4-yl)methanone (**17**)


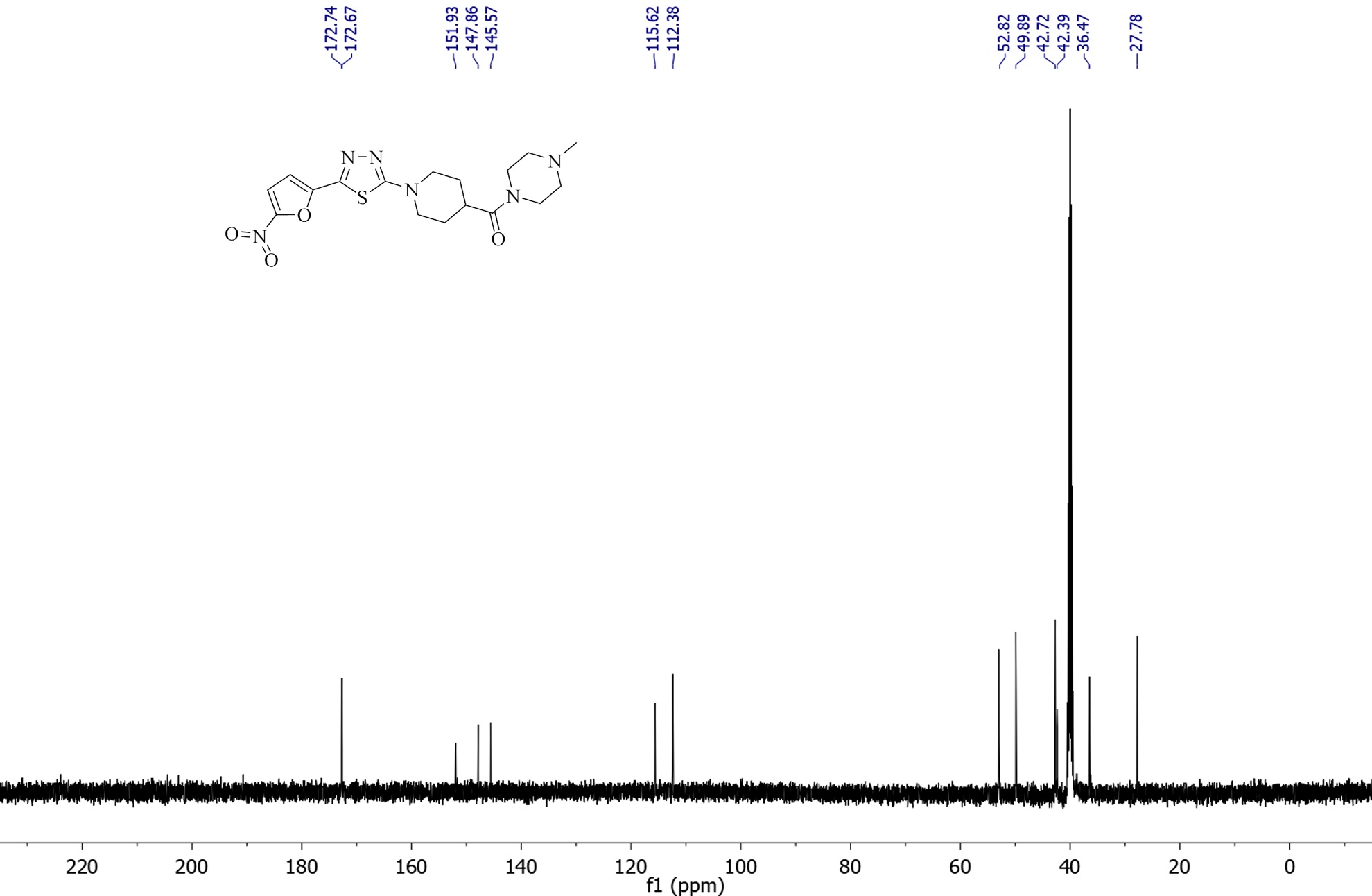
^13^C NMR spectrum of (4-methylpiperazin-1-yl)(1-(5-(5-nitrofuran-2-yl)-1,3,4-thiadiazol-2-yl)piperidin-4-yl)methanone (**17**)


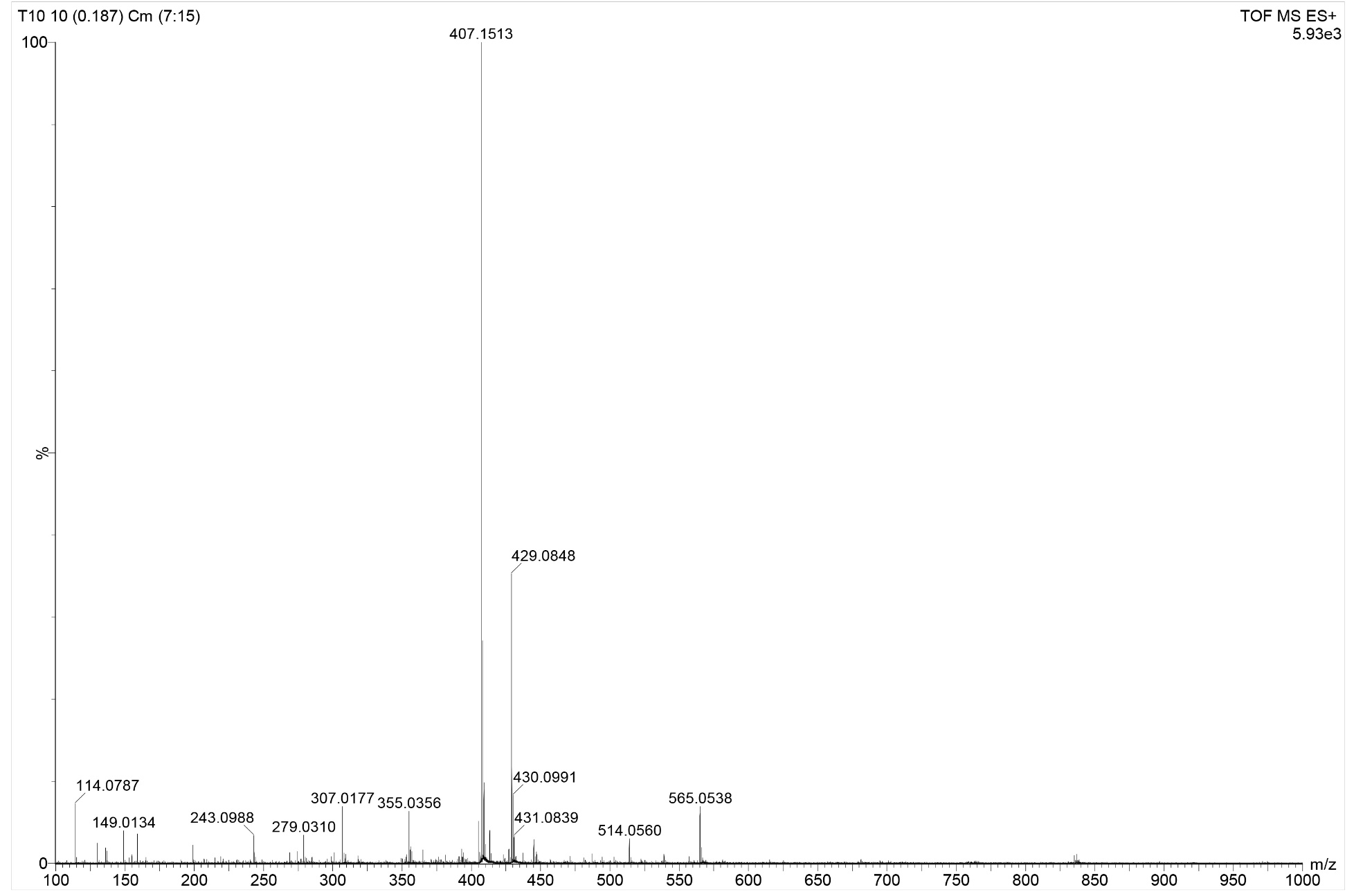
HRMS spectrum of (4-methylpiperazin-1-yl)(1-(5-(5-nitrofuran-2-yl)-1,3,4-thiadiazol-2-yl)piperidin-4-yl)methanone (**17**)

HPLC spectrum of (4-methylpiperazin-1-yl)(1-(5-(5-nitrofuran-2-yl)-1,3,4-thiadiazol-2-yl)piperidin-4-yl)methanone (**17**)

^
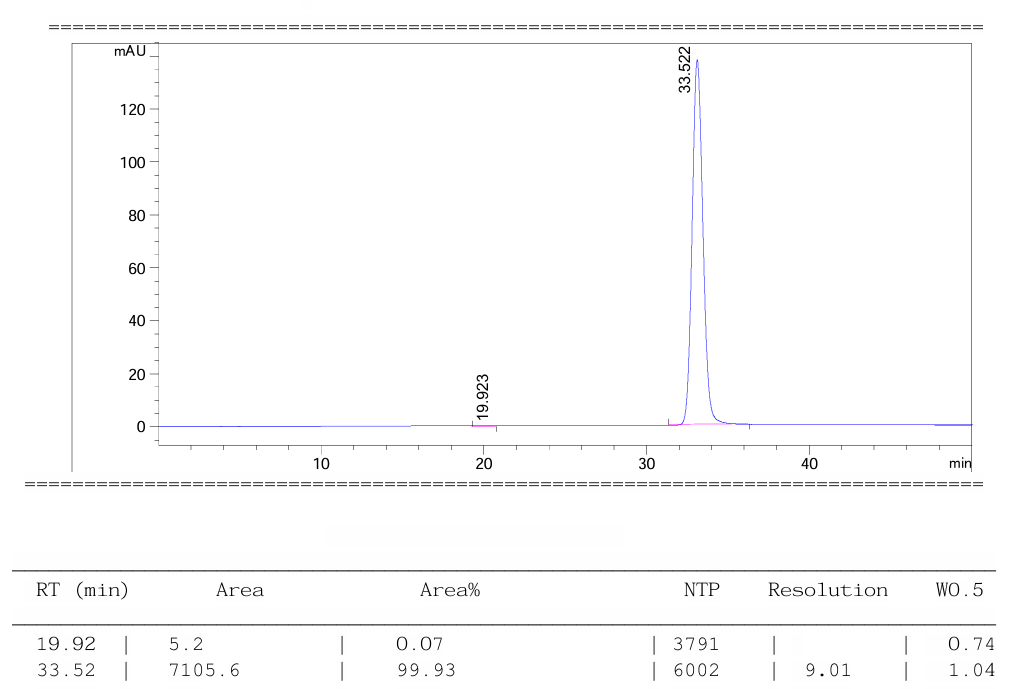
^


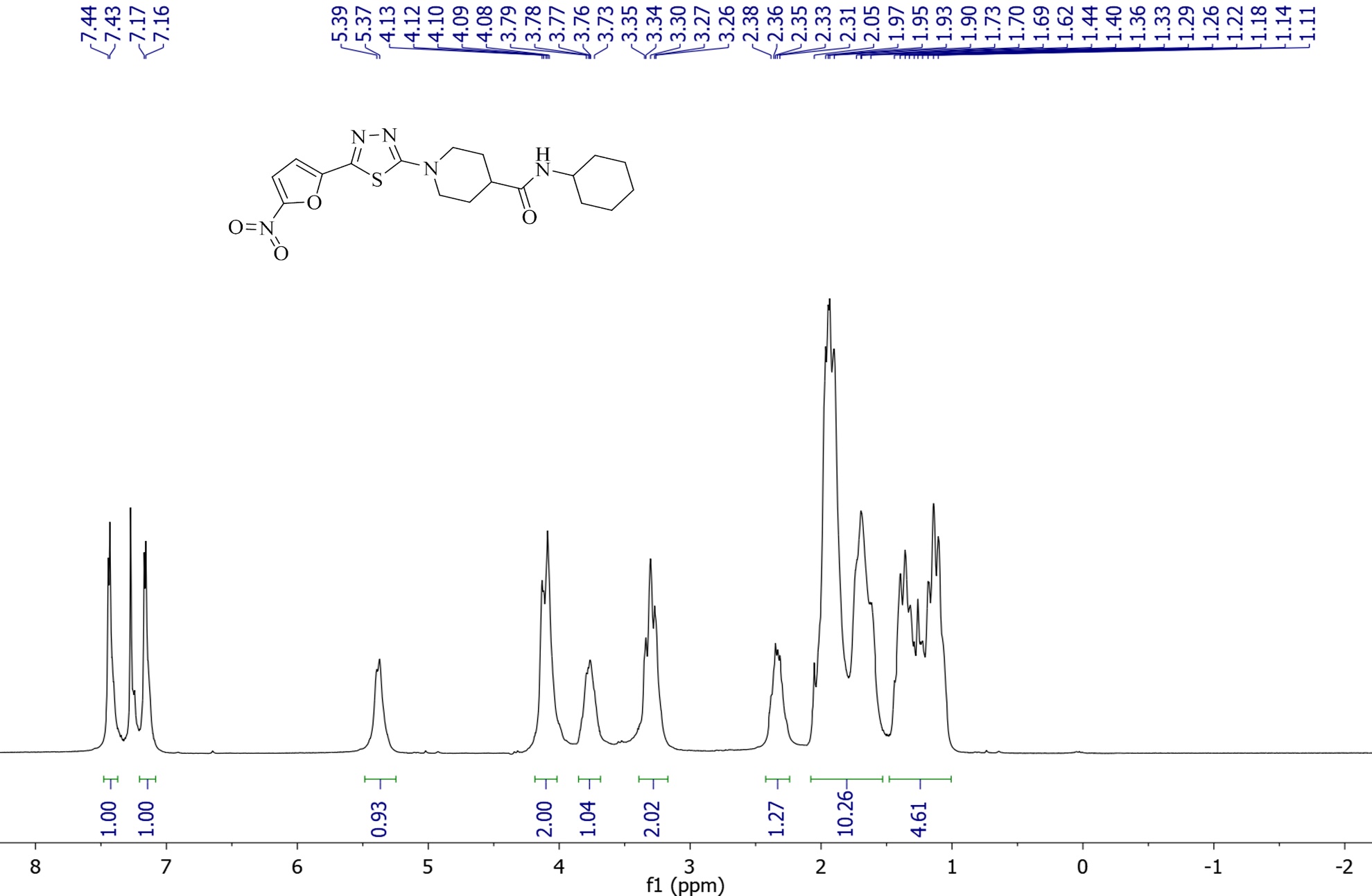
^1^H NMR spectrum of N-cyclohexyl-1-(5-(5-nitrofuran-2-yl)-1,3,4-thiadiazol-2-yl)piperidine-4-carboxamide (**18**)


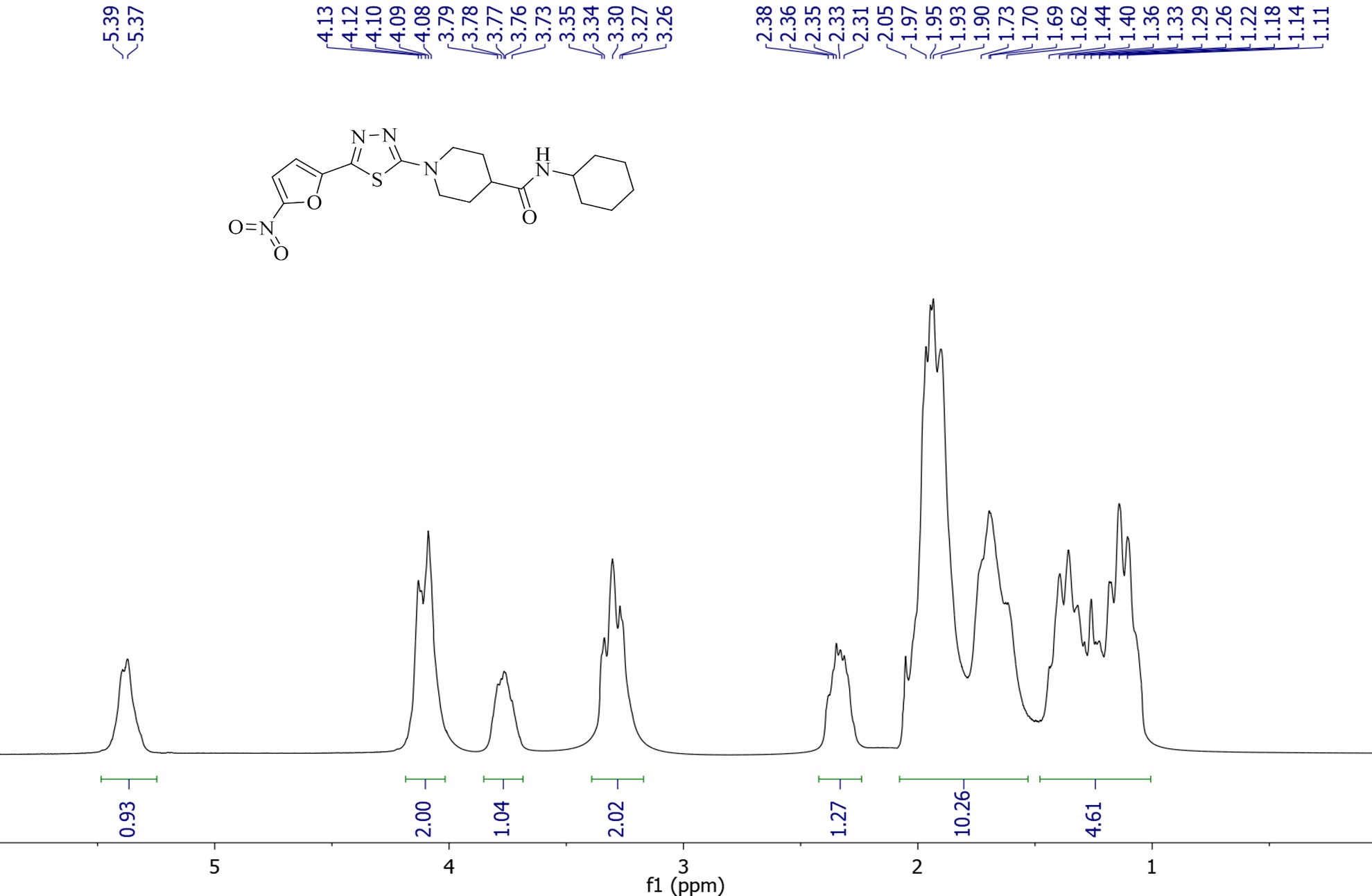
Expanded ^1^H NMR spectrum of *N*-cyclohexyl-1-(5-(5-nitrofuran-2-yl)-1,3,4-thiadiazol-2-yl)piperidine-4-carboxamide (**18**)


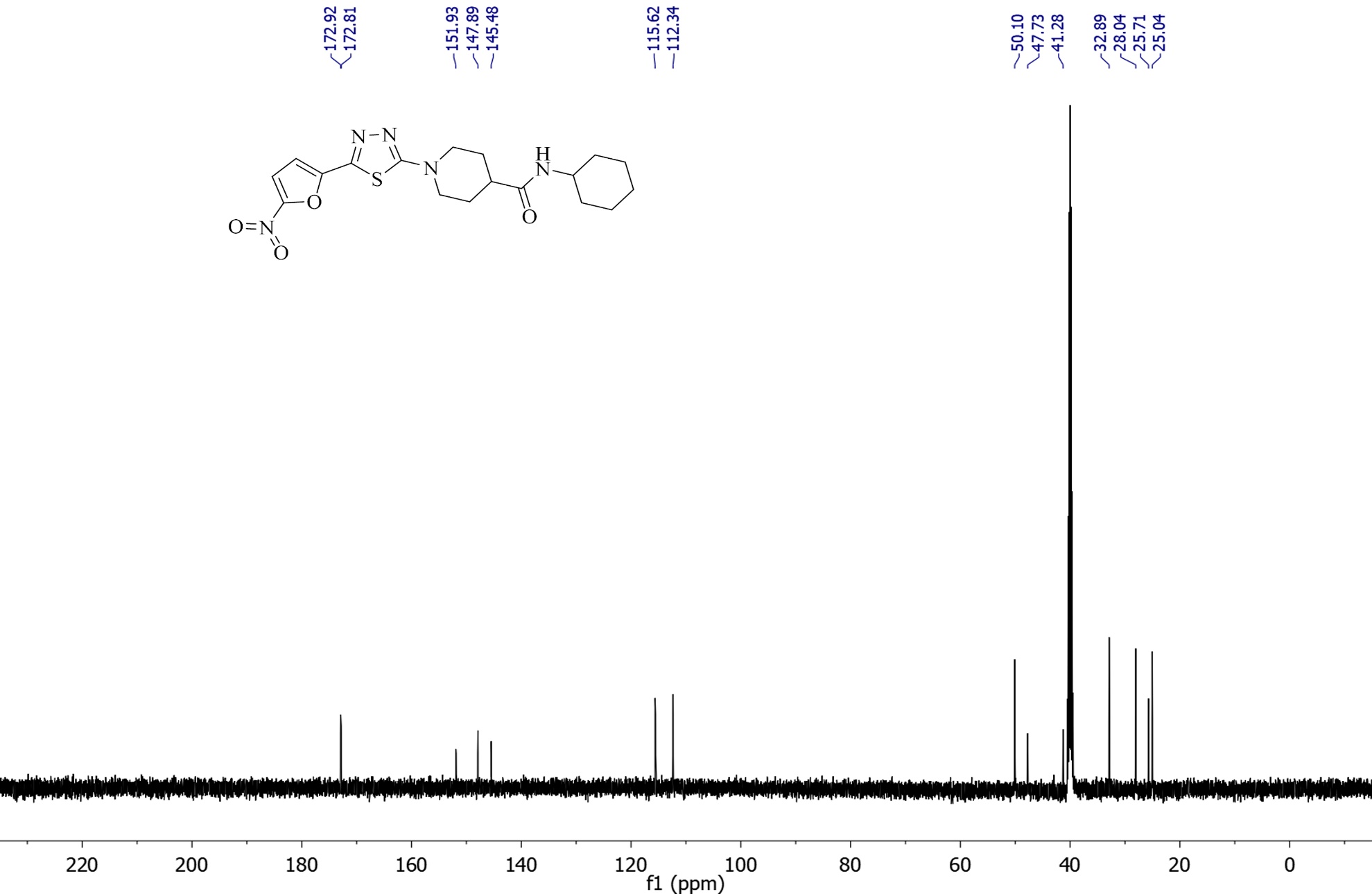
^13^C NMR spectrum of *N*-cyclohexyl-1-(5-(5-nitrofuran-2-yl)-1,3,4-thiadiazol-2-yl)piperidine-4-carboxamide (**18**)


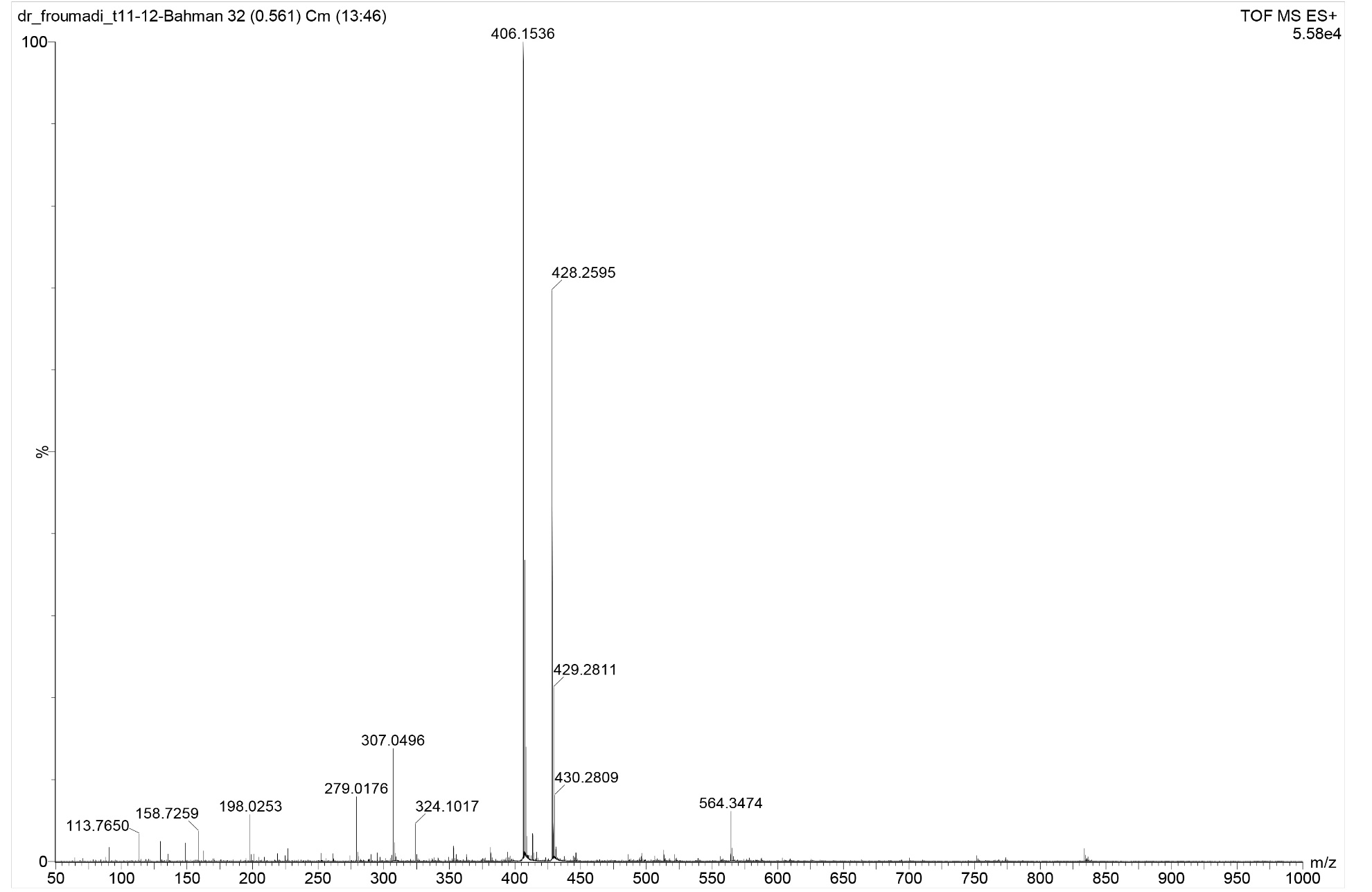
HRMS spectrum of *N*-cyclohexyl-1-(5-(5-nitrofuran-2-yl)-1,3,4-thiadiazol-2-yl)piperidine-4-carboxamide (**18**)

HPLC spectrum of N-cyclohexyl-1-(5-(5-nitrofuran-2-yl)-1,3,4-thiadiazol-2-yl)piperidine-4-carboxamide (**18**)

**
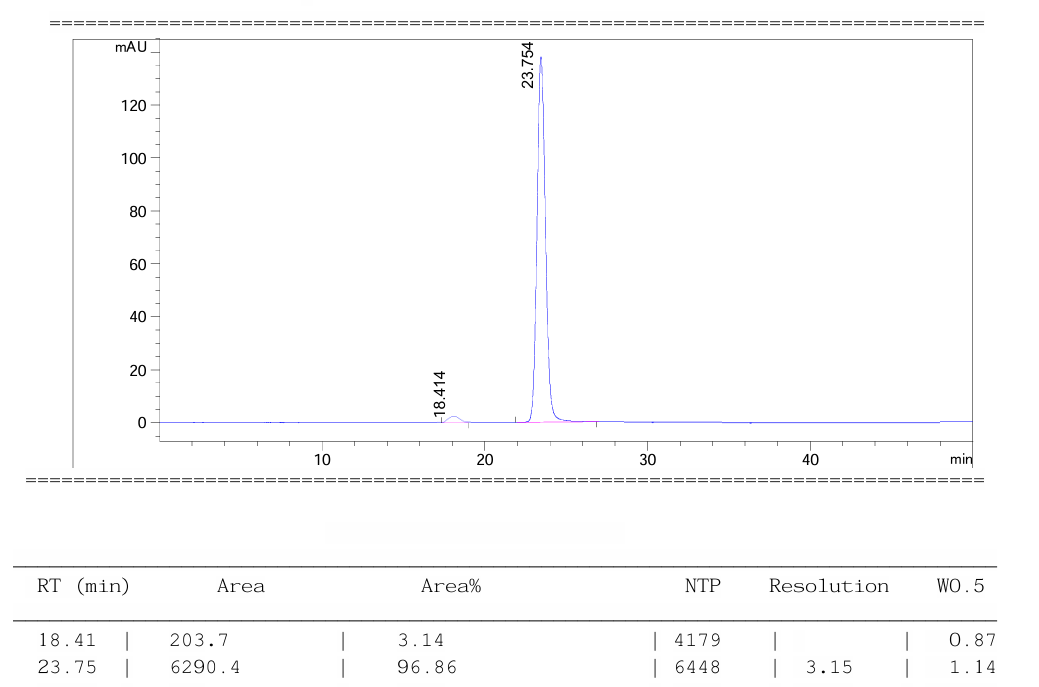
**


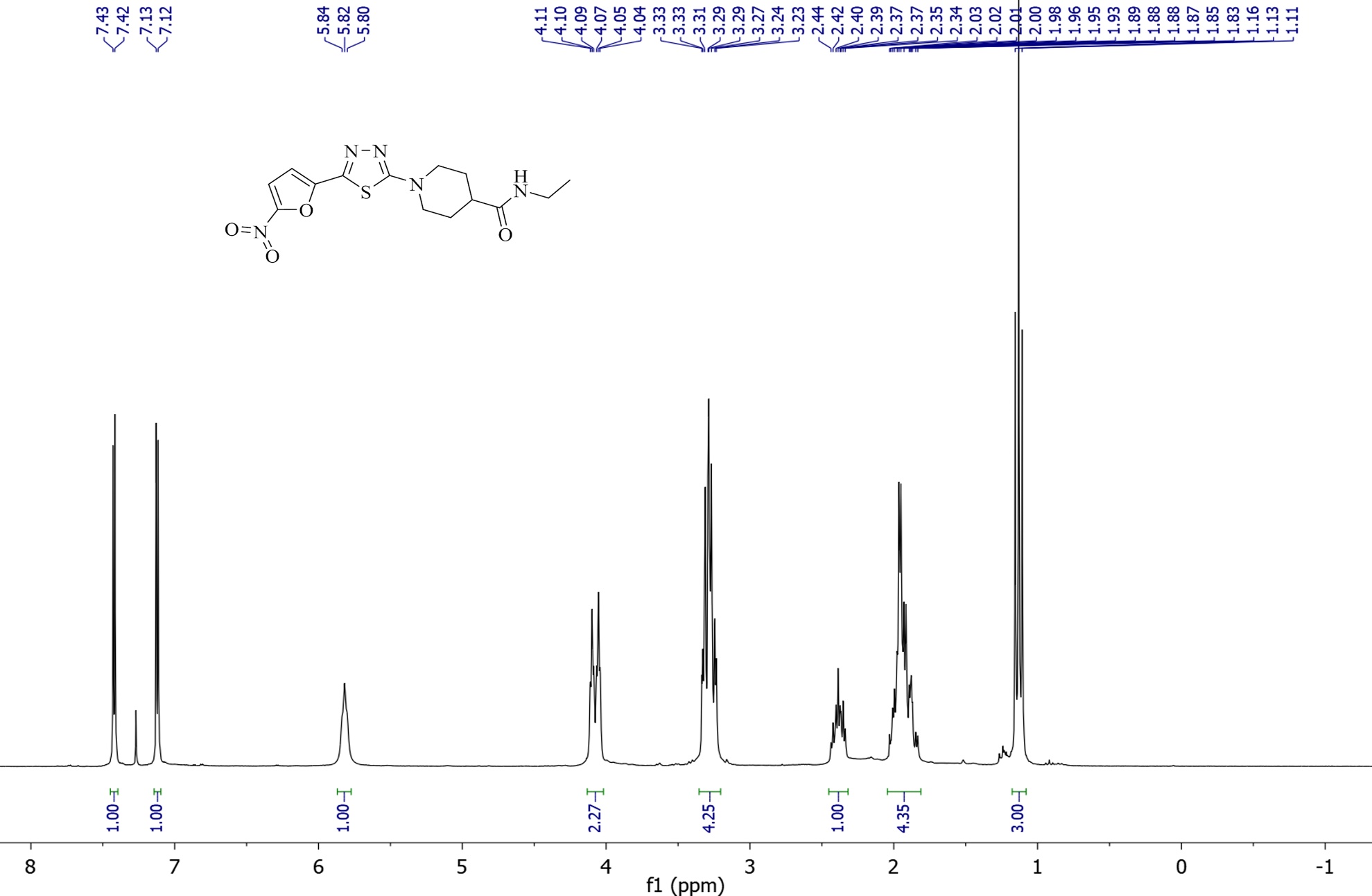
^1^H NMR spectrum of *N*-ethyl-1-(5-(5-nitrofuran-2-yl)-1,3,4-thiadiazol-2-yl)piperidine-4-carboxamide (**19**)


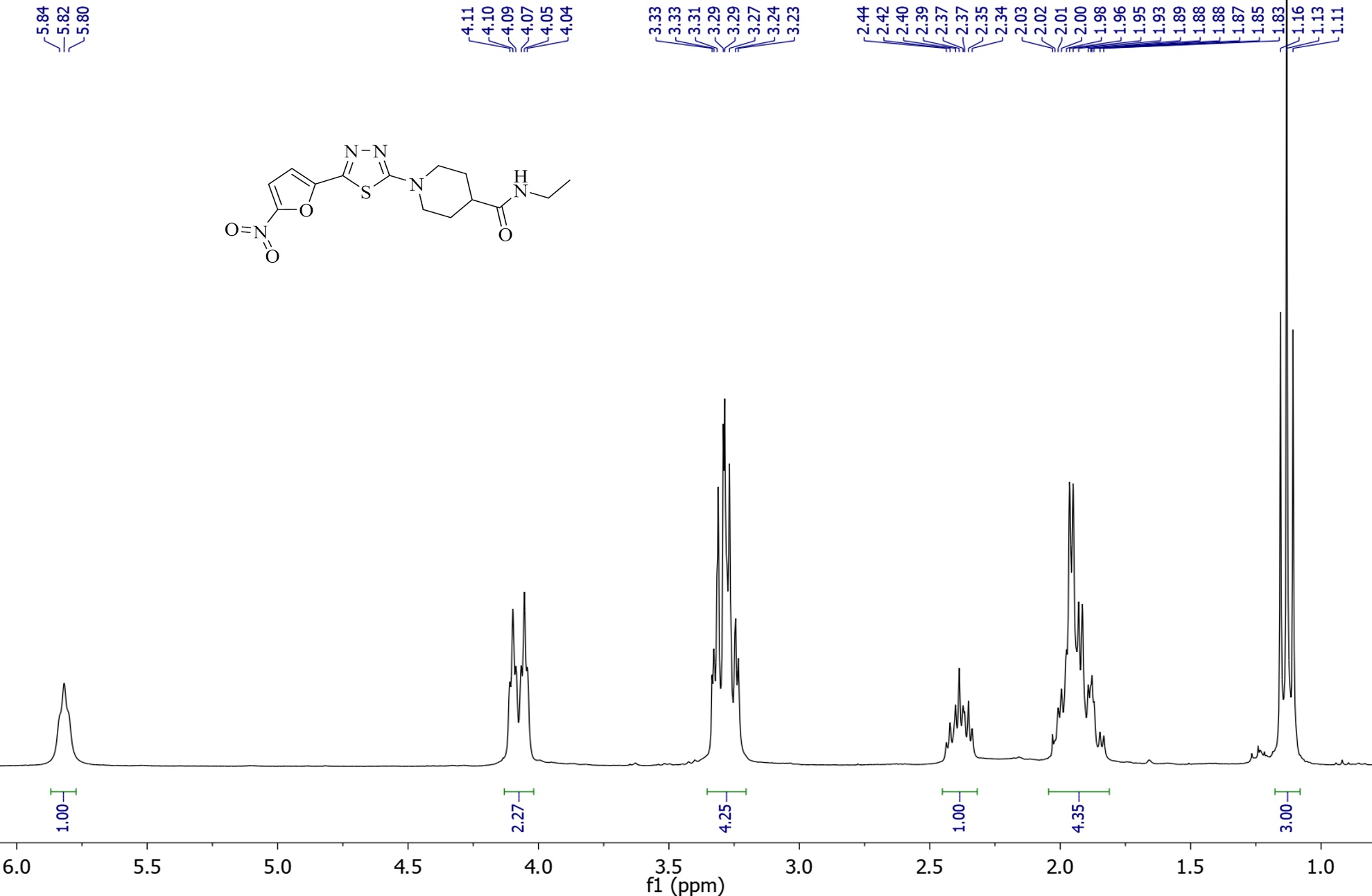
Expanded ^1^H NMR spectrum of *N*-ethyl-1-(5-(5-nitrofuran-2-yl)-1,3,4-thiadiazol-2-yl)piperidine-4-carboxamide (**19**)


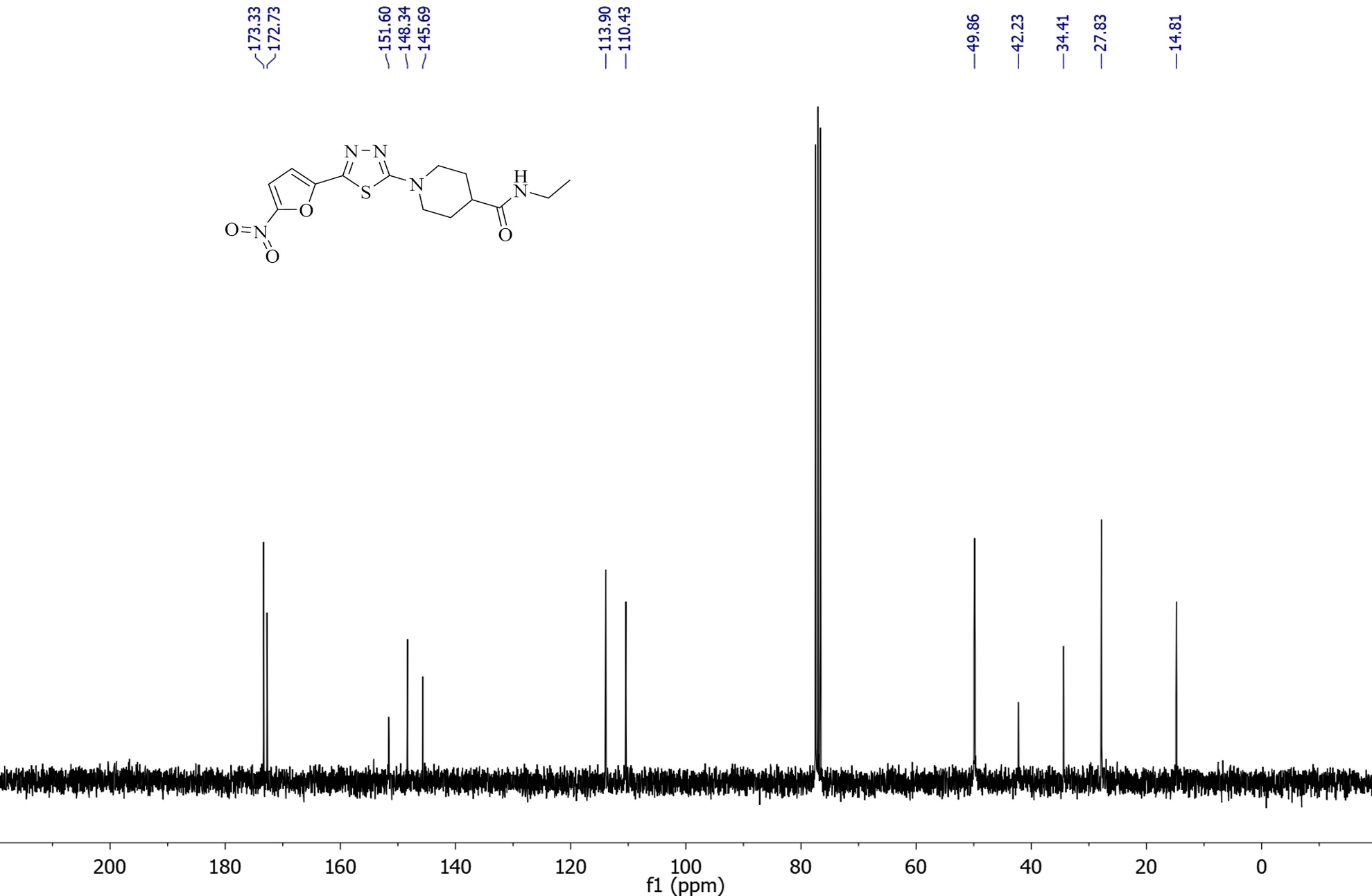
^13^C NMR spectrum of *N*-ethyl-1-(5-(5-nitrofuran-2-yl)-1,3,4-thiadiazol-2-yl)piperidine-4-carboxamide (**19**)


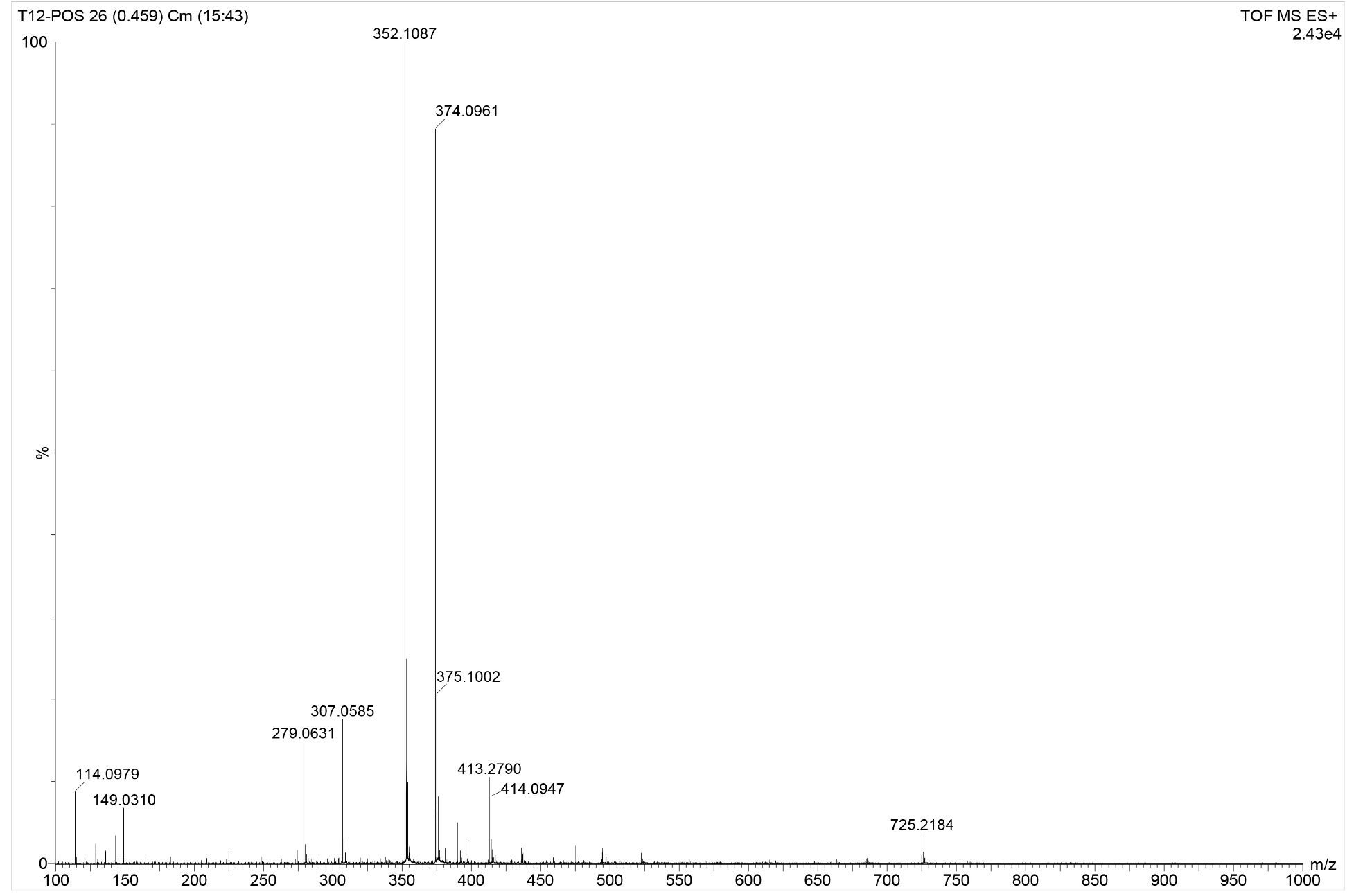
HRMS spectrum of *N*-ethyl-1-(5-(5-nitrofuran-2-yl)-1,3,4-thiadiazol-2-yl)piperidine-4-carboxamide (**19**)

HPLC spectrum of N-ethyl-1-(5-(5-nitrofuran-2-yl)-1,3,4-thiadiazol-2-yl)piperidine-4-carboxamide (**19**)

^
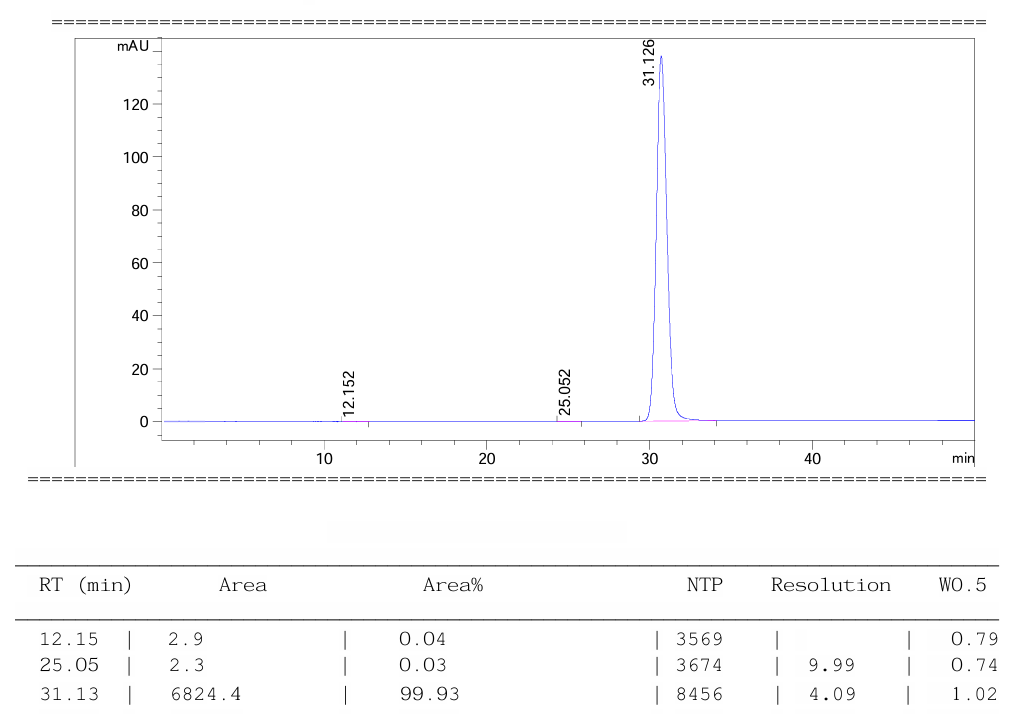
^


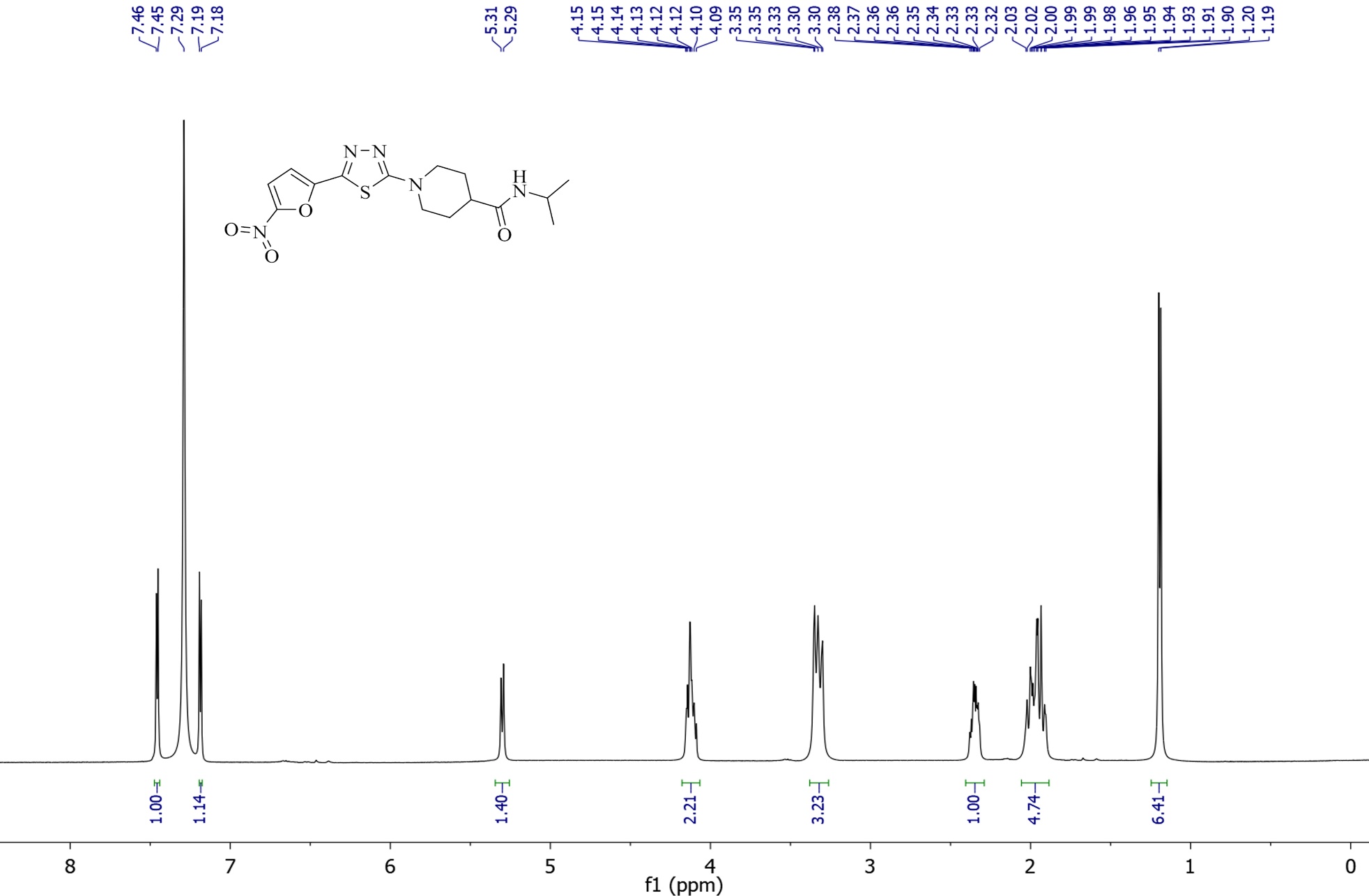
^1^H NMR spectrum of *N*-isopropyl-1-(5-(5-nitrofuran-2-yl)-1,3,4-thiadiazol-2-yl)piperidine-4-carboxamide (**20**)


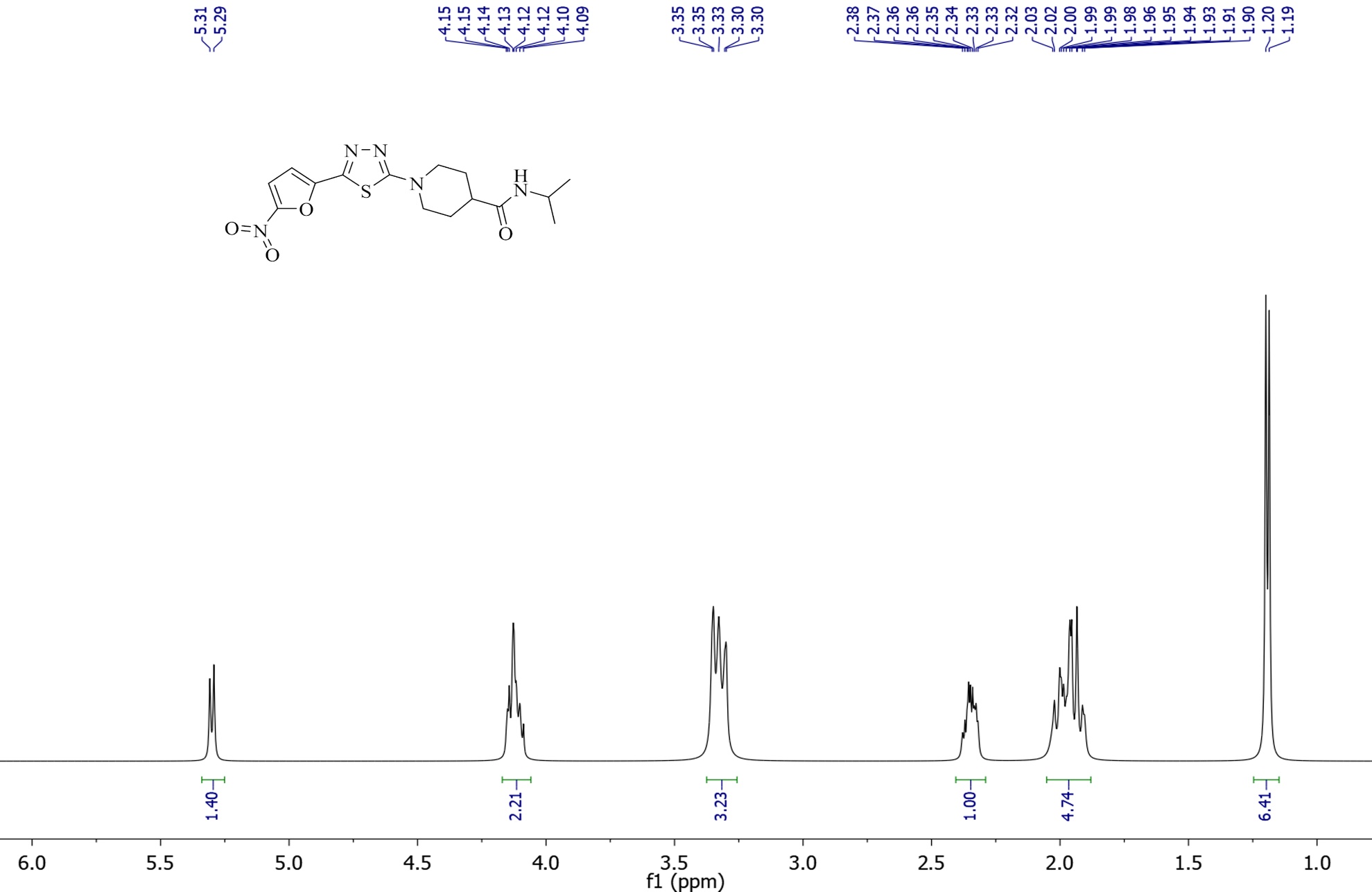
Expanded ^1^H NMR spectrum of *N*-isopropyl-1-(5-(5-nitrofuran-2-yl)-1,3,4-thiadiazol-2-yl)piperidine-4-carboxamide (**20**)


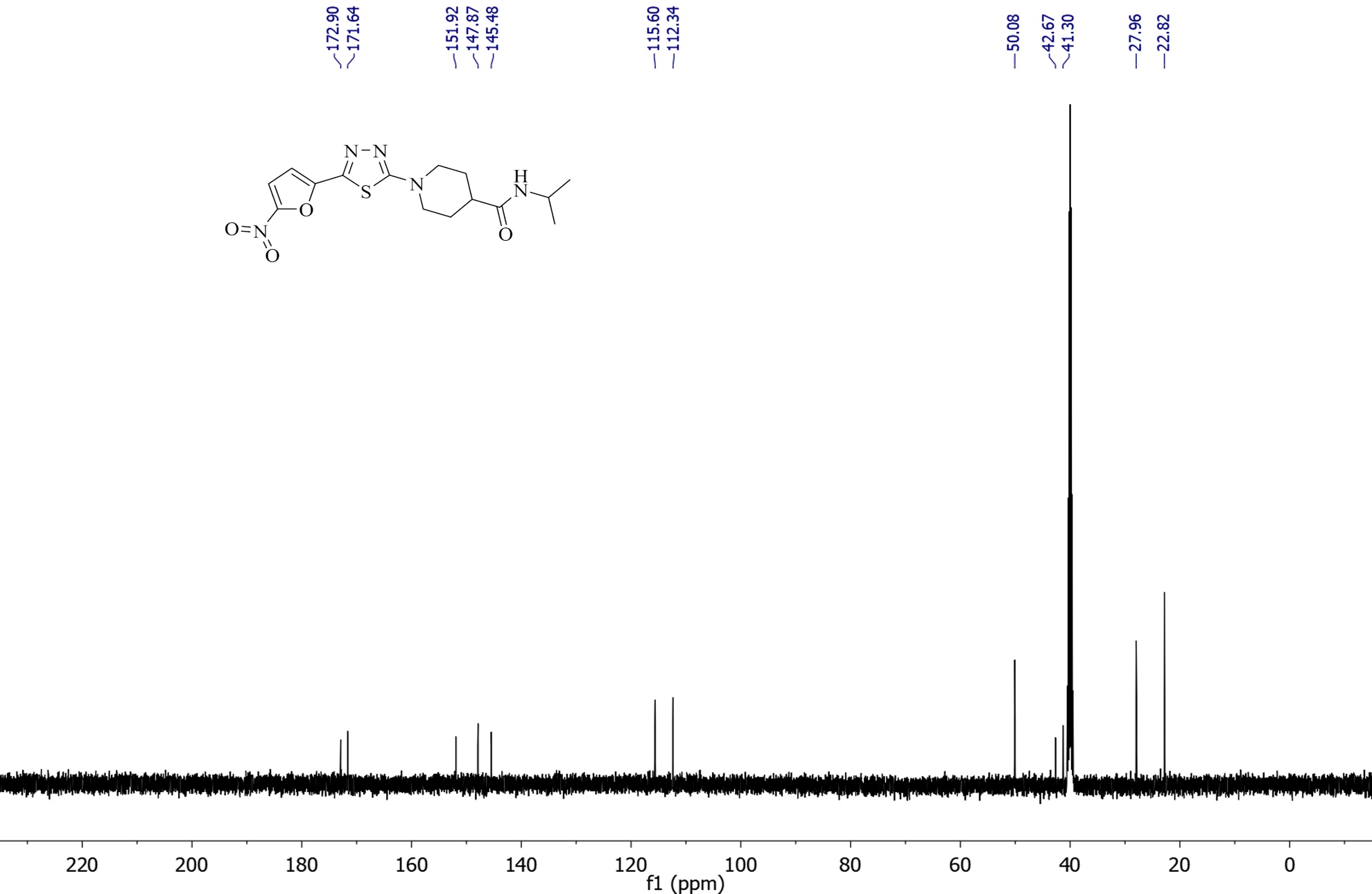
^13^C NMR spectrum of *N*-isopropyl-1-(5-(5-nitrofuran-2-yl)-1,3,4-thiadiazol-2-yl)piperidine-4-carboxamide (**20**)


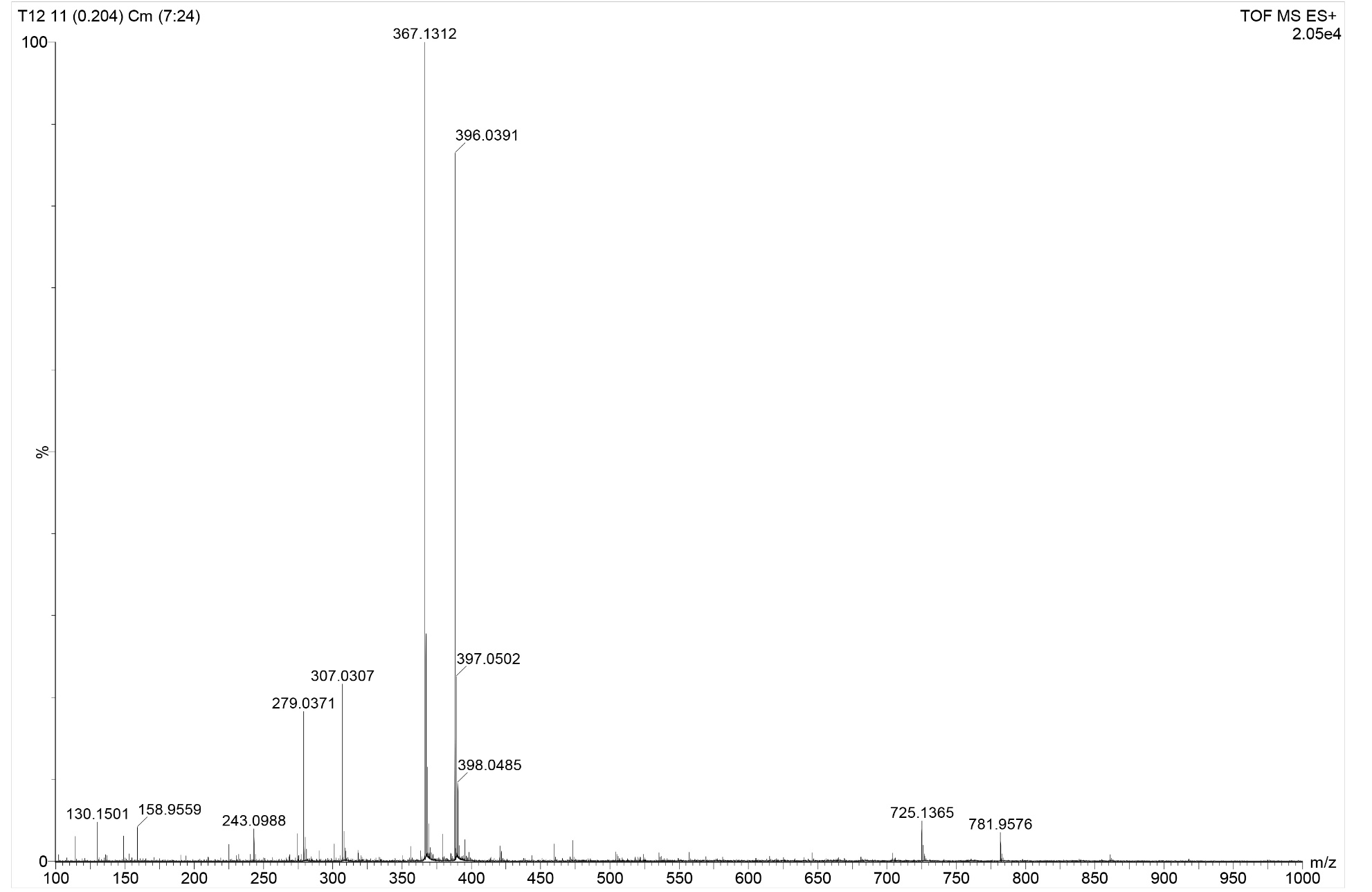
HRMS spectrum of *N*-isopropyl-1-(5-(5-nitrofuran-2-yl)-1,3,4-thiadiazol-2-yl)piperidine-4-carboxamide (**20**)

HPLC spectrum of N-isopropyl-1-(5-(5-nitrofuran-2-yl)-1,3,4-thiadiazol-2-yl)piperidine-4-carboxamide (**20**)

**
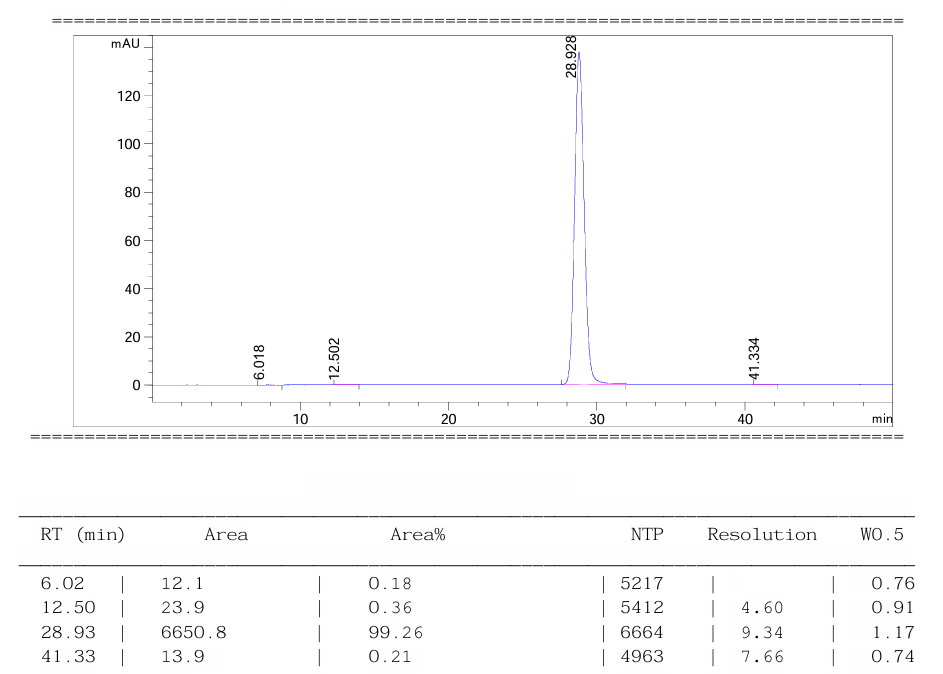
**
